# QSAR Accelerated Discovery of Potent Ice Recrystallization Inhibitors

**DOI:** 10.1038/srep26403

**Published:** 2016-05-24

**Authors:** Jennie G. Briard, Michael Fernandez, Phil De Luna, Tom. K. Woo, Robert N. Ben

**Affiliations:** 1 Department of Chemistry and Biomolecular Sciences University of Ottawa, 10 Marie Curie, Ottawa, Ontario, K1N 6N5, Canada

## Abstract

Ice recrystallization is the main contributor to cell damage and death during the cryopreservation of cells and tissues. Over the past five years, many small carbohydrate-based molecules were identified as ice recrystallization inhibitors and several were shown to reduce cryoinjury during the cryopreservation of red blood cells (RBCs) and hematopoietic stems cells (HSCs). Unfortunately, clear structure-activity relationships have not been identified impeding the rational design of future compounds possessing ice recrystallization inhibition (IRI) activity. A set of 124 previously synthesized compounds with known IRI activities were used to calibrate 3D-QSAR classification models using GRid INdependent Descriptors (GRIND) derived from DFT level quantum mechanical calculations. Partial least squares (PLS) model was calibrated with 70% of the data set which successfully identified 80% of the IRI active compounds with a precision of 0.8. This model exhibited good performance in screening the remaining 30% of the data set with 70% of active additives successfully recovered with a precision of ~0.7 and specificity of 0.8. The model was further applied to screen a new library of aryl-alditol molecules which were then experimentally synthesized and tested with a success rate of 82%. Presented is the first computer-aided high-throughput experimental screening for novel IRI active compounds.

Cryopreservation is an attractive strategy permitting the long-term storage of biological materials by using very low sub-zero temperatures (>−190 °C). At these temperatures, all biochemical processes are stopped and cells can be stored for extended periods of time. However, the cryoinjury (poor post-thaw viabilities and decreased functionality) associated with the process of cryopreservation continues to be a significant problem^1^,^2^, and is quickly becoming the major bottle neck in the clinical deployment of novel cell therapies, regenerative medicine applications^3^ and tissue engineering^4^.

Cryoinjury during freezing and thawing is a complex issue^2^,^5^,^6^,^7^,^8^,^9^. However, the major source of cryoinjury occurs as a result of ice formation and subsequent mechanical damage to cell membranes^5^,^10^,^11^,^12^. Current clinically used cryoprotectants function by stabilizing cellular membranes and minimizing osmotic imbalances by replacing water within the cell^13^. Interestingly, there are no cryoprotectants in current use that are capable of controlling ice growth or recrystallization even though this causes cellular injury. Consequently, non-toxic molecules capable of inhibiting ice recrystallization constitute a new class of cryoprotectants that may meet the current needs of modern cellular therapy.

Our laboratory has previously demonstrated that several small carbohydrate-based molecules are potent inhibitors of ice recrystallization (IRIs) and protect hematopoietic stem cells (HSCs)^14^,^15^ and human red blood cells (RBCs)^16^ from cryoinjury associated with ice recrystallization. Subsequent to this, we have identified several structurally different classes of small molecules that are very effective inhibitors of ice recrystallization (Fig. 1)^17^,^18^,^19^,^20^,^21^.

In order to identify these five specific classes of IRIs, hundreds of other molecules were synthesized and their IRI activity characterized before *in vitro* studies were performed. The IRI activity is measured as a percentage of the mean grain size of the ice crystals formed relative to those in a solution of phosphate buffered saline (PBS) in a splat cooling assay^22^. This is a time-consuming process. While the exact mechanism by which these small molecules inhibit ice recrystallization is currently unknown, key structural attributes required for inhibition of this process are also not known. This is unfortunate as this makes it very difficult to rationally design *de novo* inhibitors.

For instance four different pyranoses are shown in Fig. 2. Prior work in our laboratory has shown that of typical simple monosaccharides d-galactose is the most potent inhibitor of ice recrystallization^17^. In fact, changing the stereochemistry of the C4 hydroxyl group has a dramatic effect upon the IRI activity as evidenced by the fact that d-glucose is 20% less active. This observation is attributed to the fact that d-galactose is more hydrated than d-glucose (hydration parameters obtained from literature)^23^,^24^. However, when β-PMP-Gal is synthesized and tested for IRI activity at 22 mM it is a significantly poorer inhibitor of ice recrystallization when compared to β-PMP-Glc at the same concentration. Structure-function correlations of this nature make it very difficult to rationally design new and improved small molecule ice recrystallization inhibitors and nicely illustrate the complexity of the problem. Consequently, we sought to use *quantitative structure activity relationship* (*QSAR*) models to accelerate development of new IRI active compounds. QSAR models relate the chemical structure to a specific activity or property using regression models of one or more structural descriptor variables. QSAR modelling is an established tool in the pharmaceutical industry where it has been well documented to reduce drug discovery timelines^25^.

There are several potential challenges to applying QSAR modeling to the problem of small molecule cryoprotectants. First, distinctly different classes of IRI active molecules have been discovered (Fig. 1), each containing highly active IRI molecules. Furthermore, as illustrated in Fig. 2, there are dramatic changes in IRI activity associated with seemingly subtle structural changes, including changes in stereochemistry.

In this work, we utilize the experimental IRI data from a diverse set of 124 molecules, some of which are shown in Fig. 3 (a complete list is provided in the Supplementary Information) to build a QSAR model to classify molecules as IRI active or inactive. This model was then used to classify the IRI activity of a set of 29 aryl-alditol structures that had not yet been synthesized. A subset of 17 of these compounds, 11 predicted to be IRI active, were then synthesized and their IRI activity measured. The ability of the 3D-QSAR models to enrich the sampling of IRI active molecules, and ultimately accelerate the discovery of active compounds was assessed.

## Methods

**Figure 3:**
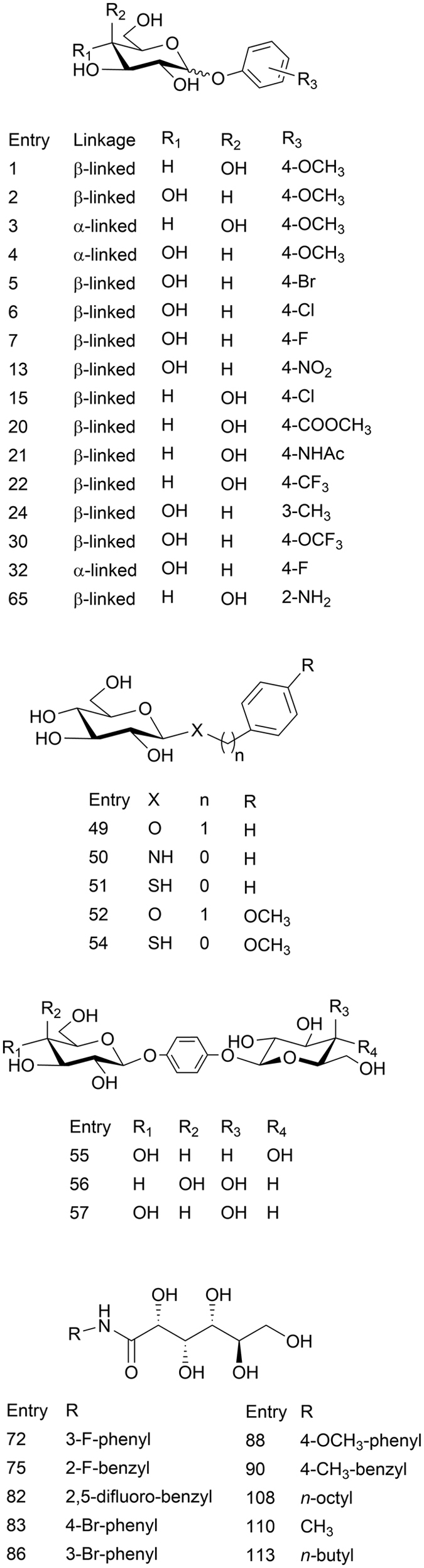
Select diverse structures from the 124 compounds used to develop the 3D-QSAR model. A full list of all 124 compounds can be found in the Supporting Info.

The set of 124 structures were synthesized and characterized, the details of which are described the Supplementary Information. A standard splat cooling assay^22^ was then used to evaluate each compound’s IRI activity. Briefly, the compound of interest is dissolved in PBS, and frozen in the form of a wafer. The wafer is then held at −6.4 °C for thirty minutes, during which ice recrystallization occurs. Subsequently, photos of the ice crystals are taken and an image analysis software^26^ is used to determine the average ice crystal size. Twelve ice crystals from three separate photos taken from three samples is used to calculate a total average size of 108 ice crystals. This average is compared to the average ice crystal size obtained from a PBS positive control for ice recrystallization to obtain a percent mean grain size (%MGS) relative to PBS. A selected subset of the structures previously synthesized and assessed for IRI activity is given in Fig. 3.

The structures were first modelled in three-dimensions using the Spartan program^27^ and optimized using the Merck Molecular Force Field (MMFF)^28^. Next, a conformational search using the Monte-Carlo algorithm^27^ was performed to identify the lowest energy conformations of all molecules. Sometimes QSAR models include several low energy conformations for each molecule, however only the lowest energy conformer was used in this study. This is because the experiments are performed at below freezing temperatures where only the lowest energy conformation is highly populated.

Due to the broad structural diversity of the IRI molecules, it was necessary to use alignment independent 3D-descriptors to build the QSAR models. Specifically, GRid INdependent Descriptors (GRIND)^29^ were utilized to correlate the features of the molecular surface curvature and the electrostatical potential (ESP) to IRI activities. Briefly, a regular rectangular grid with 0.176 Å spacing on the surface of each molecule was constructed. By only looking at the distances between nodes and not their orientation within space, GRIND eliminates the need to have all molecules oriented the same way. At each grid point the surface curvature and the electrostatic potential of the molecule was evaluated^30^. Grid points were paired to obtain all combinations of pairs of grid points. These were then sorted based on their distance and placed in bins ranging from 0.33 to 25.83 Å in 0.5 Å increments. Then for each pair of grid points, the product of the two ESP values and two curvature values (auto-correlation), and the cross-correlation of the ESP-curvature was calculated. The maximum product value for the ESP-ESP, curvature-curvature and ESP-curvature at each distance bin was used to create a unique fingerprint for each molecule. Each maximum product represents the unique interaction between nodes at the molecular surface and thus is unique to each specific molecular structure. The electrostatic potential for each molecule was determined at the B3LYP/6-311G(d, p) level of theory using the Gaussian’09 quantum chemistry package^31^. The molecular surface was extracted as an isosurface (0.002 a.u.) of the total electron density from the DFT calculation using an isosurface value empirically shown to correspond to a van der Waals surface^30^. In-house codes were used to evaluate the surface curvature and all molecular fingerprints. A full description of the GRIND descriptors and molecular fingerprints are given in the Supplementary Information.

To build the QSAR models, the 124 molecule data set was divided into a training set of 84 molecules and a test set of the remaining 40 molecules. The molecules were classified as IRI active (A) if the MGS was found to be less than 70%, while those with MGS >70% were classified as inactive (I). The cutoff for activity was chosen to be a MGS of 70%. This is because compounds with IRI activity >70% have been previously described as weakly active to inactive^17^,^32^. Furthermore, this cutoff was found to yield the best correlation between chemical similarity (using the GRIND descriptors) and activity (%MGS). We note that only ~37% of the total set of 124 molecules contains molecules that are IRI active according to this classification. The *training set* was chosen to maintain this distribution of active and inactive molecules. Partial least squares (PLS) regression methods^33^ were used to construct the 3D-QSAR models using the GRIND descriptors previously described. Instead of predicting the absolute IRI activity value, we trained PLSR models with the GRIND descriptors to classify whether an additive has IRI activity lower (active) or higher (inactive) than 70%. In order to reduce the number of descriptors used in the model, a genetic algorithm (GA)^34^ feature selection was utilized, giving an optimized model with only 23 descriptors (reduced from over 150).

## Results and Discussion

The PLS regression yielded an optimum 3D-QSAR model of only 23 GRiND descriptors that successfully identified IRI active compounds in the *training set* with accuracy of 95% given by the area-under the curve (AUC) of the receiver-operator-curve (ROC) plots of the model (see Supplementary Information for details). We consider these results good considering the diversity of molecules in the training set and the fact that only 37% of the molecules in the test set are IRI active. An exhaust leave-one-out (LOO) cross validation was also performed on the *training set* giving comparable results with accuracy of 85%, sensitivity of 82% and precision of 72%. Sensitivity is the number of truly active molecules identified divided by the total number of active molecules in the set and it is a measure of the probability that an active molecule will be identified by the model. On the other hand, precision is the number of truly active molecules out of the total number of molecules identified as such by the model, which relates to the probability that a molecule identify by the model as active is truly active. Therefore, there is always a trade-off between sensitivity and precision that can be measured by their harmonic mean or *F-score* value of binary classification (see Supplementary Information for details). For cross-validation and test set predictions we used the cutoff of maximum F-score, so called harmonic predictions.

Although the QSAR model performed well on the *training set*, these were molecules for which the model parameters and descriptors were optimized to. The important measure of the predictive capability of the QSAR model is the performance on molecules not used in calibration. When applied to the *test set* of 40 molecules that were not part of the *training set*, the 3D-QSAR model successfully recovered 67% of the active molecules with similar precision and higher specificity of ~80% when using the harmonic cutoff for predictions (see Supplementary Information for details). The statistics for the different structural groups in the test set appear in Table 1, where 100% of the active aryl-aldonamides and alkyl-aldonamides were successfully identified in comparison to only 60% of the active aryl-glycosides, whilst precision ranges from ~67% to 75%.

**Figure 4:**
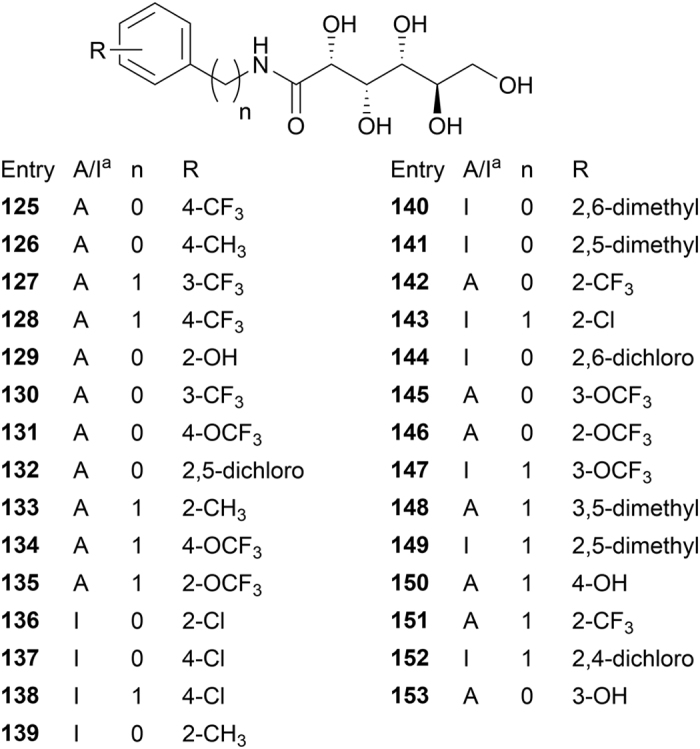
List of 29 proposed aryl-alditol structures. ^a^This column indicates whether the QSAR model predicted the compound to be IRI active (A) or inactive (I).

Encouraged by these results, we chose to use the QSAR model to screen for IRI active molecules that had not yet been synthesized. For this purpose, a small library of 29 aryl-aldonamide structures as shown in Fig. 4 was created and the 3D-QSAR model applied to them. We limited the library to aryl-aldonamides as they were deemed to be most easily synthesized. To prioritize precision over sensitivity, we use a higher cutoff in these predictions than that of maximum *F-score*, which identifies 18 IRI active molecules out of the 29 candidates.

**Figure 5:**
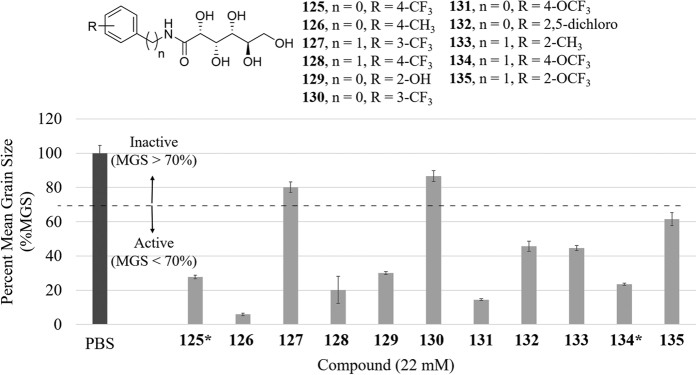
IRI activity of compounds predicted to be active (**125–135**) at 22 mM (except those marked with asterisks which were measured at 11 mM). The dark grey bar represents the PBS control. The dotted line represents the cutoff for activity where a MGS >70% is considered inactive and a MGS <70% is considered active.

11 out of the 18 molecules predicted to be active (**125–135**) were synthesized and tested for IRI activity. The results are summarized in Fig. 5 which shows the experimental IRI activity of these compounds. The line shows the 70% MGS cutoff that was used to classify a compound as active or inactive. Figure 5 shows that 9 out of 11 (82%) of the compounds predicted to be IRI active were determined experimentally to be active. It is worth noticing that this precision value is significantly higher that of 67% for the harmonic predictions of the aryl-aldonamides in Table 1, which provides a strong evidence of the capacity of the model to identify active compounds at a higher cutoff.

The main objective of our predictions is to identify a set of candidates with high IRI activity but the ability of the model to reject inactive compounds was also partially evaluated. In this case, from the 11 compounds predicted to be inactive, six compounds (**136–141**) were synthesized and tested for IRI activity and the experimental activity is shown in the Supplementary Information (Fig. S1). Of the six compounds that were predicted to be IRI inactive, three were experimentally found to be active with a MSG <70%, which represents an inactive omission rate of 50%. However, this is not surprising because in this case the prediction cutoff was selected to enhance the success ratio at the cost of lower sensitivity, therefore some active compounds can be expected among the rejected compounds. However, the fact that precision is more than 30% higher that the inactive omission rate demonstrates the ability of the model to predict molecules that will be experimentally active. This result is a notable achievement that has accelerated the discovery of IRI active small molecules that were previously discovered through a laborious trial-and-error process.

**Figure 6:**
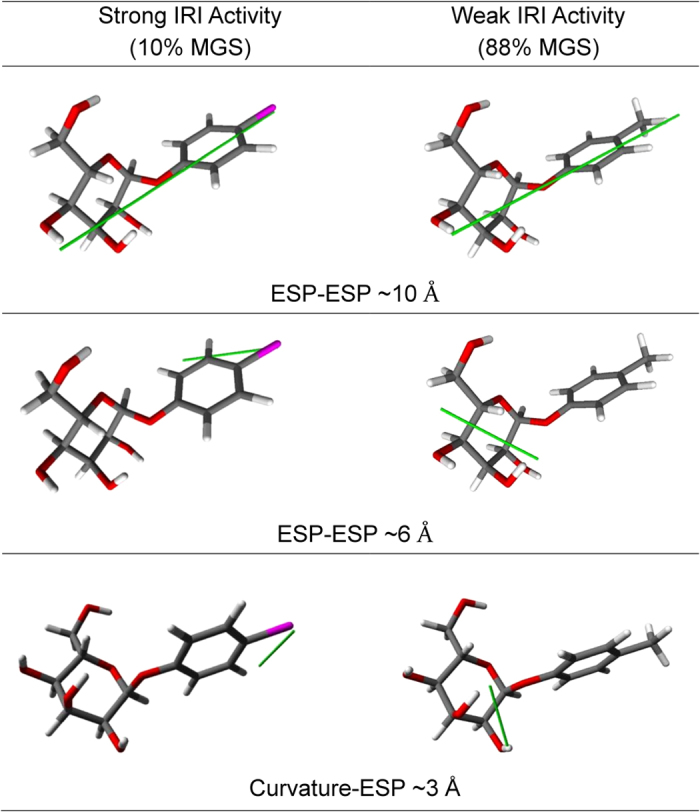
Most relevant features and their specific correlations for compound 6 (left), possessing potent IRI activity and compound 18 (right) which does not possess IRI activity. A prominent ESP-ESP interaction occurs at ~10 Å between the aryl substituent and the farthest hydroxyl group for both active and inactive compounds as shown by the green line. The most prominent ESP-ESP interactions (~6 Å) and curvature-ESP interactions (~3 Å) occur between atoms in the aromatic ring in IRI active compound **6**, whereas these prominent interactions occur within the carbohydrate region in inactive compound **18**.

One challenge with QSAR models is that it can be difficult to extract rational design principles from them. In a limited fashion the GRIND descriptors can highlight potential structural features that influence the activity. The descriptors that were found to have the greatest impact on the IRI activity corresponds to the ESP-ESP interactions at 6.0 and 10.0 Å; and curvature-ESP interactions at 3.0 Å. These features on the structures of a highly active IRI molecule and a related inactive compound are comparatively depicted in Fig. 6. Interestingly, it was found that both active and inactive compounds exhibit prominent ESP-ESP interactions between the substituent in the aryl ring and the hydroxyl group of the sugar ring. Meanwhile, the IRI active compound showed prominent ESP-ESP and curvature-ESP interactions between the aryl group and the substituent of the aryl group, whilst the inactive compound showed the same type of interaction but between the hydroxyl groups of the sugar group. In future studies, these interactions may provide a basis in which to further understand the mechanism of recrystallization inhibition.

## Conclusions

Small molecules capable of inhibiting ice recrystallization represent an exciting class of cryoprotectants. The mechanism by which these molecules inhibit ice recrystallization is not known and this has hampered the rational design of new IRI active compounds resulting in an arduous trial-and-error discovery process. In this work, we have demonstrated that 3D-QSAR modeling can be used to pre-screen compounds for IRI potency to minimize labor intensive chemical synthesis, and ultimately accelerate the discovery process. Using a diverse set of 124 molecules with known IRI activities, a 3D-QSAR model was calibrated that is able to successfully identify IRI active molecules with 80% accuracy in both a training set of 84 molecules and a test set of 40 molecules.

The GRIND descriptors highlighted structural features that had the greatest impact on IRI activity. It was found that a prominent curvature-ESP interaction between the aryl ring atoms at 3 Å occurred in IRI active molecules, while this interaction occurred between carbohydrate atoms in molecules which were inactive. These interactions may provide a basis in which to further understand the mechanism of recrystallization inhibition and this is something that will be explored in future work. Furthermore, it was previously shown that IRI activity is correlated to the hydration of a molecule^17^,^32^. In future studies, renewed building, testing and optimization of the current QSAR model to include hydration parameters are required in order to better understand the role of hydration in the mechanism of ice recrystallization inhibition.

The purpose of this study was to employ QSAR methods to facilitate the design of new small molecules capable of inhibiting ice recrystallization. The QSAR model was used to prescreen a small library of novel IRI active compounds that were then synthesized and tested with a success rate of 82%. This success rate is comparable to that of other QSAR aided drug discoveries. For example, QSAR models were shown to have a 70% success rate for their prediction of blood-brain barrier permeation and central nervous system (CNS) activity^35^. While the model was mainly experimentally validated for the prediction of active molecules, experimental evidence of adequate discrimination of inactive compounds was also provided, which overall has greatly accelerated the discovery of IRI active small molecules that were previously discovered through a laborious trial-and-error process. Thus, QSAR modeling can be used to enrich the sampling of active IRI compounds even though rational design principles for IRI activity do not yet exist.

## Additional Information

**How to cite this article**: Briard, J. G. *et al*. QSAR Accelerated Discovery of Potent Ice Recrystallization Inhibitors. *Sci. Rep.* **6**, 26403; doi: 10.1038/srep26403 (2016).

## Supplementary Material

Supplementary Information

## Figures and Tables

**Figure 1:**
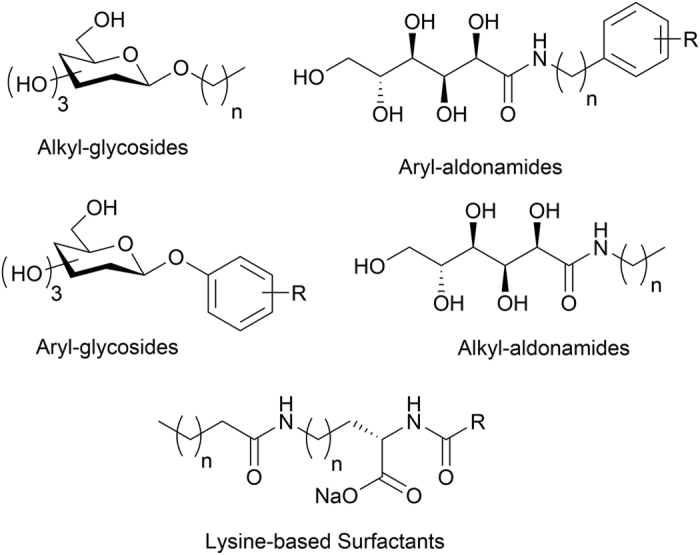
Novel small molecule IRIs.

**Figure 2:**
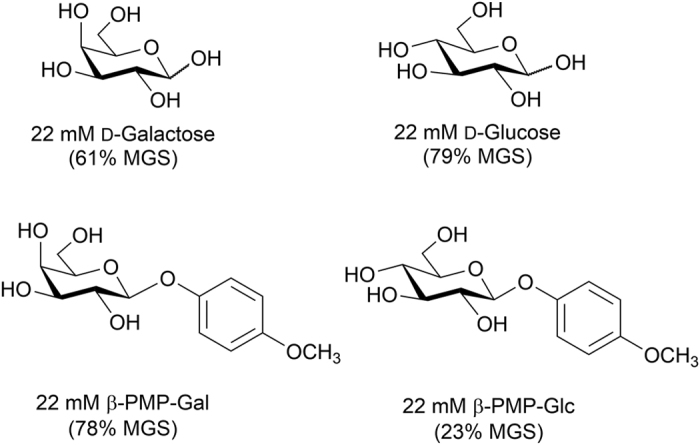
The effects of structural changes on IRI activity. IRI activity values, represented as a percent mean grain size (MGS), are relative to a solution of phosphate buffered saline (positive control for ice recrystallization).

**Table 1:** Prediction statistics of the IRI performance for the different structure groups in the test set at the cutoff of maximum harmonic mean between sensitivity and precision (F-score) (see Supplementary Information for details).

Structures	Sensitivity	Specificity	Number of molecules	Precision
Entire test set	0.67	0.80	40	0.67
Aryl-aldonamides	1.00	0.75	6	0.67
Alkyl-aldonamides	1.00	0.67	6	0.75
Aryl-glycosides	0.60	0.79	24	0.67
