# Supplementary material for: QSAR Accelerated Discovery of Potent Ice Recrystallization Inhibitors
Source: Sci Rep. 2016 May 24;6:26403. doi: 10.1038/srep26403 (PMC4877635; doi:10.1038/srep26403)
Supplement: Supplementary Information [file srep26403-s1.pdf]

Supplementary Information

QSAR Accelerated Discovery of Potent Ice Recrystallization Inhibitors

Jennie G. Briard[1], Michael Fernandez[2], Phil De Luna[2], Robert N. Ben[1]*, and Tom K. Woo[2]*

[1]*Department of Chemistry, University of Ottawa, 10 Marie Curie, Ottawa, Ontario, Canada, K1N 6N5. Fax: 613-562-5170; Tel: 613-562-5800; E-mail: robert.ben@uottawa.ca.*
[2]*Department of Chemistry, University of Ottawa, 10 Marie Curie, Ottawa, Ontario, K1N 6N5. Fax: 613-562-5170; Tel: 613-562-5800; E-mail: twoo@uottawa.ca*

Table of Contents

Inhibition of Ice Recrystallization ……..S3

Splat Cooling Assay……………………………………………………………………………S3

Experimental Section.. …S3

General Experimental…………………………………………………………...…..........S3

Synthesis of Compounds used for the Training and Test Set………………..……….…………S5

Synthesis of Compounds used for the Prediction Set……………………..…….………..…...S53

Computational Details…………………………………………………………………….......S57

Three-Dimensional Structure Generation……………………………………………….S57

Molecular Fingerprint Generation……………………………………………………….S58

Genetic Algorithm Feature Selection………………………………………………........S59

Optimum QSAR Model………………………………………………………..………..S60

References……………………………………………………………………………...………...S61

Inhibition of Ice Recrystallization

Splat Cooling Assay

Sample analysis for IRI activity was performed using the "splat cooling" method as previously described.[1] The analyte was dissolved in phosphate buffered saline (PBS) solution and a 10 μL droplet of this solution was dropped from a height of two meters from a micropipette onto a block of polished aluminum precooled to approximately -80 °C. The droplet froze instantly on the polished aluminum block and was approximately 1 cm in diameter and 20 μm thick. This wafer was then carefully removed from the surface of the block and transferred to a cryostage held at -6.4 °C for annealing. After a period of 30 min, the wafer was photographed using a digital camera (Nikon CoolPix 5000) fitted to the microscope. A total of three images were taken from each wafer. Twelve ice crystal areas were obtained from three images for each wafer for a total of 108 ice crystal areas. Image analysis of the ice wafers was performed using a domain recognition software (DRS)[2] program to determine the mean grain size of ice crystals in the sample. This processing employed the Microsoft Windows Graphical User Interface to allow a user to visually demarcate and store the vertices of ice domains in a digital micrograph. The data was then used to calculate the domain areas. All data was plotted and analyzed using Microsoft Excel. The mean grain (or ice crystal) size (MGS) of the sample was compared to the MGS of the control PBS solution for that same day of testing. IRI activity is reported as the percentage of the MGS (% MGS) relative to the PBS control. Therefore, small percentages represent a small MGS (small ice crystals), which is indicative of high IRI activity. Error bars are reported as the standard error of the mean (SEM).

Experimental Section

General Experimental

All anhydrous reactions were performed in flame-dried glassware under a positive pressure of dry argon. Air or moisture-sensitive reagents and anhydrous solvents were transferred with oven-dried syringes or cannulae. All flash chromatography was performed with E. Merck silica gel 60 (230-400 mesh). All solution phase reactions were monitored using analytical thin layer chromatography (TLC) with 0.2 mm pre-coated silica gel aluminum plates 60 F254 (E. Merck). Components were visualized by illumination with a short-wavelength (254 nm) ultra-violet light and/or staining (ceric ammonium molybdate, potassium permanganate, or phosphomolybdate stain solution). All solvents used for anhydrous reactions were distilled. Tetrahydrofuran (THF) and diethyl ether (Et_2O) were distilled from sodium/benzophenone under nitrogen. Dichloromethane (DCM) was distilled from calcium hydride. N,Ndimethylformamide (DMF) was stored over activated 4Å molecular sieves under argon. ^{1}H (300, 400

or 500 MHz) and ^{13}C NMR (75, 100 or 125 MHz) spectra were recorded at ambient temperature on a Bruker Avance 400, Bruker Avance 500, or Varian Inova 500 spectrometer. Deuterated chloroform ($CDCl_3$), methanol (CD_3OD), or water (D_2O) were used as NMR solvents, unless otherwise stated. Chemical shifts are reported in ppm downfield from trimethylsilane (TMS) or the solvent residual peak as an internal standard. Splitting patterns are designated as follows: s, singlet; d, doublet; t, triplet; q, quartet; quint, quintet; m, multiplet and br, broad. Low resolution mass spectrometry (LRMS) was performed on a Micromass Quatro-LC Electrospray spectrometer with a pump rate of 20 µL/min using electrospray ionization (ESI).

Compounds **118-120** are commercially available and **121-124** were graciously provided by Dr. Mark S. Taylor's laboratory from the Department of Chemistry at the University of Toronto.

Synthesis of Compounds used for the Training and Test Set:

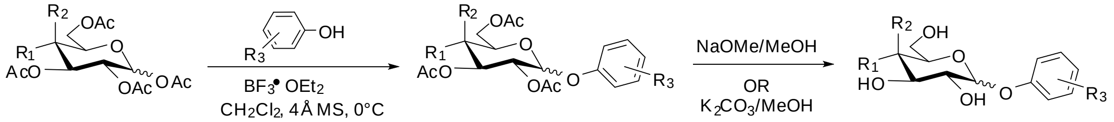

1a: R_1 = H, R_2 = OAc, R_3 = *p*-OCH_3, β-linked
2a: R_1 = OAc, R_2 = H, R_3 = *p*-OCH_3, β-linked
3a: R_1 = H, R_2 = OAc, R_3 = *p*-OCH_3, α-linked
4a: R_1 = OAc, R_2 = H, R_3 = *p*-OCH_3, α-linked
5a: R_1 = OAc, R_2 = H, R_3 = *p*-Br, β-linked
6a: R_1 = OAc, R_2 = H, R_3 = *p*-Cl, β-linked
7a: R_1 = OAc, R_2 = H, R_3 = *p*-F, β-linked
8a: R_1 = OAc, R_2 = H, R_3 = *p*-OH, β-linked
9a: R_1 = OAc, R_2 = H, R_3 = *p*-CH_3, β-linked
10a: R_1 = OAc, R_2 = H, R_3 = H, β-linked
11a: R_1 = OAc, R_2 = H, R_3 = *p*-$COOCH_3$, β-linked
12a: R_1 = OAc, R_2 = H, R_3 = *p*-CF_3, β-linked
13a: R_1 = OAc, R_2 = H, R_3 = *p*-NO_2, β-linked
14a: R_1 = H, R_2 = OAc, R_3 = *p*-Br, β-linked
15a: R_1 = H, R_2 = OAc, R_3 = *p*-Cl, β-linked
16a: R_1 = H, R_2 = OAc, R_3 = *p*-F, β-linked
17a: R_1 = H, R_2 = OAc, R_3 = *p*-OH, β-linked
18a: R_1 = H, R_2 = OAc, R_3 = *p*-CH_3, β-linked
19a: R_1 = H, R_2 = OAc, R_3 = H, β-linked
20a: R_1 = H, R_2 = OAc, R_3 = *p*-$COOCH_3$, β-linked
21a: R_1 = H, R_2 = OAc, R_3 = *p*-NHAc, β-linked
22a: R_1 = H, R_2 = OAc, R_3 = *p*-CF_3, β-linked
23a: R_1 = H, R_2 = OAc, R_3 = *p*-NO_2, β-linked
24a: R_1 = OAc, R_2 = H, R_3 = *m*-CH_3, β-linked
25a: R_1 = OAc, R_2 = H, R_3 = *m*-OCH_3, β-linked
26a: R_1 = OAc, R_2 = H, R_3 = *m*-OH, β-linked
27a: R_1 = H, R_2 = OAc, R_3 = *m*-Br, β-linked
28a: R_1 = H, R_2 = OAc, R_3 = *o*-Br, β-linked
29a: R_1 = H, R_2 = OAc, R_3 = *m*-NHAc, β-linked
30a: R_1 = OAc, R_2 = H, R_3 = *p*-OCF_3, β-linked
31a: R_1 = OAc, R_2 = H, R_3 = H, α-linked
32a: R_1 = OAc, R_2 = H, R_3 = *p*-F, α-linked
33a: R_1 = OAc, R_2 = H, R_3 = *p*-Br, α-linked
34a: R_1 = OAc, R_2 = H, R_3 = *p*-OEt, β-linked
35a: R_1 = OAc, R_2 = H, R_3 = *p*-O-nPr, β-linked
36a: R_1 = OAc, R_2 = H, R_3 = *o*-OCH_3, β-linked
37a: R_1 = H, R_2 = OAc, R_3 = *p*-OCF_3, β-linked
38a: R_1 = H, R_2 = OAc, R_3 = *o*-NHAc, β-linked
39a: R_1 = H, R_2 = OAc, R_3 = *p*-COOEt, β-linked
40a: R_1 = H, R_2 = OAc, R_3 = *p*-COOiPr, β-linked
41a: R_1 = OAc, R_2 = H, R_3 = *p*-COOEt, β-linked
42a: R_1 = OAc, R_2 = H, R_3 = *p*-NHCOnBu, β-linked
43a: R_1 = H, R_2 = OAc, R_3 = *m*-$COOCH_3$, β-linked

1: R_1 = H, R_2 = OH, R_3 = *p*-OCH_3, β-linked
2: R_1 = OH, R_2 = H, R_3 = *p*-OCH_3, β-linked
3: R_1 = H, R_2 = OH, R_3 = *p*-OCH_3, α-linked
4: R_1 = OH, R_2 = H, R_3 = *p*-OCH_3, α-linked
5: R_1 = OH, R_2 = H, R_3 = *p*-Br, β-linked
6: R_1 = OH, R_2 = H, R_3 = *p*-Cl, β-linked
7: R_1 = OH, R_2 = H, R_3 = *p*-F, β-linked
8: R_1 = OH, R_2 = H, R_3 = *p*-OH, β-linked
9: R_1 = OH, R_2 = H, R_3 = *p*-CH_3, β-linked
10: R_1 = OH, R_2 = H, R_3 = H, β-linked
11: R_1 = OH, R_2 = H, R_3 = *p*-$COOCH_3$, β-linked
12: R_1 = OH, R_2 = H, R_3 = *p*-CF_3, β-linked
13: R_1 = OH, R_2 = H, R_3 = *p*-NO_2, β-linked
14: R_1 = H, R_2 = OH, R_3 = *p*-Br, β-linked
15: R_1 = H, R_2 = OH, R_3 = *p*-Cl, β-linked
16: R_1 = H, R_2 = OH, R_3 = *p*-F, β-linked
17: R_1 = H, R_2 = OH, R_3 = *p*-OH, β-linked
18: R_1 = H, R_2 = OH, R_3 = *p*-CH_3, β-linked
19: R_1 = H, R_2 = OH, R_3 = H, β-linked
20: R_1 = H, R_2 = OH, R_3 = *p*-$COOCH_3$, β-linked
21: R_1 = H, R_2 = OH, R_3 = *p*-NHAc, β-linked
22: R_1 = H, R_2 = OH, R_3 = *p*-CF_3, β-linked
23: R_1 = H, R_2 = OH, R_3 = *p*-NO_2, β-linked
24: R_1 = OH, R_2 = H, R_3 = *m*-CH_3, β-linked
25: R_1 = OH, R_2 = H, R_3 = *m*-OCH_3, β-linked
26: R_1 = OH, R_2 = H, R_3 = *m*-OH, β-linked
27: R_1 = H, R_2 = OH, R_3 = *m*-Br, β-linked
28: R_1 = H, R_2 = OH, R_3 = *o*-Br, β-linked
29: R_1 = H, R_2 = OH, R_3 = *m*-NHAc, β-linked
30: R_1 = OH, R_2 = H, R_3 = *p*-OCF_3, β-linked
31: R_1 = OH, R_2 = H, R_3 = H, α-linked
32: R_1 = OH, R_2 = H, R_3 = *p*-F, α-linked
33: R_1 = OH, R_2 = H, R_3 = *p*-Br, α-linked
34: R_1 = OH, R_2 = H, R_3 = *p*-OEt, β-linked
35: R_1 = OH, R_2 = H, R_3 = *p*-O-nPr, β-linked
36: R_1 = OH, R_2 = H, R_3 = *o*-OCH_3, β-linked
37: R_1 = H, R_2 = OH, R_3 = *p*-OCF_3, β-linked
38: R_1 = H, R_2 = OH, R_3 = *o*-NHAc, β-linked
39: R_1 = H, R_2 = OH, R_3 = *p*-COOEt, β-linked
40: R_1 = H, R_2 = OH, R_3 = *p*-COOiPr, β-linked
41: R_1 = OH, R_2 = H, R_3 = *p*-COOEt, β-linked
42: R_1 = OH, R_2 = H, R_3 = *p*-NHCOnBu, β-linked
43: R_1 = H, R_2 = OH, R_3 = *m*-$COOCH_3$, β-linked

4-Methoxyphenyl-2,3,4,6-tetra-O-acetyl-ß-D-galactopyranoside (1a)

To a mixture of 1,2,3,4,6-penta-*O*-acetyl-β-D-galactopyranose (5.0 g, 12.8 mmol), 4-methoxyphenol (2.22 g, 17.9 mmol) and 4 Å MS in anhydrous CH_2Cl_2 (50 mL) stirring at 0 °C under Ar, was slowly added boron trifluoride diethyl etherate (2.09 mL, 16.6 mmol). The reaction mixture was stirred overnight, then diluted with CH_2Cl_2 and quenched with sodium bicarbonate. The solution was filtered through Celite®, then extracted with CH_2Cl_2. The organic layer was washed with sodium bicarbonate, water, saturated brine, then dried over $MgSO_4$ and concentrated. Column chromatography (3:1 hexanes/ethyl acetate)

afforded **1a** as a white powder (5.3 g, 91%). Characterization data is consistent with that previously reported.[3] ^{1}H NMR (500 MHz, $CDCl_3$): δ 6.97-6.94 (m, 2H), 6.84-6.80 (m, 2H), 5.47 - 5.44 (m, 2H),5.09 (dd, *J* = 10.4, 3.4 Hz, 1H), 4.92 (d, *J* = 8.0 Hz, 1H), 4.23 (dd, *J* = 11.3, 6.8 Hz, 1H), 4.16 (dd, *J* = 11.3, 6.5 Hz, 1H), 4.02-3.99 (m, 1H), 3.78 (s, 3H), 2.18 (s, 3H), 2.09 (s, 3H), 2.06 (s, 3H), 2.01 (s, 3H). ^{13}C NMR (100 MHz, $CDCl_3$): δ 170.3, 170.0, 169.4, 169.3, 155.8, 150.9, 118.7, 114.5, 100.7, 72.4, 71.3, 70.2, 66.3, 61.4, 55.5, 20.7, 20.6, 20.6, 20.5. LRMS (ESI): *m/z* calcd. for $C_{21}H_{30}NO_{11}$ $[M+NH_4]^+$ 472.5; found, 472.3.

4-Methoxyphenyl -ß-D-galactopyranoside (1)
Compound **1a** (5.3 g, 11.6 mmol) was dissolved in a solution of sodium methoxide in methanol (25 mL) and stirred for one hour at room temperature. The solution was neutralized with Amberlite® IR-120 (H^+) ion-exchange resin and filtered. The filtrate was concentrated and the product was lyophilized to yield **1** as a white powder (3.3 g, 98%). Characterization data is consistent with that previously reported.[3] ^{1}H NMR (400 MHz, CD_3OD): δ 7.08-7.04 (m, 2H), 6.85-6.81 (m, 2H), 4.72 (d, *J* = 7.7 Hz, 1H), 3.89 (dd, *J* = 3.4, 0.8 Hz, 1H), 3.78-3.74 (m, 3H), 3.74 (s, 3H), 3.63 (ddd, *J* = 6.7, 5.5, 1.1 Hz, 1H), 3.55 (dd, *J* = 9.7, 3.4 Hz, 1H). ^{13}C NMR (101 MHz, D_2O): δ 154.6, 151.0, 118.1, 115.0, 101.7, 75.4, 72.6, 70.6, 68.5, 60.7, 55.8. LRMS (ESI): *m/z* calcd. for $C_{13}H_{18}NaO_7$ $[M+Na]^+$ 309.3; found, 309.3.

4-Methoxyphenyl-2,3,4,6-tetra-O-acetyl-ß-D-glucopyranoside (2a)
To a mixture of 1,2,3,4,6-penta-*O*-acetyl-β-D-glucopyranose (15 g, 38.4 mmol), 4-methoxyphenol (6.7 g, 53.8 mmol) and 4 Å MS in anhydrous CH_2Cl_2 (100 mL) stirring at 0 °C under Ar, was slowly added boron trifluoride diethyl etherate (9.64 mL, 76.8 mmol). The reaction mixture was stirred overnight, then diluted with CH_2Cl_2 and quenched with sodium bicarbonate. The solution was filtered through Celite®, then extracted with CH_2Cl_2. The organic layer was washed with sodium bicarbonate, water, saturated brine, then dried over $MgSO_4$ and concentrated. Column chromatography (3:2 hexanes/ethyl acetate) afforded **2a** as a white powder (13.6 g, 78%). Characterization data is consistent with that previously reported.[4] ^{1}H NMR (300 MHz, $CDCl_3$): δ ^{1}H NMR (300 MHz, $CDCl_3$): δ 6.97-6.92 (m, 2H), 6.84-6.78 (m, 2H), 5.315.20 (m, 2H), 5.16 (t, *J* = 9.6 Hz, 1H), 4.95 (d, *J* = 7.6 Hz, 1H), 4.29 (dd, *J* = 12.3, 5.2 Hz, 1H), 4.18 (dd, *J* = 12.3, 2.5 Hz, 1H), 3.83-3.79 (m, 1H), 3.77 (s, 3H), 2.08 (s, 3H), 2.07 (s, 3H), 2.04 (s, 3H) 2.03 (s, 3H). ^{13}C NMR (75 MHz, $CDCl_3$): δ 170.6, 170.3, 169.4, 169.3, 155.8, 150.9, 118.7, 114.5, 100.3, 72.7, 71.9, 71.2, 68.3, 61.9, 55.6, 20.7, 20.7, 20.6, 20.6. LRMS (ESI): *m/z* calcd. for $C_{21}H_{30}NO_{11}$ $[M+NH_4]^+$ 472.5; found, 472.2; *m/z* calcd. for $C_{21}H_{26}NaO_{11}$ $[M+Na]^+$ 477.4; found, 477.1.

4-Methoxyphenyl -ß-D-glucopyranoside (2)
Compound **2a** (6.75 g, 14.9 mmol) was dissolved in a solution of sodium methoxide in methanol (25 mL) and stirred for one hour at room temperature. The solution was neutralized with Amberlite® IR-120 (H^+) ion-exchange resin and filtered. The filtrate was concentrated and the product was lyophilized to yield **2** as a white powder (4.1 g, 95%). ^{1}H NMR (400 MHz, D_2O): δ 7.14-7.10 (m, 2H), 7.01-6.96 (m, 2H), 5.01 (d, *J* = 7.6 Hz, 1H), 3.92 (dd, *J* = 12.4, 2.2 Hz, 1H), 3.81 (s, 3H), 3.75 (dd, *J* = 12.4, 5.7 Hz, 1H), 3.62-3.54 (m, 3H), 3.48 (dd, *J* = 9.6, 9.2 Hz, 1H). ^{13}C NMR (101 MHz, D_2O): δ 154.7, 150.9, 118.2, 115.0, 101.2, 76.1, 75.5, 72.9, 69.4, 60.5, 55.8. LRMS (ESI): *m/z* calcd. for $C_{13}H_{18}NaO_7$ $[M+Na]^+$ 309.3; found, 309.3.

4-Methoxyphenyl-2,3,4,6-tetra-O-acetyl-α-D-galactopyranoside (3a)
Compound **3a** was prepared in a similar manner as **1a** from 1,2,3,4,6-penta-*O*-acetyl-β-D-galactopyranose (2 g, 5.12 mmol), 4-methoxyphenol (1.91 g, 15.4 mmol) with boron trifluoride diethyl etherate (2.6 mL, 20.5 mmol)**.** The reaction mixture was refluxed for 2 days, and following quenching, washing and concentration the crude mixture showed a 1.6:1 ratio of a:ß-linked products. Column chromatography (4:1 hexanes/EtOAc) afforded **3a** as a white powder (1.4 g, 60%). Characterization data is consistent with that previously reported in the literature.[5] ^{1}H NMR (300 MHz, $CDCl_3$): δ 7.00-6.96 (m, 2H), 6.85-6.81 (m, 2H), 5.65 (d, *J* = 3.7 Hz, 1H), 5.58-5.51 (m, 2H), 5.26 (dd, *J* = 10.4, 3.6 Hz, 1H), 4.39 (t, *J* = 6.7

Hz, 1H), 4.10 (t, J = 6.6 Hz, 2H), 3.77 (s, 3H), 2.16 (s, 3H), 2.08 (s, 3H), 2.02 (s, 3H), 1.97 (s, 3H). ^{13}C NMR (101 MHz, $CDCl_3$): δ 170.5, 170.5, 170.3, 170.2, 155.6, 150.4, 118.2, 114.8, 95.9, 68.1, 68.0, 67.7, 67.1, 61.7, 55.8, 20.9, 20.8, 20.8, 20.8. LRMS (ESI): *m/z* calcd. for $C_{21}H_{30}NO_{11}$ $[M+NH_4]^+$ 472.5; found, 472.0.

4-Methoxyphenyl-α-D-galactopyranoside (3)
Compound **3a** (1.4 g, 3.15 mmol) was dissolved in a solution of sodium methoxide in methanol (15 mL) and stirred for one hour at room temperature. The solution was then neutralized with Amberlite® IR-120 (H^+) ion-exchange resin, filtered and concentrated. The filtrate was concentrated and the product was lyophilized to yield **3** as a white powder (600 mg, 67%). ^{1}H NMR (300 MHz, D_2O): d 7.17-7.12 (m, 2H), 7.01-6.97 (m, 2H), 5.53 (d, J = 3.8 Hz, 1H), 4.12 (t, J = 6.2 Hz, 1H), 4.08-4.03 (m, 2H), 3.99-3.94 (m, 1H), 3.81 (s, 3H), 3.72-3.70 (m, 2H). ^{13}C NMR (76 MHz, D_2O): δ 154.6, 150.4, 119.1, 115.0, 98.5, 71.6, 69.4, 69.1, 68.1, 61.0, 55.7. LRMS (ESI): *m/z* calcd. for $C_{13}H_{17}O_7$ $[M-H]^-$ 285.3; found, 285.1.

4-Methoxyphenyl-2,3,4,6-tetra-O-acetyl-α-D-glucopyranoside (4a)
Compound **4a** was prepared in a similar manner as **2a** from 1,2,3,4,6-penta-*O*-acetyl-β-D-glucopyranose (4 g, 10.3 mmol), 4-methoxyphenol (1.78 g, 14.4 mmol) with boron trifluoride diethyl etherate (2.6 mL, 20.5 mmol)**.** The reaction mixture was refluxed for 2 days, and following quenching, washing and concentration the crude mixture showed a 1:1.6 ratio of α:β-linked products. Column chromatography (4:1 hexanes/EtOAc) afforded **4a** as a white powder (1.43 g, 30%). Characterization data is consistent with that previously reported in the literature.[5] ^{1}H NMR (300 MHz, $CDCl_3$): δ 7.02 - 6.97 (m, 2H), 6.84-6.79 (m, 2H), 5.68 (dd, J = 10.0, 9.7 Hz, 1H), 5.61 (d, J = 3.6 Hz, 1H), 5.13 (dd, J = 10.1, 9.4 Hz, 1H), 5.00 (dd, J = 10.3, 3.7 Hz, 1H), 4.24 (dd, J = 12.1, 4.5 Hz, 1H), 4.15 (ddd, J = 10.2, 4.5, 2.0 Hz, 1H), 4.06 (dd, J = 12.1, 2.2 Hz, 1H), 3.76 (s, 3H), 2.06 (s, 3H), 2.05 (s, 3H), 2.04 (s, 3H), 2.03 (s, 3H). ^{13}C NMR (101 MHz, $CDCl_3$): δ170.6, 170.3, 169.4, 169.3, 158.7, 151.5, 118.7, 115.3, 96.2, 72.2, 71.4, 71.2, 68.3, 61.9, 55.8, 20.7, 20.7, 20.6, 20.6. LRMS (ESI): *m/z* calcd. for $C_{21}H_{30}NO_{11}$ $[M+NH_4]^+$ 472.5; found, 472.3.

4-Methoxyphenyl -α-D-glucopyranoside (4)
Compound **4a** (1.43 g, 3.15 mmol) was dissolved in a solution of sodium methoxide in methanol (15 mL) and stirred for one hour at room temperature. The solution was then neutralized with Amberlite® IR-120 (H^+) ion exchange resin, filtered and concentrated. The filtrate was concentrated and the product was lyophilized to yield **4** as a white powder (780 mg, 87%). ^{1}H NMR (300 MHz, D_2O): δ 7.17-7.12 (m, 2H), 7.02-6.96 (m, 2H), 5.52 (d, J = 3.7 Hz, 1H), 3.90 (t, J = 9.4 Hz, 1H), 3.84-3.76 (m, 3H), 3.81 (s, 3H), 3.70 (dd, J = 9.8, 3.8 Hz, 1H), 3.51 (t, J = 9.5 Hz, 1H). ^{13}C NMR (126 MHz, D_2O): δ 154.5, 150.2, 118.8, 114.9, 98.0, 72.9, 72.3, 71.0, 69.2, 60.1, 55.6. LRMS (ESI): *m/z* calcd. for $C_{13}H_{17}O_7$ $[M-H]^-$ 285.3; found, 285.1.

4-Bromophenyl-2,3,4,6-tetra-O-acetyl-β-D-glucopyranoside (5a)
Compound **5a** was prepared in a similar manner as **2a** from 1,2,3,4,6-penta-*O*-acetyl-β-D-glucopyranose (1.5 g, 3.84 mmol), 4-bromophenol (798 mg, 4.61 mmol) with boron trifluoride diethyl etherate (723 *µ*L, 5.76 mmol). Column chromatography (4:1 hexanes/ethyl acetate) afforded **5a** as a white powder (1.33 g, 69%). Characterization data is consistent with that previously reported.[6] ^{1}H NMR (400 MHz, $CDCl_3$): δ 7.42-7.39 (m, 2H), 6.90-6.86 (m, 2H), 5.31-5.23 (m, 2H), 5.16 (t, J = 9.6 Hz, 1H), 5.03 (d, J = 7.6 Hz, 1H), 4.28 (dd, J = 12.3, 5.4 Hz, 1H), 4.16 (dd, J = 12.3, 2.4 Hz, 1H), 3.84 (ddd, J = 10.0, 5.4, 2.5 Hz, 1H), 2.08 (s, 3H), 2.06 (s, 3H), 2.05 (s, 3H), 2.03 (s, 3H). ^{13}C NMR (101 MHz, $CDCl_3$): δ 170.5, 170.2, 169.4, 155.8, 132.5, 118.8, 115.9, 99.1, 72.6, 72.1, 71.1, 68.2, 61.9, 20.8, 20.8, 20.7, 20.6. LRMS (ESI): *m/z* calcd. for $C_{20}H_{27}BrNO_{10}$ $[M+NH_4]^+$ 520.2; found, 520.0.

4-Bromophenyl-ß-D-glucopyranoside (5)
Compound **5a** (200 mg, 0.40 mmol) was dissolved in a solution of sodium methoxide in methanol (5 mL) and stirred for one hour at room temperature. The solution was then neutralized with Amberlite® IR-120

(H^+) ion-exchange resin, filtered and concentrated. The filtrate was concentrated and the product was lyophilized to yield **5** as a white powder (123 mg, 92%). ^{1}H NMR (400 MHz, D_2O): δ 7.54-7.50 (m, 2H), 7.07-7.03 (m, 2H), 5.09 (d, *J* = 7.5 Hz, 1H), 3.92 (dd, *J* = 12.5, 2.3 Hz, 1H), 3.74 (dd, *J* = 12.5, 5.7 Hz, 1H), 3.64-3.53 (m, 3H), 3.49 (dd, *J* = 9.7, 8.9 Hz, 1H). ^{13}C NMR (101 MHz, D_2O): δ 155.7, 132.6, 118.5, 115.0, 100.2, 76.1, 75.5, 72.9, 69.4, 60.5. LRMS (ESI): *m/z* calcd. for $C_{12}H_{15}BrNaO_6$ $[M+Na]^+$ 357.0; found, 357.0.

4-Chlorophenyl-2,3,4,6-tetra-O-acetyl-β-D-glucopyranoside (6a)

Compound **6a** was prepared in a similar manner as **2a** from 1,2,3,4,6-penta-*O*-acetyl-β-D-glucopyranose (500 mg, 1.28 mmol), 4-chlorophenol (230 mg, 1.79 mmol) with boron trifluoride diethyl etherate (803 *μ*L, 6.4 mmol). Column chromatography (3:2 hexanes/ethyl acetate) afforded **6a** as a white powder (234 mg, 40%). Characterization data is consistent with that previously reported.[7] ^{1}H NMR (400 MHz, $CDCl_3$): δ 7.27-7.23 (m, 4H), 6.95-6.91 (m, 2H), 5.31-5.23 (m, 2H), 5.16 (t, *J* = 9.6 Hz, 1H), 5.03 (d, *J* = 7.6 Hz, 1H), 4.28 (dd, *J* = 12.3, 5.4 Hz, 1H), 4.18-4.15 (m, 1H), 3.84 (ddd, *J* = 9.9, 5.3, 2.4 Hz, 1H), 2.08 (s, 3H), 2.06 (s, 3H), 2.05 (s, 3H), 2.04 (s, 3H). ^{13}C NMR (101 MHz, $CDCl_3$): δ 170.5, 170.2, 169.3, 169.2, 155.3, 129.5, 128.5, 118.4, 99.2, 72.6, 72.1, 71.1, 68.2, 61.9, 20.7, 20.6, 20.6, 20.6. LRMS (ESI): *m/z* calcd. for $C_{20}H_{27}ClNO_{10}$ $[M+NH_4]^+$ 476.9; found, 476.3.

4-Chlorophenyl-β-D-glucopyranoside (6)

Compound **6a** (150 mg, 0.33 mmol) was dissolved in a solution of sodium methoxide in methanol (5 mL) and stirred for one hour at room temperature. The solution was then neutralized with Amberlite® IR-120 (H^+) ion-exchange resin, filtered and concentrated. The filtrate was concentrated and the product was lyophilized to yield **6** as a white powder (92 mg, 97%). ^{1}H NMR (400 MHz, D_2O): δ 7.39-7.35 (m, 2H), 7.127.08 (m, 2H), 5.09 (d, *J* = 7.5 Hz, 1H), 3.92 (dd, *J* = 12.5, 2.2 Hz, 1H), 3.74 (dd, *J* = 12.4, 5.7 Hz, 1H), 3.64-3.53 (m, 3H), 3.49 (t, *J* = 9.3 Hz, 1H). ^{13}C NMR (101 MHz, D_2O): δ 155.2, 129.6, 127.5, 118.0, 100.3, 76.1, 75.5, 72.9, 69.4, 60.5. LRMS (ESI): *m/z* calcd. for $C_{12}H_{15}ClNaO_6$ $[M+Na]^+$ 313.7; found, 313.1.

4-Fluorophenyl-2,3,4,6-tetra-O-acetyl-ß-D-glucopyranoside (7a)

Compound **7a** was prepared in a similar manner as **2a** from 1,2,3,4,6-penta-*O*-acetyl-β-D-glucopyranose (500 mg, 1.28 mmol), 4-fluorophenol (200 mg, 1.58 mmol) with boron trifluoride diethyl etherate (803 *μ*L, 6.4 mmol). Column chromatography (3:2 hexanes/ethyl acetate) afforded **7a** as a white powder (416 mg, 77%). Characterization data is consistent with that previously reported.[8] ^{1}H NMR (400 MHz, $CDCl_3$): δ 7.10-7.08 (m, 2H), 6.91-6.88 (m, 2H), 5.30-5.25 (m, 2H), 5.16 (t, *J* = 9.7 Hz, 1H), 5.03-5.01 (m, 1H), 4.29 (dd, *J* = 12.2, 5.2 Hz, 1H), 4.17 (dd, *J* = 12.2, 2.5 Hz, 1H), 3.83 (ddd, *J* = 10.0, 5.3, 2.5 Hz, 1H), 2.08 (s, 3H), 2.06 (s, 3H), 2.04 (s, 3H), 2.03 (s, 3H). ^{13}C NMR (101 MHz, $CDCl_3$): δ 170.5, 170.2, 169.4, 169.2, 160.0, 157.6, 152.9, 152.9, 118.8, 118.7, 116.1, 115.9, 99.8, 72.6, 72.1, 71.2, 68.2, 61.9, 20.7, 20.6, 20.6, 20.6. LRMS (ESI): *m/z* calcd for $C_{20}H_{27}FNO_{10}$ $[M+NH_4]^+$ 460.5; found, 460.2.

4-Fluorophenyl-ß-D-glucopyranoside (7)

Compound **7a** (198 mg, 0.45 mmol) was dissolved in a solution of sodium methoxide in methanol (5 mL) and stirred for one hour at room temperature. The solution was then neutralized with Amberlite® IR-120 (H^+) ion-exchange resin, filtered and concentrated. The filtrate was concentrated and the product was lyophilized to yield **7** as a white powder (120 mg, 98%). ^{1}H NMR (400 MHz, D_2O): δ 7.15-7.09 (m, 4H), 5.06 (d, *J* = 7.5 Hz, 1H), 3.93 (dd, *J* = 12.4, 2.3 Hz, 1H), 3.75 (dd, *J* = 12.4, 5.6 Hz, 1H), 3.63-3.53 (m, 3H), 3.52-3.47 (m, 1H). ^{13}C NMR (101 MHz, D_2O): δ 152.7, 118.2, 118.2, 116.2, 116.0, 100.9, 76.1, 75.5, 72.9, 69.4, 60.5. LRMS (ESI): *m/z* calcd. for $C_{12}H_{15}FNaO_6$ $[M+Na]^+$ 297.2; found, 297.1.

4-Hydroxyphenyl-2,3,4,6-tetra-O-acetyl-ß-D-glucopyranoside (8a)

Compound **8a** was prepared in a similar manner as **2a** from 1,2,3,4,6-penta-*O*-acetyl-β-D-glucopyranose (1 g, 2.56 mmol), hydroquinone (211 mg, 1.92 mmol) with boron trifluoride diethyl etherate (482 μL, 3.84 mmol). Column chromatography (7:3 hexanes/ethyl acetate) afforded **8a** (339 mg, 40%) as a white powder. Characterization data is consistent with that previously reported.[9] ^{1}H NMR (400 MHz, $CDCl_3$): δ 6.90-6.86 (m, 2H), 6.77-6.72 (m, 2H), 5.30-5.20 (m, 2H), 5.16 (t, *J* = 9.5 Hz, 2H), 4.93 (d, *J* = 7.6 Hz, 1H), 4.28 (dd, *J* = 12.3, 5.1 Hz, 1H), 4.16 (dd, *J* = 12.3, 2.5 Hz, 1H), 3.79 (ddd, *J* = 9.9, 5.1, 2.5 Hz, 1H), 2.09 (d, *J* = 1.5 Hz, 3H), 2.07-2.05 (m, 5H), 2.02 (s, 3H). ^{13}C NMR (101 MHz, $CDCl_3$): δ 170.7, 170.3, 169.5, 169.4, 151.8, 150.8, 118.9, 116.0, 100.3, 72.7, 71.9, 71.2, 68.3, 61.9, 20.7, 20.7, 20.6, 20.6. LRMS (ESI): *m/z* calcd. for $C_{20}H_{24}NaO_{11}$ $[M+Na]^+$ 463.4; found, 463.2.

4-Hydroxyphenyl-ß-D-glucopyranoside (8)

Compound **8a** (200 mg, 0.45 mmol) was dissolved in a solution of sodium methoxide in methanol (5 mL) and stirred for one hour at room temperature. The solution was then neutralized with Amberlite® IR-120 (H^+) ion-exchange resin, filtered and concentrated. The filtrate was concentrated and the product was lyophilized to yield **8** as a white powder (122 mg, 98%). ^{1}H NMR (400 MHz, D_2O): δ 7.07-7.03 (m, 2H), 6.89-6.85 (m, 2H), 4.99 (d, *J* = 7.6 Hz, 1H), 3.92 (dd, *J* = 12.4, 2.2 Hz, 1H), 3.75 (dd, *J* = 12.5, 5.6 Hz, 1H), 3.61-3.52 (m, 3H), 3.48 (dd, *J* = 9.7, 8.9 Hz, 1H). ^{13}C NMR (101 MHz, D_2O): δ 151.2, 150.4, 118.4, 116.2, 101.3, 76.0, 75.5, 73.0, 69.4, 60.5. LRMS (ESI): *m/z* calcd. for $C_{12}H_{16}NaO_7$ $[M+Na]^+$ 295.3; found, 295.2.

4-Methylphenyl-2,3,4,6-tetra-O-acetyl-ß-D-glucopyranoside (9a)

Compound **9a** was prepared in a similar manner as **2a** from 1,2,3,4,6-penta-*O*-acetyl-β-D-glucopyranose (500 mg, 1.28 mmol), 4-methylphenol (190 mg, 1.79 mmol) with boron trifluoride diethyl etherate (800 μL, 6.40 mmol)**.** Column chromatography (7:3 hexanes/EtOAc) afforded **9a** as a white powder (205 mg, 37%). Characterization data is consistent with that previously reported in the literature.[10] ^{1}H NMR (400 MHz, $CDCl_3$): δ 7.09 (d, *J* = 8.2 Hz, 2H), 6.89 (d, *J* = 8.6 Hz, 2H), 5.31-5.23 (m, 2H), 5.16 (t, *J* = 9.6 Hz, 1H), 5.02 (d, *J* = 7.7 Hz, 1H), 4.29 (dd, *J* = 12.2, 5.3 Hz, 1H), 4.16 (dd, *J* = 12.3, 2.3 Hz, 1H), 3.85-3.81 (m, 1H), 2.30 (s, 3H), 2.08 (s, 3H), 2.06 (s, 3H), 2.05 (s, 3H), 2.03 (s, 3H). ^{13}C NMR (101 MHz, $CDCl_3$): δ 170.6, 170.3, 169.4, 169.3, 154.8, 132.9, 130.0, 117.0, 99.6, 72.8, 72.0, 71.2, 68.3, 62.0, 20.7, 20.7, 20.6, 20.6, 20.6. LRMS (ESI): *m/z* calcd. for $C_{21}H_{30}NO_{10}$ $[M+NH_4]^+$ 456.4; found, 456.1.

4-Methylphenyl-ß-D-glucopyranoside (9)

Compound **9a** (124 mg, 0.28 mmol) was dissolved in a solution of sodium methoxide in methanol (5 mL) and stirred for one hour at room temperature. The solution was then neutralized with Amberlite® IR-120 (H^+) ion-exchange resin, filtered and concentrated. The product was purified by recrystallization in hot ethyl acetate to afford **9** as a white powder (68 mg, 89%). Characterization data is consistent with that previously reported in the literature.[10] ^{1}H NMR (400 MHz, D_2O): δ 7.24-7.22 (m, 2H), 7.06-7.04 (m, 2H), 5.08 (d, *J* = 7.6 Hz, 1H), 3.92 (dd, *J* = 12.4, 2.3 Hz, 1H), 3.75 (dd, *J* = 12.4, 5.7 Hz, 1H), 3.63-3.53 (m, 3H), 3.49 (dd, *J* = 10.0, 8.6 Hz, 1H), 2.30 (s, 3H). ^{13}C NMR (101 MHz, D_2O): δ 154.4, 133.3, 130.2, 116.6, 100.5, 76.1, 75.6, 73.0, 69.5, 60.6, 19.6. LRMS (ESI): *m/z* calcd. for $C_{13}H_{18}NaO_6$ $[M+Na]^+$ 293.2; found, 293.1.

Phenyl-2,3,4,6-tetra-O-acetyl-ß-D-glucopyranoside (10a)

Compound **10a** was prepared in a similar manner as **2a** from 1,2,3,4,6-penta-*O*-acetyl-β-D-glucopyranose (500 mg, 1.28 mmol), phenol (169 mg, 1.79 mmol) with boron trifluoride diethyl etherate (209 μL, 1.66 mmol)**.** Column chromatography (7:3 hexanes/EtOAc) afforded **10a** as a white powder (420 mg, 77%). Characterization data is consistent with that previously reported in the literature.[11] ^{1}H NMR (400 MHz, $CDCl_3$): δ 7.30 (dd, *J* = 8.7, 7.4 Hz, 2H), 7.08 (dd, *J* = 7.8, 7.0 Hz, 1H), 6.99 (dt, *J* = 7.8, 1.0 Hz, 2H), 5.33-5.25 (m, 2H), 5.17 (t, *J* = 9.7 Hz, 1H), 5.09 (d, *J* = 7.8 Hz, 1H), 4.29 (dd, *J* = 12.3, 5.4 Hz, 1H), 4.17 (dd, *J* = 12.3, 2.5 Hz, 1H), 3.86 (ddd, *J* = 10.0, 5.3, 2.5 Hz, 1H), 2.09 (s, 3H), 2.06 (s, 3H), 2.05 (s, 3H),

2.04 (s, 3H). ^{13}C NMR (75 MHz, $CDCl_3$): δ 170.6, 170.2, 169.4, 169.3, 156.8, 129.6, 123.4, 117.0, 99.1, 72.7, 72.0, 71.2, 68.3, 61.9, 20.7, 20.6, 20.6. LRMS (ESI): *m/z* calcd. for $C_{20}H_{23}O_{10}$ $[M-H]^-$ 423.4; found, 423.3.

Phenyl-ß-D-glucopyranoside (10)

Compound **10a** (400 mg, 0.94 mmol) was dissolved in a solution of sodium methoxide in methanol (5 mL) and stirred for one hour at room temperature. The solution was then neutralized with Amberlite® IR-120 (H^+) ion-exchange resin, filtered and concentrated. The filtrate was concentrated and the product was lyophilized to yield **10** as a white powder (229 mg, 95%). ^1H NMR (400 MHz, D_2O): δ 7.41-7.37 (m, 2H), 7.16-7.13 (m, 3H), 5.13 (d, *J* = 7.5 Hz, 1H), 3.94-3.90 (m, 1H), 3.74 (dd, *J* = 12.5, 5.7 Hz, 1H), 3.64-3.54 (m, 3H), 3.48 (dd, *J* = 9.8, 8.9 Hz, 1H). ^{13}C NMR (101 MHz, D_2O): δ 156.5, 129.9, 123.3, 116.5, 100.1, 76.1, 75.5, 72.9, 69.4, 60.5. LRMS (ESI): *m/z* calcd. for $C_{12}H_{15}O_6$ $[M-H]^-$ 255.3; found, 255.1.

4-(Methoxycarbonyl)phenyl-2,3,4,6-tetra-O-acetyl-ß-D-glucopyranoside (11a)

Compound **11a** was prepared in a similar manner as **2a** from 1,2,3,4,6-penta-*O*-acetyl-β-D-glucopyranose (500 mg, 1.28 mmol), 4-(methoxycarbonyl)phenol (234 mg, 1.53 mmol) with boron trifluoride diethyl etherate (209 *μ*L, 1.66 mmol)**.** Column chromatography (3:2 hexanes/EtOAc) afforded **11a** as a white powder (258 mg, 77%). Characterization data is consistent with that previously reported in the literature.[4] ^1H NMR (400 MHz, $CDCl_3$): δ 8.02-7.99 (m, 2H), 7.03-6.99 (m, 2H), 5.35-5.28 (m, 2H), 5.20-5.15 (m, 2H), 4.29 (dd, *J* = 12.3, 5.5 Hz, 1H), 4.18 (dd, *J* = 12.3, 2.4 Hz, 1H), 3.92 (m, 1H), 3.90 (s, 3H), 2.08 (d, *J* = 5.8 Hz, 3H), 2.05 (s, 6H), 2.05 (d, *J* = 5.6 Hz, 3H). ^{13}C NMR (101 MHz, $CDCl_3$): δ 170.5, 170.2, 169.4, 169.2, 166.4, 160.1, 131.6, 116.1, 98.2, 72.6, 72.2, 71.0, 68.2, 61.9, 52.0, 20.7, 20.7, 20.6, 20.6. LRMS (ESI): *m/z* calcd. for $C_{22}H_{26}NaO_{12}$ $[M+Na]^+$ 505.4; found, 505.3.

4-(Methoxycarbonyl)phenyl-ß-D-glucopyranoside (11)

Compound **11a** (150 mg, 0.31 mmol) was dissolved in a solution of sodium methoxide in methanol (5 mL) and stirred for one hour at room temperature. The solution was then neutralized with Amberlite® IR-120 (H^+) ion-exchange resin, filtered and concentrated. The filtrate was concentrated and the product was lyophilized to yield **11** as a white powder (87 mg, 89%). ^1H NMR (400 MHz, D_2O): d 8.01 (d, *J* = 8.8 Hz, 2H), 7.18 (d, *J* = 8.9 Hz, 2H), 5.23 (d, *J* = 7.0 Hz, 1H), 3.95-3.92 (m, 1H), 3.90 (s, 3H), 3.75 (dd, *J* = 12.4, 5.6 Hz, 1H), 3.69-3.65 (m, 1H), 3.62-3.58 (m, 2H), 3.50 (t, *J* = 9.3 Hz, 1H). ^{13}C NMR (101 MHz, D_2O): d 169.6, 161.2, 132.4, 124.7, 116.8, 100.1, 76.9, 76.1, 73.5, 70.0, 61.1, 53.2. LRMS (ESI): *m/z* calcd. for $C_{14}H_{18}NaO_8$ $[M+Na]^+$ 337.3; found, 337.2.

4-Trifluoromethylphenyl-2,3,4,6-tetra-O-acetyl-ß-D-glucopyranoside (12a)

Compound **12a** was prepared in a similar manner as **2a** from 1,2,3,4,6-penta-*O*-acetyl-β-D-glucopyranose (500 mg, 1.28 mmol), 4-trifluoromethylphenol (290 mg, 1.79 mmol) with boron trifluoride diethyl etherate (1.6 mL, 12.8 mmol)**.** Column chromatography (3:2 hexanes/EtOAc) afforded **12a** as a white powder (189 mg, 30%). ^1H NMR (400 MHz, $CDCl_3$): δ 7.57 (d, *J* = 8.5 Hz, 2H), 7.07 (d, *J* = 8.4 Hz, 2H), 5.34-5.27 (m, 2H), 5.20-5.14 (m, 2H), 4.29 (dd, *J* = 12.3, 5.4 Hz, 1H), 4.18 (dd, *J* = 12.3, 2.4 Hz, 1H), 3.92-3.87 (m, 1H), 2.07 (s, 3H), 2.06 (s, 3H), 2.06 (s, 3H), 2.04 (s, 3H). ^{13}C NMR (101 MHz, $CDCl_3$): δ 170.5, 170.2, 169.4, 169.2, 127.1, 127.0, 127.0, 127.0, 116.7, 98.4, 72.5, 72.2, 71.0, 68.1, 61.9, 20.7, 20.6, 20.6. LRMS (ESI): *m/z* calcd. for $C_{21}H_{23}F_3NaO_{10}$ $[M+Na]^+$ 515.4; found, 515.2.

4-Trifluoromethylphenyl-ß-D-glucopyranoside (12)

Compound **12a** (117 mg, 0.24 mmol) was dissolved in a solution of sodium methoxide in methanol (5 mL) and stirred for one hour at room temperature. The solution was then neutralized with Amberlite® IR-120 (H^+) ion-exchange resin, filtered and concentrated. The filtrate was concentrated and the product was lyophilized to yield **12** as a white powder (74 mg, 96%). ^1H NMR (400 MHz, D_2O): δ 7.70 (d, *J* = 8.7 Hz, 2H), 7.24 (d, *J* = 8.5 Hz, 2H), 5.21 (d, *J* = 7.6 Hz, 1H), 3.93 (dd, *J* = 12.4, 2.2 Hz, 1H), 3.75 (dd, *J* = 12.4, 5.7 Hz, 1H), 3.69-3.65 (m, 1H), 3.62-3.60 (m, 2H), 3.50 (t, *J* = 9.4 Hz, 1H). ^{13}C NMR (101 MHz, D_2O): δ

159.0, 127.3, 127.2, 127.2, 127.2, 116.5, 99.6, 76.2, 75.5, 72.8, 69.4, 60.5. LRMS (ESI): *m/z* calcd. for $C_{13}H_{15}F_3NaO_6$ $[M+Na]^+$ 347.2; found, 347.1.

4-Nitrophenyl-2,3,4,6-tetra-O-acetyl-ß-D-glucopyranoside (13a)
Compound **13a** was prepared in a similar manner as **2a** from 1,2,3,4,6-penta-*O*-acetyl-β-D-glucopyranose (420 mg, 1.08 mmol), 4-nitrophenol (210 mg, 1.51 mmol) with boron trifluoride diethyl etherate (180 μL, 1.40 mmol)**.** Column chromatography (8:2 hexanes/EtOAc) afforded **13a** as a white powder (347 mg, 68%). Characterization data is consistent with that previously reported in the literature.[11] ^{1}H NMR (500 MHz, $CDCl_3$): δ 8.23-8.20 (m, 2H), 7.09-7.06 (m, 2H), 5.16-5.11 (m, 3H), 5.09 (d, *J* = 3.7 Hz, 1H), 4.28 (dd, *J* = 12.3, 5.2 Hz, 1H), 4.18 (dd, *J* = 12.3, 2.3 Hz, 1H), 3.86-3.82 (m, 1H), 2.07 (s, 3H), 2.06 (s, 3H), 2.03 (s, 3H), 2.02 (s, 3H). ^{13}C NMR (101 MHz, $CDCl_3$): δ 170.7, 170.2, 169.7, 169.3, 160.8, 140.9, 124.7, 115.5, 98.1, 74.7, 73.9, 71.2, 68.1, 61.9, 20.9, 20.7, 20.6, 20.5. LRMS (ESI): *m/z* calcd. for $C_{20}H_{22}NO_{12}$ $[M-H]^-$ 468.4; found, 468.2.

4-Nitrophenyl -ß-D-glucopyranoside (13)
Compound **13a** (250 mg, 0.53 mmol) was dissolved in a solution of sodium methoxide in methanol (4 mL) and stirred for one hour at room temperature. The solution was then neutralized with Amberlite® IR-120 (H^+) ion-exchange resin, filtered and concentrated. The product was purified by column chromatography (4:1:1:1 EtOAc/ACN/H_2O/MeOH) to afford **13** as a white powder (128 mg, 80%). ^{1}H NMR (400 MHz, D_2O): δ 8.28-8.24 (m, 2H), 7.26-7.22 (m, 2H), 5.27 (d, *J* = 7.7 Hz, 1H), 3.93 (dd, *J* = 12.3, 2.1 Hz, 1H), 3.75 (dd, *J* = 12.3, 5.7 Hz, 1H), 3.71-3.67 (m, 1H), 3.63-3.61 (m, 2H), 3.51 (t, *J* = 9.4 Hz, 1H). ^{13}C NMR (101 MHz, D_2O): δ 161.7, 142.6, 126.1, 116.4, 99.4, 76.3, 75.4, 72.7, 69.3, 60.4. LRMS (ESI): *m/z* calcd. for $C_{12}H_{14}NO_8$ $[M-H]^-$ 300.3; found, 300.5. LRMS (ESI): *m/z* calcd. for $C_{24}H_{28}N_2O_{16}$ $[M-H]^-$ Dimer 601.5; found, 601.0.

4-Bromophenyl-2,3,4,6-tetra-O-acetyl-ß-D-galactopyranoside (14a)
Compound **14a** was prepared in a similar manner as **1a** from 1,2,3,4,6-penta-*O*-acetyl-β-D-galactopyranose (500 mg, 1.28 mmol), 4-bromophenol (266 mg, 1.54 mmol) with boron trifluoride diethyl etherate (209 μL, 1.66 mmol). Column chromatography (3:2 hexanes/ethyl acetate) afforded **14a** as a white powder (406 g, 63%). Characterization data is consistent with that previously reported.[6] ^{1}H NMR (400 MHz, $CDCl_3$): δ 7.41-7.37 (m, 2H), 6.90-6.86 (m, 2H), 5.49-5.44 (m, 2H), 5.10 (dd, *J* = 10.4, 3.4 Hz, 1H), 4.99 (d, *J* = 7.9 Hz, 1H), 4.21 (dd, *J* = 11.3, 7.1 Hz, 1H), 4.15 (dd, *J* = 11.3, 6.2 Hz, 1H), 4.06-4.03 (m, 1H), 2.18 (s, 3H), 2.06 (s, 3H), 2.05 (s, 3H), 2.01 (s, 3H). ^{13}C NMR (101 MHz, $CDCl_3$): δ 170.3, 170.2, 170.0, 169.3, 155.9, 132.4, 118.7, 115.8, 99.6, 71.1, 70.7, 68.5, 66.8, 61.3, 20.7, 20.6, 20.6. LRMS (ESI): *m/z* calcd. for $C_{20}H_{27}BrNO_{10}$ $[M+NH_4]^+$ 521.4; found, 522.2.

4-Bromophenyl-ß-D-galactopyranoside (14)
Compound **14a** (300 mg, 0.60 mmol) was dissolved in a solution of sodium methoxide in methanol (5 mL) and stirred for one hour at room temperature. The solution was then neutralized with Amberlite® IR-120 (H^+) ion-exchange resin, filtered and concentrated. The filtrate was concentrated and the product was lyophilized to yield **14** as a white powder (159 mg, 80%). ^{1}H NMR (400 MHz, D_2O): d 7.54-7.50 (m, 2H), 7.08-7.04 (m, 2H), 5.03 (d, *J* = 7.3 Hz, 1H), 3.99 (d, *J* = 2.8 Hz, 1H), 3.88-3.84 (m, 1H), 3.82-3.73 (m, 4H). ^{13}C NMR (101 MHz, D_2O): d 155.9, 132.5, 118.4, 114.8, 100.7, 75.4, 72.5, 70.5, 68.4, 60.7. LRMS (ESI): *m/z* calcd. for $C_{12}H_{15}BrNaO_6$ $[M+Na]^+$ 357.0; found, 357.2.

4-Chlorophenyl-2,3,4,6-tetra-O-acetyl-ß-D-galactopyranoside (15a)
Compound **15a** was prepared in a similar manner as **1a** from 1,2,3,4,6-penta-*O*-acetyl-β-D-galactopyranose (500 mg, 1.28 mmol), 4-chlorophenol (197 mg, 1.54 mmol) with boron trifluoride diethyl etherate (209 μL, 1.66 mmol). Column chromatography (3:2 hexanes/ethyl acetate) afforded **15a** as a white powder (294 mg, 50%). Characterization data is consistent with that previously reported.[7] ^{1}H NMR (400 MHz, $CDCl_3$): δ 7.27-7.23 (m, 2H), 6.96-6.92 (m, 2H), 5.49-5.44 (m, 2H), 5.10 (dd, *J* = 10.4,

3.4 Hz, 1H), 4.99 (d, J = 7.9 Hz, 1H), 4.22 (dd, J = 11.3, 7.0 Hz, 1H), 4.15 (dd, J = 11.3, 6.2 Hz, 1H), 4.06-4.03 (m, 1H), 2.18 (s, 3H), 2.07 (s, 3H), 2.05 (s, 3H), 2.01 (s, 3H). ^{13}C NMR (101 MHz, $CDCl_3$): δ 170.3, 170.2, 170.1, 169.3, 155.4, 129.5, 128.4, 118.3, 99.7, 71.1, 70.7, 68.5, 66.8, 61.3, 20.7, 20.6, 20.6. LRMS (ESI): *m/z* calcd. for $C_{20}H_{27}ClNO_{10}$ $[M+NH_4]^+$ 476.9; found, 476.3.

4-Chlorophenyl-ß-D-galactopyranoside (15)

Compound **15a** (250 mg, 0.55 mmol) was dissolved in a solution of sodium methoxide in methanol (5 mL) and stirred for one hour at room temperature. The solution was then neutralized with Amberlite® IR-120 (H^+) ion-exchange resin, filtered and concentrated. The filtrate was concentrated and the product was lyophilized to yield **15** as a white powder (146 mg, 92%). ^{1}H NMR (400 MHz, D_2O): d 7.39-7.35 (m, 2H), 7.13-7.09 (m, 2H), 5.03 (d, J = 7.2 Hz, 1H), 3.99 (dd, J = 3.1, 0.8 Hz, 1H), 3.86 (t, J = 6.5 Hz, 1H), 3.82-3.74 (m, 4H). ^{13}C NMR (101 MHz, D_2O): d 156.1, 130.2, 128.1, 118.7, 101.5, 76.1, 73.2, 71.2, 69.1, 61.4. LRMS (ESI): *m/z* calcd. for $C_{12}H_{15}ClNaO_6$ $[M+Na]^+$ 313.7; found, 313.2.

4-Fluorophenyl-β-D-galactopyranoside (16)

Compound **16a** was prepared in a similar manner as **1a** from 1,2,3,4,6-penta-*O*-acetyl-β-D-galactopyranose (200 mg, 0.256 mmol), 4-fluorophenol (172 mg, 1.53 mmol) with boron trifluoride diethyl etherate (100 µl, 0.76 mmol). Column chromatography (7:3 hexanes/ethylacetate) afforded **16a** which was then deprotected and recrystallized in a similar manner as **1** to afford **16** as a white crystalline solid (105 mg, 75%). Characterization data is consistent with that previously reported.[12] ^{1}H NMR (500 MHz, D_2O): δ 7.15-7.02 (m, 4H), 4.94 (d, J = 7.6 Hz, 1H), 3.97 (d, J = 3.3 Hz, 1H), 3.87-3.72 (m, 4H), 3.73 (dd, J = 10.0, 3.3 Hz, 1H). ^{13}C NMR (126 MHz, D_2O): δ 158.8 (q, J_{C-F} = 238.0 Hz), 153.2 (d, J_{C-F} = 2.3 Hz), 118.5 (d, J_{C-F} = 8.5 Hz), 116.5 (d, J_{C-F} = 23.4 Hz, C), 101.9, 75.8, 72.9, 71.0, 68.9, 61.1. LRMS (ESI): *m/z* calcd. for $C_{12}H_{15}FO_6$ [M + Na]$^+$ 297.1; found 297.1.

4-Hydroxyphenyl-2,3,4,6-tetra-O-acetyl-ß-D-galactopyranoside (17a)

Compound **17a** was prepared in a similar manner as **1a** from 1,2,3,4,6-penta-*O*-acetyl-β-D-galactopyranose (1 g, 2.56 mmol), hydroquinone (211 mg, 1.92 mmol) with boron trifluoride diethyl etherate (482 μL, 3.84 mmol). Column chromatography (7:3 hexanes/ethyl acetate) afforded both **17a** (185 mg, 22%) as a white powder. Characterization data is consistent with that previously reported.[9] ^{1}H NMR (400 MHz, $CDCl_3$): δ 6.92-6.88 (m, 2H), 6.77-6.73 (m, 2H), 5.47-5.43 (m, 2H), 5.08 (dd, J = 10.5, 3.4 Hz, 1H), 4.90 (d, J = 8.0 Hz, 1H), 4.24 (dd, J = 11.3, 6.9 Hz, 1H), 4.17-4.11 (m, 1H), 4.00 (td, J = 6.7, 1.0 Hz, 1H), 2.18 (s, 3H), 2.09 (s, 3H), 2.05 (s, 3H), 2.01 (s, 3H). ^{13}C NMR (101 MHz, $CDCl_3$): δ 170.4, 170.3, 170.2, 169.5, 151.7, 151.0, 118.8, 116.0, 100.8, 70.9, 70.9, 68.8, 66.9, 61.3, 20.8, 20.7, 20.6. LRMS (ESI): *m/z* calcd. for $C_{20}H_{24}NaO_{11}$ $[M+Na]^+$ 463.4; found, 463.3.

4-Hydroxyphenyl-ß-D-galactopyranoside (17)

Compound **17a** (95 mg, 0.22 mmol) was dissolved in a solution of sodium methoxide in methanol (5 mL) and stirred for one hour at room temperature. The solution was then neutralized with Amberlite® IR-120 (H^+) ion-exchange resin, filtered and concentrated. The filtrate was concentrated and the product was lyophilized to yield **17** as a white powder (41 mg, 70%). ^{1}H NMR (400 MHz, D_2O): δ 7.07-7.02 (m, 2H), 6.88-6.83 (m, 2H), 4.91 (d, J = 7.6 Hz, 1H), 3.97 (d, J = 2.6 Hz, 1H), 3.82-3.71 (m, 5H). ^{13}C NMR (101 MHz, D_2O): δ 151.1, 150.6, 118.3, 116.2, 101.9, 75.3, 72.6, 70.6, 68.5, 60.7. LRMS (ESI): *m/z* calcd. for $C_{12}H_{16}NaO_7$ $[M+Na]^+$ 295.3; found, 295.2.

4-Methylphenyl-β-D-galactopyranoside (18)

Compound **18a** was prepared in a similar manner as **1a** from 1,2,3,4,6-penta-*O*-acetyl-β-D-galactopyranose (1 g, 2.56 mmol), 4-methylphenol (305 mg, 2.82 mmol) with boron trifluoride diethyl etherate (0.46 ml, 3.84 mmol). Column chromatography (7:3 hexanes/ethylacetate) afforded **18a** which was then deprotected and recrystallized in a similar manner as **1** to afford **18** as a yellowish-white crystalline solid (42 mg, 48% over two steps). ^{1}H NMR (500 MHz, D_2O): δ 7.16 (d, J = 8.7 Hz, 2H), 7.00

(d, J = 8.5 Hz, 2H), 4.96 (d, J = 7.2 Hz, 1H), 3.96 (dd, J = 3.1, 0.8 Hz, 1H), 3.82 (ddd, J = 6.4, 5.6, 0.8 Hz, 1H), 3.70 (m, 4H), 2.26 (s, 1H). ^{13}C NMR (126 MHz, D_2O): δ 154.4, 133.0, 130.1, 116.4, 101.0, 75.3, 72.5, 70.49, 70.48, 68.4, 60.7. LRMS (ESI): *m/z* calcd. for $C_{13}H_{18}O_6$ [M + Na]$^+$ 270.1; found 293.1.

Phenyl-β-D-galactopyranoside (19)
Compound **19a** was prepared in a similar manner as **1a** from 1,2,3,4,6-penta-*O*-acetyl-β-D-galactopyranose (1 g, 2.56 mmol), phenol (265 mg, 2.82 mmol) with boron trifluoride diethyl etherate (0.46 ml, 3.84 mmol). Column chromatography (1:1 hexanes/ethylacetate) afforded **19a** which was then deprotected and recrystallized in a similar manner as **1** to afford **19** as a yellowish-white crystalline solid (39 mg, 41% over two steps). Characterization data is consistent with that previously reported.[13] ^{1}H NMR (500 MHz, D_2O): δ 7.40-7.35 (m, 2H), 7.15-7.09 (m, 3H), 5.05 (d, J = 7.4 Hz, 1H), 4.80 (m, 1H), 3.98 (dd, J = 3.2, 0.8 Hz, 1H), 3.85 (ddd, J = 6.6, 5.6, 0.9 Hz, 1H), 3.78 (dd, J = 9.9, 7.4 Hz, 1H), 3.75 (dd, J = 5.3, 3.4 Hz, 1H), 3.74 (dd, J = 10.0, 3.4 Hz, 1H). ^{13}C NMR (126 MHz, D_2O): δ 156.6, 129.8, 123.1, 116.4, 100.6, 75.3, 72.5, 70.5, 68.4, 60.6. LRMS (ESI): *m/z* calcd. for $C_{12}H_{16}O_6$ [M + Na]$^+$ 279.1, found 279.1.

Methyl 4-(2,3,4,6-tetra-O-acetyl-β-D-galactopyranosyloxy)benzoate (20a)
Compound **20a** was prepared in a similar manner as **1a** from 1,2,3,4,6-penta-*O*-acetyl-β-D-galactopyranose (600 mg, 1.54 mmol), methyl-3-hydroxybenzoate[14] (257 mg, 1.69 mmol) with boron trifluoride diethyl etherate (1.90 mL, 15.37 mmol). Column chromatography (4:1 hexanes/EtOAc) afforded **20a** as a white powder (411 mg, 55%). ^{1}H NMR (300 MHz, $CDCl_3$): δ 2.02 (s, 3H), 2.06 (s, 3H), 2.07 (s, 3H), 2.18 (s, 3H), 3.89 (s, 3H), 4.06-4.26 (m, 3H), 5.12 (dd, J = 10.5, 3.4 Hz, 1H), 5.13 (d, J = 7.9 Hz, 1H), 5.47 (dd, J = 3.5, 1.0 Hz, 1H), 5.51 (dd, J = 10.5, 7.9 Hz, 1H), 7.01 (dd, J = 8.9, 2.0 Hz, 2H), 8.00 (dd, J = 8.9, 2.0 Hz, 2H). ^{13}C NMR (75 MHz, $CDCl_3$): δ 20.7, 20.8, 20.8, 20.9, 52.2, 61.5, 66.9, 68.6, 70.9, 71.4, 98.9, 116.3, 125.2, 131.7, 160.4, 166.6, 169.5, 170.2, 170.3, 170.5. LRMS (ESI): *m/z* calcd. for $C_{26}H_{26}KO_{12}$ [M+K]$^+$ 482.1, found 521.2.

Methyl 4-(β-D-galactopyranosyloxy)benzoate (20)
Compound **20a** (409 mg, 0.85 mmol) and potassium carbonate (12 mg, 0.08 mmol) were dissolved in methanol (15 mL) and stirred for two hour at room temperature. The solution was then neutralized with Amberlite® IR-120 (H^+) ion-exchange resin, filtered and concentrated. The filtrate was concentrated and recrystallized in MeOH/CH_2Cl_2 to yield **20** as white crystals (181 mg, 68%). ^{1}H NMR (300 MHz, D_2O): δ 3.74-3.86 (m, 4H), 3.88 (s, 3H), 3.91 (m, 1H), 4.00 (d, J = 3.16 Hz, 1H), 5.15 (d, J = 7.5 Hz, 1H), 7.18 (dd, J = 8.9, 1.8 Hz, 2H), 7.99 (dd, J = 8.9, 1.8 Hz, 2H). ^{13}C NMR (75 MHz, D_2O): δ 52.5, 60.6, 68.4, 70.4, 72.4, 75.5, 99.9, 116.0, 123.8, 131.6, 160.6, 168.9. LRMS (ESI): *m/z* calcd. for $C_{14}H_{18}KO_8$ [M+K]$^+$ 314.3, found 353.1.

4-Acetamidophenyl-2,3,4,6-tetra-O-acetyl-β-D-galactopyranoside (21a)
Compound **21a** was prepared in a similar manner as **1a** from 1,2,3,4,6-penta-*O*-acetyl-β-D-galactopyranose (300 mg, 0.769 mmol), 4-acetamidophenol (128 mg, 0.845 mmol) with boron trifluoride diethyl etherate (1.09 mL, 15.37 mmol). Column chromatography (6:4 hexanes/ethyl acetate) afforded **21a** (148 mg, 40%) as a colourless oil. ^{1}H NMR (300 MHz, $CDCl_3$): δ 1.99 (s, 3H), 2.04 (s, 3H), 2.05 (s, 3H), 2.11 (s, 3H), 2.14 (s, 3H), 4.03 (dd, J = 6.6 Hz, 1H), 4.10-4.18 (m, 2H), 5.05 (d, J = 7.9 Hz, 1H), 5.09 (dd, J = 10.3, 3.4 Hz, 1H), 5.45 (dd, J = 10.2, 7.8 Hz, 1H), 5.42 (m, 1H), 6.91 (d, J = 9.0 Hz, 2H), 7.39 (d, J = 9.0 Hz, 2H), 7.63 (br s, 1H). ^{13}C NMR (75 MHz, $CDCl_3$): δ 20.7, 20.8, 20.8, 20.9, 24.6, 61.5, 67.1, 68.8, 71.0, 71.2, 100.3, 117.8, 121.7, 133.5, 153.7, 168.4, 169.6, 170.3, 170.4, 170.5. LRMS (ESI): *m/z* calcd. for $C_{22}H_{27}NaNO_{11}$ [M+Na]$^+$ 504.2, found 504.3.

4-Acetamidophenyl-β-D-galactopyranoside (21)
Compound **21a** (25 mg, 0.051 mmol) and potassium carbonate (7 mg, 0.0051 mmol) were dissolved in methanol (10 mL) in a 50 mL round bottom flask and stirred overnight at room temperature. Amberlite®

IR-120 resin (H^+) ion-exchange resin was added to the mixture, which was stirred for an additional 10 minutes until a pH of 5 to 6. The mixture was filtered and the solvent was removed under reduced pressure. The crude product was purified by column chromatography (85:15 CH_2Cl_2:MeOH) to yield **21** as white crystals (8 mg, 53%). ^1H NMR (300 MHz, D_2O): δ 2.15 (s, 3H), 3.76-3.81 (m, 4H), 3.86 (dd, *J* = 12.1, 6.1 Hz, 1H), 4.00 (d, *J* = 3.1 Hz, 1H), 5.04 (d, *J* = 7.2 Hz, 1H), 7.14 (d, *J* = 9.0 Hz, 2H), 7.35 (d, *J* = 9.0 Hz, 2H). ^{13}C NMR (75 MHz, D_2O): δ 30.9, 61.4, 69.1, 71.2, 73.2, 76.1, 101.6, 117.7, 124.8, 132.4, 154.9, 173.9. LRMS (ESI): *m/z* calcd. for $C_{12}H_{19}NaNO_7$ $[M+Na]^+$ 336.1, found 336.2.

4-(Trifluoromethyl)phenyl-β-D-galactopyranoside (22)

Compound **22a** was prepared in a similar manner as **1a** from 1,2,3,4,6-penta-*O*-acetyl-β-D-galactopyranose (100 mg, 0.256 mmol), 4-(trifluoromethyl)phenol (46 mg, 0.28 mmol) with boron trifluoride diethyl etherate (50 µl, 0.38 mmol). Column chromatography (6:4 hexanes/ethylacetate) afforded **22a** which was then deprotected and recrystallized in a similar manner as **1** to afford **22** as a white crystalline solid (13 mg, 19% over two steps). ^1H NMR (500 MHz, D_2O): δ 7.75 (d, *J* = 8.4 Hz, 2H), 7.30 (d, *J* = 8.5 Hz, 2H), 5.19 (d, *J* = 7.8 Hz, 1H), 4.06 (d, *J* = 2.7 Hz, 1H), 3.95 (dd, *J* = 6.1, 5.6 Hz, 1H), 3.89 (dd, *J* = 9.0, 8.5 Hz, 1H), 3.85-3.80 (m, 3H). ^{13}C NMR (126 MHz, D_2O): δ 159.1, 127.2, 127.1, 127.1, 116.3, 100.1, 75.4, 72.4, 70.4, 68.3, 60.6. LRMS (ESI): m/z calcd. for $C_{13}H_{15}F_3NaO_6$ $[M + Na]^+$ 347.1, found 347.1.

4-Nitrophenyl-β-D-galactopyranoside (23)

Compound **23a** was prepared in a similar manner as **1a** from 1,2,3,4,6-penta-*O*-acetyl-β-D-galactopyranose (100 mg, 0.256 mmol), 4-nitrophenol (42 mg, 0.28 mmol) with boron trifluoride diethyl etherate (50 µl, 0.38 mmol). Column chromatography (1:1 hexanes/ethylacetate) afforded **23a** which was then deprotected and recrystallized in a similar manner as **1** to afford **23** as a white crystalline solid (15 mg, 19% over two steps). Characterization data is consistent with that previously reported.[15] ^1H NMR (500 MHz, D_2O): δ 8.24 (d, *J* = 9.2 Hz, 2H), 7.22 (d, *J* = 9.3 Hz, 2H), 5.18 (d, *J* = 7.6 Hz, 1H), 3.99 (d, *J* = 3.0 Hz, 1H), 3.91 (dd, *J* = 6.0, 5.9 Hz, 1H), 3.83 (dd, *J* = 9.9, 7.6 Hz, 1H), 3.78-3.74 (m, 3H). ^{13}C NMR (126 MHz, D_2O): δ 161.8, 142.5, 126.1, 116.4, 100.0, 75.6, 72.4, 70.3, 68.4, 60.7. LRMS (ESI): *m/z* calcd. for $C_{12}H_{15}NO_8$ $[M + H]^+$ 302.1, found 302.1.

3-Methylphenyl-2,3,4,6-tetra-O-acetyl-ß-D-glucopyranoside (24a)

Compound **24a** was prepared in a similar manner as **2a** from 1,2,3,4,6-penta-*O*-acetyl-β-D-glucopyranose (500 mg, 1.28 mmol), 3-methylphenol (190 mg, 1.79 mmol) with boron trifluoride diethyl etherate (1.61 mL, 12.8 mmol). Column chromatography (7:3 hexanes/EtOAc) afforded **24a** as a white powder (226 mg, 40%). Characterization data is consistent with that previously reported in the literature.[7] ^1H NMR (400 MHz, $CDCl_3$): δ 7.17 (t, *J* = 7.8 Hz, 1H), 6.90-6.88 (m, 1H), 6.81-6.77 (m, 2H), 5.32-5.24 (m, 2H), 5.16 (t, *J* = 9.6 Hz, 1H), 5.07 (d, *J* = 7.7 Hz, 1H), 4.28 (dd, *J* = 12.3, 5.5 Hz, 1H), 4.17 (dd, *J* = 12.3, 2.4 Hz, 1H), 3.86 (ddd, *J* = 10.0, 5.5, 2.5 Hz, 1H), 2.33 (s, 3H), 2.08 (s, 3H), 2.06 (s, 3H), 2.05 (s, 3H), 2.03 (s, 3H). ^{13}C NMR (101 MHz, $CDCl_3$): δ 170.6, 170.2, 169.4, 169.3, 156.9, 139.7, 129.3, 124.1, 117.7, 113.7, 99.1, 72.7, 72.0, 71.2, 68.4, 62.0, 21.5, 20.7, 20.6, 20.6. LRMS (ESI): *m/z* calcd. for $C_{21}H_{30}NO_{10}$ $[M+NH_4]^+$ 456.5; found, 456.2.

3-Methylphenyl-ß-D-glucopyranoside (24)

Compound **24a** (142 mg, 0.33 mmol) was dissolved in a solution of sodium methoxide in methanol (5 mL) and stirred for one hour at room temperature. The solution was then neutralized with Amberlite® IR-120 (H^+) ion-exchange resin, filtered and concentrated. The filtrate was concentrated and the product was lyophilized to yield **24** as a white powder (79 mg, 89%). ^1H NMR (400 MHz, D_2O): δ 7.29 (t, *J* = 7.9 Hz, 1H), 7.01-6.94 (m, 3H), 5.12 (d, *J* = 7.6 Hz, 1H), 3.93 (dd, *J* = 12.4, 2.2 Hz, 1H), 3.75 (dd, *J* = 12.7, 5.8 Hz, 1H), 3.65-3.54 (m, 3H), 3.49 (dd, *J* = 9.7, 9.1 Hz, 1H), 2.34 (s, 2H). ^{13}C NMR (101 MHz, D_2O): δ 156.6, 140.6, 129.7, 124.0, 117.1, 113.4, 100.1, 76.1, 75.6, 72.9, 69.5, 60.6, 20.5. LRMS (ESI): *m/z* calcd. for $C_{13}H_{18}NaO_6$ $[M+Na]^+$ 293.3; found, 293.1.

3-Methoxyphenyl-2,3,4,6-tetra-O-acetyl-ß-D-glucopyranoside (25a)
Compound **25a** was prepared in a similar manner as **2a** from 1,2,3,4,6-penta-*O*-acetyl-β-D-glucopyranose (500 mg, 1.28 mmol), 3-methoxyphenol (222 mg, 1.79 mmol) with boron trifluoride diethyl etherate (803 *μ*L, 6.4 mmol)**.** Column chromatography (3:2 hexanes/EtOAc) afforded **25a** as a white powder (162 mg, 28%). ^{1}H NMR (400 MHz, $CDCl_3$): δ 7.21-7.17 (m, 1H), 6.64-6.56 (m, 3H), 5.32-5.24 (m, 2H), 5.16 (t, *J* = 9.7 Hz, 1H), 5.08 (d, *J* = 7.7 Hz, 1H), 4.28 (dd, *J* = 12.3, 5.5 Hz, 1H), 4.17 (dd, *J* = 12.2, 2.4 Hz, 1H), 3.86 (ddd, *J* = 10.0, 5.5, 2.5 Hz, 1H), 3.78 (s, 3H), 2.08 (s, 3H), 2.06 (s, 3H), 2.05 (s, 3H), 2.03 (s, 3H). ^{13}C NMR (101 MHz, $CDCl_3$): δ 170.6, 170.2, 169.4, 169.3, 160.8, 158.0, 130.0, 108.9, 108.6, 103.6, 99.0, 72.7, 72.0, 71.1, 68.3, 62.0, 55.4, 30.9, 20.6, 20.6, 20.6. LRMS (ESI): *m/z* calcd. for $C_{21}H_{30}NO_{11}$ $[M+NH_4]^+$ 475.5; found, 472.1.

3-Methoxyphenyl-ß-D-glucopyranoside (25)
Compound **25a** (162 mg, 0.33 mmol) was dissolved in a solution of sodium methoxide in methanol (5 mL) and stirred for one hour at room temperature. The solution was then neutralized with Amberlite® IR-120 (H^+) ion-exchange resin, filtered and concentrated. The filtrate was concentrated and the product was lyophilized to yield **25** as a white powder (90 mg, 95%). ^{1}H NMR (400 MHz, D_2O): δ 7.34-7.30 (m, 1H), 6.78-6.75 (m, 3H), 5.12 (d, *J* = 7.2 Hz, 1H), 3.93 (dd, *J* = 12.1, 1.2 Hz, 1H), 3.82 (s, 3H), 3.76-3.72 (m, 1H), 3.65-3.54 (m, 3H), 3.51-3.46 (m, 1H). ^{13}C NMR (101 MHz, D_2O): δ 160.1, 157.7, 130.6, 109.0, 108.9, 102.9, 100.1, 76.2, 75.5, 72.9, 69.5, 60.6, 55.5. LRMS (ESI): *m/z* calcd. for $C_{13}H_{22}NO_7$ $[M+NH_4]^+$ 304.4; found, 304.1.

3-Hydroxyphenyl-2,3,4,6-tetra-O-acetyl-ß-D-glucopyranoside (26a)
Compound **26a** was prepared in a similar manner as **2a** from 1,2,3,4,6-penta-*O*-acetyl-β-D-glucopyranose (1 g, 2.56 mmol), resorcinol (170 mg, 1.54 mmol) with boron trifluoride diethyl etherate (804 *μ*L, 6.4 mmol). Column chromatography (3:2 hexanes/ethyl acetate) afforded **26a** (221 mg, 33%) as a white powder. ^{1}H NMR (400 MHz, $CDCl_3$): δ 7.10 (t, *J* = 8.2 Hz, 1H), 6.55-6.51 (m, 3H), 5.25 (qd, *J* = 11.2, 9.4 Hz, 2H), 5.14 (t, *J* = 9.4 Hz, 1H), 5.05 (d, *J* = 7.5 Hz, 1H), 4.27 (dd, *J* = 12.3, 5.3 Hz, 1H), 4.16 (dd, *J* = 12.3, 2.3 Hz, 1H), 3.84 (ddd, *J* = 9.9, 5.2, 2.4 Hz, 1H), 2.07 (s, 3H), 2.04 (s, 3H), 2.03 (s, 3H), 2.02 (s, 3H). ^{13}C NMR (101 MHz, $CDCl_3$): δ 171.0, 170.4, 169.5, 157.9, 157.1, 130.1, 110.4, 108.7, 104.6, 98.9, 72.7, 71.9, 71.2, 68.3, 61.9, 20.6, 20.6, 20.52. LRMS (ESI): *m/z* calcd. for $C_{20}H_{24}NaO_{11}$ $[M+Na]^+$ 463.4; found, 463.1.

3-Hydroxyphenyl-ß-D-glucopyranoside (26)
Compound **26a** (88 mg, 0.20 mmol) was dissolved in a solution of sodium methoxide in methanol (5 mL) and stirred for one hour at room temperature. The solution was then neutralized with Amberlite® IR-120 (H^+) ion-exchange resin, filtered and concentrated. The filtrate was concentrated and the product was lyophilized to yield **26** as a white powder (54 mg, 98%). ^{1}H NMR (400 MHz, D_2O): δ 7.25 (t, *J* = 8.5 Hz, 1H), 6.70 (ddd, *J* = 8.3, 2.2, 0.9 Hz, 1H), 6.65 (dquintet, *J* = 4.8, 1.6 Hz, 2H), 5.10 (d, *J* = 7.6 Hz, 1H), 3.93 (dd, *J* = 12.4, 2.2 Hz, 1H), 3.75 (dd, *J* = 12.4, 5.7 Hz, 1H), 3.65-3.52 (m, 3H), 3.48 (dd, *J* = 9.7, 8.9 Hz, 1H). ^{13}C NMR (101 MHz, D_2O): δ 158.5, 157.6, 131.4, 111.0, 109.0, 104.7, 100.7, 76.8, 76.2, 73.6, 70.1, 61.2. LRMS (ESI): *m/z* calcd. for $C_{12}H_{16}NaO_7$ $[M+Na]^+$ 295.2; found, 295.1.

3-Bromophenyl-2,3,4,6-tetra-O-acetyl-β-D-galactopyranoside (27a)
Compound **27a** was prepared in a similar manner as **1a** from 1,2,3,4,6-penta-*O*-acetyl-β-D-galactopyranose (200 mg, 0.51 mmol), 3-bromophenol (98 mg, 0.56 mmol) with boron trifluoride diethyl etherate (1.58 mL, 12.81 mmol). Column chromatography (3:2 hexanes/ethyl acetate) afforded **27a** (53 mg, 59%) as a yellow oil. ^{1}H NMR (300 MHz, $CDCl_3$): δ 2.01 (s, 3H), 2.07 (s, 3H), 2.09 (s, 3H), 2.18 (s, 3H), 4.04-4.24 (m, 3H), 5.02 (d, *J* = 7.9 Hz, 1H), 5.10 (dd, *J* = 10.4, 3.4 Hz, 1H), 5.46 (dd, *J* = 3.5, 1.0 Hz, 1H), 5.48 (dd, *J* = 10.4, 7.9 Hz, 1H), 6.93 (ddd, *J* = 7.9, 2.4, 1.3 Hz, 1H), 7.16 (dd, *J* = 7.9, 7.9 Hz, 1H), 7.19-7.24 (m, 2H). ^{13}C NMR (75 MHz, $CDCl_3$): δ 20.6, 20.7, 20.7, 20.7, 61.7, 67.0, 68.5, 70.8, 71.3,

99.4, 116.0, 120.1, 122.7, 126.4, 130.7, 157.5, 169.4, 170.1, 170.2, 170.5. LRMS (ESI): *m/z* calcd. for $C_{20}H_{23}BrKO_{10}$ $[M+K]^+$ 542.4, found 542.7.

3-Bromophenyl-β-D-galactopyranoside (27)
Compound **27a** (138 mg, 0.275 mmol) was dissolved in a solution of sodium methoxide in methanol (5 mL) and stirred for one hour at room temperature. The solution was then neutralized with Amberlite® IR-120 (H^+) ion-exchange resin, filtered and concentrated. The filtrate was concentrated and the product was lyophilized to yield **27** as a white powder (60 mg, 65%). ^{1}H NMR (300 MHz, D_2O): δ 3.70-3.85 (m, 5H), 3.98 (d, *J* = 3.1 Hz, 1H), 5.00 (d, *J* = 7.3 Hz, 1H), 7.08 (m, 1H), 7.25 (dd, *J* = 8.9, 8.9 Hz, 1H), 7.27-7.32 (m, 2H). ^{13}C NMR (75 MHz, D_2O): δ 60.6, 68.3, 70.4, 72.5, 75.4, 100.6, 115.4, 119.6, 122.1, 126.0, 131.1, 157.3. LRMS (ESI): *m/z* calcd. for $C_{12}H_{15}BrKO_6$ $[M+K]^+$ 374.3, found 373.0.

2-Bromophenyl-2,3,4,6-tetra-O-acetyl-β-D-galactopyranoside (28a)
Compound **28a** was prepared in a similar manner as **1a** from 1,2,3,4,6-penta-*O*-acetyl-β-D-galactopyranose (500 mg, 1.28 mmol), 2-bromophenol (443 mg, 2.56 mmol) with boron trifluoride diethyl etherate (1.58 mL, 12.81 mmol). Column chromatography (3:2 hexanes/ethyl acetate) afforded **28a** (341 mg, 53%) as a yellow oil. ^{1}H NMR (300 MHz, $CDCl_3$): δ 7.53 (dd, *J* = 8.0, 1.6 Hz, 1H), 7.25 (ddd, *J* = 8.8, 7.3, 1.6 Hz, 1H), 7.16 (dd, *J* = 8.3, 1.6Hz, 1H), 6.96 (ddd, *J* = 7.9, 7.3, 1.6 Hz, 1H), 5.59 (dd, *J* = 10.6, 8.0 Hz, 1H), 5.46 (dd, *J* = 3.4, 1.0 Hz, 1H), 5.10 (dd, *J* = 10.5, 3.4 Hz, 1H), 4.48 (d, *J* = 8.0 Hz, 1H), 4.29-4.02 (m, 3H), 2.18 (s, 3H), 2.10 (s, 3H), 2.06 (s, 3H), 2.01 (s, 3H). ^{13}C NMR (75 MHz, $CDCl_3$): δ 170.4, 170.3, 170.2, 169.4, 153.7, 133.6, 128.6, 124.7, 117.8, 133.2, 100.6, 71.2, 70.8, 68.2, 66.9, 61.4, 21.0, 20.7, 20.6. LRMS (ESI): *m/z* calcd. for $C_{20}H_{23}BrKO_{10}$ $[M+K]^+$ 542.4, found 542.2.

2-Bromophenyl-β-D-galactopyranoside (28)
Compound **28a** (320 mg, 0.636 mmol) and potassium carbonate (9 mg, 0.0636 mmol) were dissolved in methanol (5 mL) in a 50 mL round bottom flask and stirred for two hours at room temperature. Amberlite® IR-120 (H^+) ion exchange resin was added to the mixture, which was stirred for an additional 10 minutes until a pH of 5 to 6. The mixture was filtered and the solvent was removed under reduced pressure. The product purified by dissolving in methanol and precipitating with diethyl ether to yield **28** as white crystals (126 mg, 59%). ^{1}H NMR (500 MHz, MeOD): δ 7.53 (dd, *J* = 7.8, 1.2 Hz, 1H), 7.31-7.25 (m, 2H), 6.91 (ddd, 1H, *J* = 8.2, 6.2, 1.8 Hz), 4.95 (d, *J* = 7.8 Hz, 1H), 3.91 (d, *J* = 3.3 Hz, 1H), 3.88 (dd, *J* = 9.7, 7.7 Hz, 1H), 3.79-3.74 (m, 2H), 3.71-3.68 (m, 1H), 3.59 (dd, *J* = 9.7, 3.5Hz, 1H). ^{13}C NMR (125 MHz, MeOD): δ 155.5, 134.3, 129.7, 124.4, 117.7, 113.4, 102.9, 77.2, 75.0, 72.2, 70.2, 62.4. LRMS (ESI): *m/z* calcd. for $C_{12}H_{15}BrKO_6$ $[M+K]^+$ 374.3, found 373.3.

3-Acetamidophenyl-2,3,4,6-tetra-O-acetyl-ß-D-galactopyranoside (29a)
Compound **29a** was prepared in a similar manner as **1a** from 1,2,3,4,6-penta-*O*-acetyl-β-D-galactopyranose (500 mg, 1.28 mmol), 3-acetamidophenol (213 mg, 1.41 mmol) with boron trifluoride diethyl etherate (1.58 mL, 12.81 mmol). Column chromatography (8:2 hexanes/ethyl acetate) afforded **29a** (136 mg, 22%) as a yellow oil. ^{1}H NMR (300 MHz, $CDCl_3$): δ 7.40 (dd, *J* = 1.7, 1.6 Hz, 1H), 7.19 (dd, *J* = 8.2, 8.2 Hz, 1H), 7.10 (dd, *J* = 8.1, 1.6 Hz, 1H), 6.73 (dd, *J* = 8.2, 1.7 Hz, 1H), 5.45 (dd, *J* = 10.4, 7.9 Hz, 1H), 5.43 (dd, *J* = 3.5, 1.0 Hz, 1H), 5.08 (dd, *J* = 10.4, 3.5 Hz, 1H), 5.05 (d, *J* = 8.0 Hz, 1H), 4.22-4.06 (m, 3H), 2.15 (s, 3H), 2.14 (s, 3H), 2.06 (s, 3H), 2.03 (s, 3H), 1.99 (s, 3H). ^{13}C NMR (75 MHz, $CDCl_3$): δ 170.6, 170.4, 170.2, 169.6, 168.6, 157.4, 139.5, 129.9, 114.5, 112.8, 108.7, 99.6, 71.1, 70.9, 68.4, 67.0, 61.4, 24.6, 20.8, 20.7, 20.7. LRMS (ESI): *m/z* calcd. for $C_{22}H_{27}NaNO_{11}$ $[M+Na]^+$ 504.5, found 504.3.

3-Acetamidophenyl ß-D-galactopyranoside (29)
Compound **29a** (136 mg, 0.28 mmol) and potassium carbonate (4 mg, 0.028 mmol) were dissolved in methanol (5 mL) in a 50 mL round bottom flask and stirred for 24 hours at room temperature. Amberlite® IR-120 (H^+) ion exchange resin was added to the mixture, which was stirred for an additional

10 minutes until a pH of 5 to 6. The mixture was filtered and the solvent was removed under reduced pressure to yield **29** as white crystals (70 mg, 79%). ^{1}H NMR (300 MHz, D_2O): δ 7.34 (dd, *J* = 8.2, 8.2 Hz, 1H), 7.31 (dd, *J* = 2.4, 2.3 Hz, 1H), 7.13 (ddd, *J* = 8.1, 1.9, 0.8 Hz, 1H), 6.94 (ddd, *J* = 8.2, 2.4, 0.7 Hz, 1H), 5.02 (d, *J* = 7.2 Hz, 1H), 4.02 (dd, *J* = 3.1, 0.7 Hz, 1H), 3.89-3.77 (m, 3H), 2.16 (s, 3H). ^{13}C NMR (75 MHz, D_2O): δ 170.6, 170.4, 170.2, 169.6, 168.6, 157.4, 139.5, 129.9, 114.5, 112.8, 108.7, 99.6, 71.1, 70.9, 68.7, 67.0, 61.4, 24.6, 20.8, 20.7, 20.7. LRMS (ESI): *m/z* calc. for $C_{16}H_{25}N_3O_7$ $[M+CH_3CN-NH_3]^+$ 372.1, found 372.3.

4-Trifluoromethoxyphenyl-2,3,4,6-tetra-O-acetyl-ß-D-glucopyranoside (30a)

Compound **30a** was prepared in a similar manner as **2a** from 1,2,3,4,6-penta-*O*-acetyl-β-D-glucopyranose (250 mg, 0.64 mmol), 4-trifluoromethoxyphenol (116 *µ*L, 0.90 mmol) with boron trifluoride diethyl etherate (161 *µ*L, 1.28 mmol)**.** Column chromatography (4:1 hexanes/EtOAc) afforded **30a** as a white powder (291 mg, 89%). ^{1}H NMR (300 MHz, $CDCl_3$): δ 7.15 (d, *J* = 8.9 Hz, 2H), 7.00 (d, *J* = 9.0 Hz, 2H), 5.33-5.23 (m, 2H), 5.16 (t, *J* = 9.5 Hz, 1H), 5.06 (d, *J* = 7.3 Hz, 1H), 4.28 (dd, *J* = 12.3, 5.3 Hz, 1H), 4.16 (dd, *J* = 12.3, 2.3 Hz, 1H), 3.88-3.82 (m, 1H), 2.07 (s, 6H), 2.05 (s, 3H), 2.03 (s, 3H). ^{13}C NMR (76 MHz, $CDCl_3$): δ 170.5, 170.2, 169.4, 169.2, 155.1, 144.6, 122.5, 118.1, 99.1, 72.6, 72.1, 71.1, 68.1, 61.8, 20.6, 20.6, 20.6. LRMS (ESI): *m/z* calcd. for $C_{21}H_{22}F_3NaO_{11}$ $[M-H]^-$ 507.4; found, 507.2.

4-Trifluoromethoxyphenyl-ß-D-glucopyranoside (30)

Compound **30a** (180 mg, 0.35 mmol) was dissolved in a solution of sodium methoxide in methanol (5 mL) and stirred for one hour at room temperature. The solution was then neutralized with Amberlite® IR-120 (H^+) ion-exchange resin, filtered and concentrated. The filtrate was concentrated and the product was lyophilized to yield **30** as a white powder (110 mg, 91%). ^{1}H NMR (500 MHz, D_2O): δ 7.30 (d, *J* = 9.1 Hz, 2H), 7.17-7.15 (m, 2H), 5.09 (d, *J* = 7.3 Hz, 1H), 3.91 (dd, *J* = 12.5, 2.1 Hz, 1H), 3.73 (t, *J* = 9.0 Hz, 1H), 3.62-3.55 (m, 3H), 3.48 (d, *J* = 9.2 Hz, 1H). ^{13}C NMR (126 MHz, D_2O): δ 155.0, 144.1, 122.6, 119.2, 117.6, 100.3, 76.1, 75.4, 72.8, 69.3, 60.4. LRMS (ESI): *m/z* calcd. for $C_{13}H_{14}F_3NaO_7$ $[M-H]^-$ 339.2; found, 339.1.

Phenyl-2,3,4,6-tetra-O-acetyl-a-D-glucopyranoside (31a)

Compound **31a** was prepared in a similar manner as **2a** from 1,2,3,4,6-penta-*O*-acetyl-β-D-glucopyranose **(**7.5 g, 19.2 mmol), phenol (2.2 g, 23.1 mmol) with boron trifluoride diethyl etherate (4.8 mL, 38.4 mmol). The reaction mixture was refluxed for 2 days, and following quenching, washing and concentration the crude mixture showed a 1:13 ratio of α:β-linked products. Column chromatography (4:1 hexanes/EtOAc) afforded **31a** as a white powder (486 mg, 6%). ^{1}H NMR (300 MHz, $CDCl_3$): δ 7.31 (dd, *J* = 8.9, 7.2 Hz, 2H), 7.10-7.04 (m, 3H), 5.75-5.68 (m, 2H), 5.16 (dd, *J* = 10.2, 9.4 Hz, 1H), 5.04 (dd, *J* = 10.2, 3.7 Hz, 1H), 4.25 (dd, *J* = 12.1, 4.5 Hz, 1H), 4.16-4.11 (m, 1H), 4.06 (dd, *J* = 12.2, 2.2 Hz, 1H), 2.06 (s, 3H), 2.06 (s, 3H), 2.04 (s, 3H), 2.04 (s, 3H). ^{13}C NMR (76 MHz, $CDCl_3$): δ 170.5, 170.1, 169.6, 156.0, 129.6, 123.0, 116.5, 94.2, 70.4, 70.1, 68.3, 67.9, 61.6, 20.7, 20.6, 20.6. LRMS (ESI): *m/z* calcd. for $C_{20}H_{23}O_{10}$ $[M-H]^-$ 423.4; found, 423.3.

Phenyl-a-D-glucopyranoside (31)

Compound **31a** (26 mg, 0.06 mmol) was dissolved in a solution of sodium methoxide in methanol (2 mL) and stirred for one hour at room temperature. The solution was then neutralized with Amberlite® IR-120 (H+) ion exchange resin, filtered and concentrated. The filtrate was concentrated and the product was lyophilized to yield **31** as a white powder (12 mg, 78%). ^{1}H NMR (300 MHz, D_2O): δ 7.41 (dd, *J* = 8.8, 7.3 Hz, 2H), 7.21-7.12 (m, 3H), 5.65 (d, *J* = 3.7 Hz, 1H), 3.94 (t, *J* = 9.5 Hz, 1H), 3.81-3.71 (m, 4H), 3.52 (t, *J* = 9.3 Hz, 1H). ^{13}C NMR (76 MHz, D_2O): δ 155.9, 129.9, 123.1, 117.2, 97.0, 73.0, 72.4, 71.1, 69.3, 60.2. LRMS (ESI): *m/z* calcd. for $C_{12}H_{15}O_6$ $[M-H]^-$ 255.3; found, 255.2.

4-Fluorophenyl-2,3,4,6-tetra-O-acetyl-a-D-glucopyranoside (32a)
Compound **32a** was prepared in a similar manner as **2a** from 1,2,3,4,6-penta-*O*-acetyl-β-D-glucopyranose (10 mg, 25.6 mmol), 4-fluorophenol (3.45 g, 30.8 mmol) with boron trifluoride diethyl etherate (4.5 mL, 35.8 mmol). The reaction mixture was refluxed for 2 days, and following quenching, washing and concentration the crude mixture showed a 1:4 ratio of α:β-linked products. Column chromatography (4:1 hexanes/EtOAc) afforded **32a** as a white powder (1.02 g, 9%). ^{1}H NMR (300 MHz, $CDCl_3$): δ 7.03-6.99 (m, 4H), 5.71-5.64 (m, 2H), 5.14 (t, *J* = 9.7 Hz, 1H), 5.02 (dd, *J* = 10.3, 3.7 Hz, 1H), 4.24 (dd, *J* = 11.9, 4.4 Hz, 1H), 4.15-4.04 (m, 2H), 2.07 (s, 3H), 2.05 (s, 3H), 2.04 (s, 3H), 2.04 (s, 3H). ^{13}C NMR (76 MHz, $CDCl_3$): δ 170.5, 170.1, 169.6, 160.2, 157.0, 152.2, 152.1, 118.0, 117.9, 116.3, 116.0, 94.8, 70.4, 69.9, 68.3, 68.0, 61.6, 20.7, 20.6, 20.6. LRMS (ESI): *m/z* calcd. for $C_{20}H_{27}FNO_{10}$ $[M+NH_4]^+$ 460.5; found, 460.4.

4-Fluorophenyl-a-D-glucopyranoside (32)
Compound **32a** (15 mg, 0.033 mmol) was dissolved in a solution of sodium methoxide in methanol (2 mL) and stirred for one hour at room temperature. The solution was then neutralized with Amberlite® IR-120 (H^+) ion-exchange resin, filtered and concentrated. The filtrate was concentrated and the product was lyophilized to yield **32** as a white powder (6 mg, 66%). ^{1}H NMR (300 MHz, D_2O): δ 7.187.07 (m, 4H), 5.55 (d, *J* = 3.7 Hz, 1H), 3.90 (t, *J* = 9.5 Hz, 1H),3.82-3.69 (m, 5H), 3.50 (t, *J* = 9.4 Hz, 1H). ^{13}C NMR (76 MHz, D_2O): δ 148.9, 118.2, 118.2, 116.2, 114.4, 97.1, 75.5, 72.9, 72.3, 69.4, 60.5. LRMS (ESI): *m/z* calcd. for $C_{12}H_{15}FNaO_6$ $[M+Na]^+$ 297.2; found, 297.3.

4-Bromophenyl-2,3,4,6-tetra-O-acetyl-a-D-glucopyranoside (33a)
Compound **33a** was prepared in a similar manner as **2a** from 1,2,3,4,6-penta-*O*-acetyl-β-D-glucopyranose (8 g, 20.5 mmol), 4-bromophenol (4.25 mg, 24.6 mmol) with boron trifluoride diethyl etherate (3.86 mL, 30.8 mmol)**.** The reaction mixture was refluxed for 2 days, and following quenching, washing and concentration the crude mixture showed a 1:6 ratio of α:β-linked products. Column chromatography (4:1 hexanes/EtOAc) afforded **33a** as a white powder (1.14 g, 11%). Characterization data is consistent with that previously reported in the literature.[16] ^{1}H NMR (300 MHz, $CDCl_3$): δ 7.39 (d, *J* = 9.0 Hz, 2H), 6.96 (d, *J* = 9.0 Hz, 2H), 5.69-5.62 (m, 2H), 5.13 (t, *J* = 9.8 Hz, 1H), 5.01 (dd, *J* = 10.3, 3.6 Hz, 1H), 4.22 (dd, *J* = 12.1, 4.6 Hz, 1H), 4.09-4.01 (m, 2H), 2.05 (s, 3H), 2.04 (s, 3H), 2.03 (s, 3H), 2.02 (s, 3H). ^{13}C NMR (76 MHz, $CDCl_3$): δ 170.5, 170.2, 169.3, 169.2, 155.3, 129.5, 128.5, 118.4, 94.6, 72.6, 72.1, 71.1, 68.2, 61.9, 20.7, 20.6, 20.6, 20.6. LRMS (ESI): *m/z* calcd. for $C_{20}H_{27}BrNO_{10}$ $[M+NH_4]^+$ 521.4; found, 522.2.

4-Bromophenyl-a-D-glucopyranoside (33)
Compound **33a** (25 mg, 0.050 mmol) was dissolved in a solution of sodium methoxide in methanol (2 mL) and stirred for one hour at room temperature. The solution was then neutralized with Amberlite® IR-120 (H^+) ion-exchange resin, filtered and concentrated. The filtrate was concentrated and the product was lyophilized to yield **33** as a white powder (17 mg, 98%). ^{1}H NMR (300 MHz, D_2O): δ 7.51 (d, *J* = 9.0 Hz, 2H), 7.09 (d, *J* = 9.1 Hz, 2H), 5.61 (d, *J* = 3.8 Hz, 1H), 3.90 (t, *J* = 9.4 Hz, 1H), 3.77-3.69 (m, 4H), 3.50 (t, *J* = 9.5 Hz, 1H). ^{13}C NMR (76 MHz, D_2O): δ 151.5, 128.4, 114.3, 110.8, 96.0, 71.9, 71.3, 68.7, 65.2, 56.3. LRMS (ESI): *m/z* calcd. for $C_{12}H_{15}BrNaO_6$ $[M+Na]^+$ 357.0; found, 357.2.

4-Ethoxyphenyl-2,3,4,6-tetra-O-acetyl-β-D-glucopyranoside (34a)
Compound **34a** was prepared in a similar manner as **2a** from 1,2,3,4,6-penta-*O*-acetyl-β-D-glucopyranose (300 mg, 0.769 mmol), 4-ethoxyphenol (117 mg, 0.845 mmol) with boron trifluoride diethyl etherate (1.42 mL, 11.54 mmol)**.** Column chromatography (3:1 hexanes/EtOAc) afforded **34a** as a colourless solid (327 mg, 74%). ^{1}H NMR (300 MHz, $CDCl_3$): δ 1.34 (t, *J* = 7.0 Hz, 3H), 1.99 (s, 3H), 2.00 (s, 3H), 2.03 (s, 3H), 2.04 (s, 3H), 3.74-3.81 (m, 1H), 3.94 (q, *J* = 7.0 Hz, 2H), 4.12 (dd, *J* = 12.3, 2.3 Hz, 1H), 4.25 (dd, *J* = 12.3, 5.3 Hz, 1H), 4.92 (d, *J* = 7.6 Hz, 1H), 5.11 (dd, *J* = 9.7, 9.7 Hz, 1H), 5.18-5.27 (m, 2H), 6.76 (d, *J* = 9.0 Hz, 2H), 6.89 (d, *J* = 9.0 Hz, 2H). ^{13}C NMR (75 MHz, $CDCl_3$): δ 14.9, 20.6, 20.6, 20.7,

20.7, 61.9, 63.9, 63.3, 71.2, 71.9, 72.8, 100.3, 115.2, 118.7, 150.8, 155.1, 169.3, 169.4, 170.2, 170.6. LRMS (ESI): *m/z* calcd. for $C_{22}H_{28}KO_{11}$ $[M+K]^+$ 507.1, found 507.1.

4-Ethoxyphenyl-β-D-glucopyranoside (34)
Compound **34a** (234 mg, 0.500 mmol) and potassium carbonate (7 mg, 0.050 mmol) were dissolved in methanol (10 mL) in a 50 mL round bottom flask and stirred overnight at room temperature. Amberlite® IR-120 (H^+) ion-exchange resin was added to the mixture, which was stirred for an additional 10 minutes until a pH of 5 to 6. The mixture was filtered and the solvent was removed under reduced pressure to yield **34** as white crystals (124 mg, 83%). ^1H NMR (300 MHz, D_2O): δ 1.35 (t, *J* = 7.0 Hz, 3H), 3.44-3.62 (m, 4H), 3.74 (dd, *J* = 12.4, 5.6 Hz, 1H), 3.91 (dd, *J* = 12.4, 2.1 Hz, 1H), 4.09 (q, *J* = 7.1 Hz, 2H), 5.01 (d, *J* = 7.5 Hz, 1H), 6.97 (d, *J* = 9.2 Hz, 2H), 7.10 (d, *J* = 9.2 Hz, 2H). ^{13}C NMR (125 MHz, D_2O): δ 13.8, 60.4, 64.8, 69.3, 72.8, 75.4, 76.0, 101.5, 115.8, 118.1, 150.9, 153.7. LRMS (ESI): *m/z* calcd. for $C_{14}H_{19}O_7$ $[M-H]^-$ 300.1, found 299.1.

4-Propoxyphenyl-2,3,4,6-tetra-O-acetyl-β-D-glucopyranoside (35a)
Compound **35a** was prepared in a similar manner as **2a** from 1,2,3,4,6-penta-*O*-acetyl-β-D-glucopyranose (300 mg, 0.769 mmol), 4-propoxyphenol (129 mg, 0.845 mmol) with boron trifluoride diethyl etherate (1.42 mL, 11.54 mmol)**.** Column chromatography (4:1 hexanes/EtOAc) afforded **35a** as white crystals (195 mg, 53%). ^1H NMR (500 MHz, $CDCl_3$): δ 0.98 (t, *J* = 7.5 Hz, 3H), 1.70-1.77 (m, 2H), 1.99 (s, 3H), 2.00 (s, 3H), 2.03 (s, 3H), 2.04 (s, 3H), 3.76-3.80 (m, 1H), 3.83 (t, *J* = 6.6 Hz, 2H), 4.13 (dd, *J* = 12.3, 2.4 Hz, 1H), 4.25 (dd, *J* = 12.3, 5.3 Hz, 1H), 4.92 (d, *J* = 7.7 Hz, 1H), 5.11 (dd, *J* = 9.4, 9.4 Hz, 1H), 5.17-5.26 (m, 2H), 6.77 (d, *J* = 9.1 Hz, 2H), 6.89 (d, *J* = 9.1 Hz, 2H). ^{13}C NMR (125 MHz, $CDCl_3$): δ 10.5, 20.6, 20.6, 20.7, 20.7, 22.6, 61.9, 68.3, 70.0, 71.2, 71.9, 72.7, 100.3, 115.2, 118.7, 150.8, 155.3, 169.3, 169.4, 170.3, 170.6. LRMS (ESI): *m/z* calcd. for $C_{23}H_{30}KO_{11}$ $[M+K]^+$ 521.1, found 521.1.

4-Propoxyphenyl-β-D-glucopyranoside (35)
Compound **35a** (185 mg, 0.384 mmol) and potassium carbonate (5 mg, 0.038 mmol) were dissolved in methanol (10 mL) in a 50 mL round bottom flask and stirred overnight at room temperature. Amberlite® IR-120 (H^+) ion-exchange resin was added to the mixture, which was stirred for an additional 10 minutes until a pH of 5 to 6. The mixture was filtered and the solvent was removed under reduced pressure to yield **35** as white crystals (114 mg, 94%). ^1H NMR (500 MHz, D_2O): δ 0.95 (t, *J* = 7.5 Hz, 3H), 1.68-1.75 (m, 2H), 3.43-3.57 (m, 4H), 3.71 (dd, *J* = 12.5, 5.7 Hz, 1H), 3.89 (dd, *J* = 12.4, 2.2 Hz, 1H), 3.96 (t, *J* = 6.6 Hz, 2H), 4.96 (d, *J* = 7.7 Hz, 1H), 6.95 (d, *J* = 9.2 Hz, 2H), 7.07 (d, *J* = 9.1 Hz, 2H). ^{13}C NMR (125 MHz, D_2O): δ 9.5, 21.8, 60.4, 69.3, 70.9, 72.9, 75.5, 76.0, 101.1, 115.9, 118.1, 150.9, 153.9. LRMS (ESI): *m/z* calcd. for $C_{15}H_{21}O_7$ $[M-H]^-$ 314.1, found 313.0.

2-Methoxyphenyl-2,3,4,6-tetra-O-acetyl-β-D-glucopyranoside (36a)
Compound **36a** was prepared in a similar manner as **2a** from 1,2,3,4,6-penta-*O*-acetyl-β-D-glucopyranose (300 mg, 0.769 mmol), 2-methoxyphenol (105 mg, 0.845 mmol) with boron trifluoride diethyl etherate (0.950mL, 7.69 mmol)**.** Column chromatography (3:1 hexanes/EtOAc) afforded **36a** as a white powder (175 mg, 50%). ^1H NMR (300 MHz, $CDCl_3$): δ 2.00 (s, 3H), 1.97 (s, 3H), 2.01 (s, 3H), 2.12 (s, 3H), 3.70-3.73 (m, 1H), 3.75 (s, 3H), 4.09 (dd, *J* = 12.2, 2.4 Hz, 1H), 4.22 (dd, *J* = 12.2, 5.4 Hz, 1H), 4.91 (d, *J* =7.7 Hz, 1H), 5.05-5.12 (m, 1H), 5.20-5.24 (m, 2H), 6.80 (ddd, *J* = 7.8, 7.8, 1.4 Hz, 1H), 6.84 (dd, *J* = 8.2, 1.4 Hz, 1H), 7.00 (ddd, *J* = 8.0, 8.0, 1.5 Hz, 1H), 7.05 (dd, *J* =8.0, 1.5 Hz, 1H). ^{13}C NMR (75 MHz, $CDCl_3$): δ 20.7, 20.7, 20.8, 20.9, 56.1, 62.1, 68.6, 71.4, 72.1, 72.8, 101.0, 112.9, 120.4, 121.0, 124.9, 146.3, 150.9, 169.6, 169.6, 170.5, 170.8. LRMS (ESI): *m/z* calcd. for $C_{21}H_{26}KO_{11}$ $[M+K]^+$ 493.9, found, 494.1.

2-Methoxyphenyl-β-D-glucopyranoside (36)
Compound **36a** (175 mg, 0.385 mmol) and potassium carbonate (5 mg, 0.0139 mmol) were dissolved in methanol (10 mL) in a 50 mL round bottom flask and stirred overnight at room temperature. Amberlite® IR-120 (H^+) ion exchange resin was added to the mixture, which was stirred for an additional 10 minutes

until a pH of 5 to 6. The mixture was filtered and the solvent was removed under reduced pressure to yield **36** as white crystals (63 mg, 57%). ^{1}H NMR (300 MHz, D_2O): δ 3.48- 3.52 (m, 1H), 3.55-3.62 (m, 3H), 3.73 (dd, *J* = 12.2, 5.3 Hz, 1H), 3.87 (s, 3H), 3.89 (dd, *J* = 12.2, 2.1 Hz, 1H), 5.10-5.13 (m, 1H), 6.96-7.03 (m, 1H), 7.10-7.14 (m, 2H), 7.18 (dd, *J* = 8.8, 1.2, 1H). ^{13}C NMR (75 MHz, D_2O): δ 55.8, 60.3, 69.3, 72.8, 75.5, 76.0, 112.9, 116.2, 121.5, 123.8, 145.4, 148.7. LRMS (ESI): *m/z* calcd. for $C_{13}H_{18}KO_7$ $[M+K]^+$ 325.1, found 325.2.

4-Trifluoromethoxyphenyl-2,3,4,6-tetra-O-acetyl-ß-D-galactopyranoside (37a)

Compound **37a** was prepared in a similar manner as **1a** from 1,2,3,4,6-penta-*O*-acetyl-β-D-galactopyranose (154 mg, 0.40 mmol), 4-trifluoromethoxyphenol **(**61 µL, 0.47 mmol) with boron trifluoride diethyl etherate (69 µL, 0.55 mmol). Column chromatography (4:1 hexanes/EtOAc) afforded **37a** as a white powder (125 mg, 89%). ^{1}H NMR (300 MHz, $CDCl_3$): δ 7.15 (d, *J* = 9.1 Hz, 2H), 7.03-6.98 (m, 2H), 5.48-5.45 (m, 2H), 5.10 (dd, *J* = 10.4, 3.4 Hz, 1H), 5.01 (d, *J* = 7.9 Hz, 1H), 4.26-4.12 (m, 2H), 4.05 (td, *J* = 6.6, 1.0 Hz, 1H), 2.18 (s, 3H), 2.07 (s, 3H), 2.05 (s, 3H), 2.01 (s, 3H). ^{13}C NMR (76 MHz, $CDCl_3$): δ 170.3, 170.2, 170.1, 169.3, 155.2, 144.6, 122.4, 122.1, 118.7, 118.0, 99.7, 77.2, 71.1, 70.7, 68.5, 66.8, 61.3, 20.7, 20.6, 20.5. LRMS (ESI): *m/z* calcd. for $C_{21}H_{22}F_3NaO_{11}$ $[M-H]^-$ 507.4; found, 507.2.

4-Trifluoromethoxyphenyl-ß-D-galactopyranoside (37)

Compound **37a** (125 mg, 0.25 mmol) was dissolved in a solution of sodium methoxide in methanol (5 mL) and stirred for one hour at room temperature. The solution was then neutralized with Amberlite® IR-120 (H^+) ion-exchange resin, filtered and concentrated. The filtrate was concentrated and the product was lyophilized to yield **37** as a white powder (77 mg, 92%). ^{1}H NMR (500 MHz, D_2O): δ 7.35-7.33 (m, 2H), 7.21 (d, *J* = 9.2 Hz, 2H), 5.07 (d, *J* = 7.5 Hz, 1H), 4.02 (d, *J* = 3.2 Hz, 1H), 3.89 (t, *J* = 6.2 Hz, 1H), 3.85-3.77 (m, 4H). ^{13}C NMR (126 MHz, D_2O): δ 155.2, 122.6, 117.6, 116.1, 100.9, 75.4, 72.4, 70.4, 68.4, 60.6. LRMS (ESI): *m/z* calcd. for $C_{13}H_{14}F_3NaO_7$ $[M-H]^-$ 339.2; found, 339.1.

2-Acetamidophenyl-2,3,4,6-tetra-O-acetyl-β-D-galactopyranoside (38a)

Compound **38a** was prepared in a similar manner as **1a** from 1,2,3,4,6-penta-*O*-acetyl-β-D-galactopyranose (300 mg, 0.77 mmol), 2-acetamidophenol (128 mg, 0.85 mmol) with boron trifluoride diethyl etherate (0.57 mL, 4.61 mmol). Column chromatography (3:2 hexanes/EtOAc) afforded **38a** as a colorless solid (28 mg, 8%). ^{1}H NMR (300 MHz, $CDCl_3$): δ 2.03 (s, 3H), 2.07 (s, 3H), 2.09 (s, 3H), 2.19 (s, 3H), 2.22 (s, 3H), 4.08- 4.26 (m, 3H), 5.03 (d, *J* = 8.1 Hz, 1H), 5.17 (dd, *J* = 10.6, 3.5 Hz, 1H), 5.46-5.52 (m, 2H), 6.93-7.09 (m, 3H), 7.89 (s, 1H), 8.40 (dd, *J* = 8.0, 1.7 Hz, 1H). ^{13}C NMR (75 MHz, $CDCl_3$): δ 20.5, 20.6, 20.6, 21.0, 24.5, 61.3, 66.7, 69.1, 70.1, 71.2, 99.9, 113.2, 120.2, 123.4, 123.6, 128.7, 145.0, 168.8, 169.9, 170.1, 170.3, 170.8. LRMS (ESI): *m/z* calcd. for $C_{22}H_{27}NNaO_{11}$ $[M+Na]^+$ 504.2, found 504.3.

2-Acetamidophenyl β-D-galactopyranoside (38)

Compound **38a** (25 mg, 0.0519 mmol) and potassium carbonate (0.7 mg, 0.00519 mmol) were dissolved in methanol (5 mL) in a 50 mL flame dried, round bottom flask and were stirred overnight at room temperature. Amberlite® IR-120 resin (H^+) ion-exchange resin was added to the mixture, which was stirred for an additional 10 minutes until a pH of 5 to 6. The mixture was filtered and the solvent was removed under reduced pressure. The product was purified by column chromatography (85:15 CH_2Cl_2:MeOH) to yield **38** as white crystals (9.9 mg, 61%). ^{1}H NMR (300 MHz, D_2O): δ 2.20 (s, 3H), 3.73-3.87 (m, 5H), 4.00 (d, *J* = 3.4 Hz, 1H), 5.01 (d, *J* = 7.5 Hz, 1H), 7.12-7.18 (m, 1H), 7.27 (d, *J* = 3.8 Hz, 1H), 7.64 (dt, *J* = 7.8, 1.0 Hz, 1H). ^{13}C NMR (75 MHz, D_2O): δ 22.6, 60.7, 68.4, 70.4, 72.5, 75.4, 101.6, 116.5, 123.4, 125.2, 127.1, 148.9, 173.4. LRMS (ESI): *m/z* calcd. for $C_{14}H_{19}KNO_7$ $[M+K]^+$ 313.1, found 312.9.

Ethyl 4-(2,3,4,6-tetra-O-acetyl-ß-D-galactopyranosyloxy)benzoate (39a)
Compound **39a** was prepared in a similar manner as **1a** from 1,2,3,4,6-penta-*O*-acetyl-β-D-galactopyranose (254 mg, 0.65 mmol), ethyl-4-hydroxybenzoate (119 mg, 0.716 mmol) with boron trifluoride diethyl etherate (0.80 mL, 6.5 mmol). Column chromatography (4:1 hexanes/EtOAc) afforded **39a** as a light yellow oil (27 mg, 9%). ^{1}H NMR (300 MHz, $CDCl_3$): δ 8.00 (d, *J* = 8.9 Hz, 1H), 7.01 (d, *J* = 8.9 Hz, 1H), 5.51 (dd, *J* = 10.5, 7.9 Hz, 1H), 5.46 (dd, *J* = 3.5, 0.7 Hz, 1H), 5.13 (d, *J* = 7.9 Hz, 1H), 5.12 (dd, *J* = 10.5, 3.5 Hz, 1H), 4.35 (q, *J* = 7.1 Hz, 2H), 4.24-4.06 (m, 3H), 2.18 (s, 3H), 2.06 (s, 3H), 2.05 (s, 3H), 2.01 (s, 3H), 1.37 (t, *J* = 7.1 Hz, 3H). ^{13}C NMR (75 MHz, $CDCl_3$): δ 170.5, 170.3, 170.2, 169.5, 166.1, 160.3, 131.7, 125.5, 116.2, 99.0, 71.4, 70.9, 68.6, 66.9, 61.5, 61.1, 20.8, 20.8, 20.8, 20.7, 14.5. LRMS (ESI): *m/z* calcd for $C_{23}H_{28}KO_{12}$ $[M+K]^+$ 535.3, found 535.2.

Ethyl 4-(β-D-galactopyranosyloxy)benzoate (39)
Compound **39a** (216 mg, 0.448 mmol) and potassium carbonate (6.2 mg, 0.0448 mmol) were dissolved in methanol (10 mL) in a 50 mL flame dried, round bottom flask and stirred overnight at room temperature. Amberlite® IR-120 (H^+) ion-exchange resin was added to the mixture, which was stirred for an additional 10 minutes until a pH of 5 to 6. The mixture was filtered and the solvent was removed under reduced pressure. The crude product was recrystallized in MeOH:Et_2O to yield **39** as white crystals (96 mg, 68%). ^{1}H NMR (300 MHz, D_2O): δ 8.00 (d, *J* = 8.9 Hz, 2H), 7.17 (d, *J* = 8.9 Hz, 2H), 5.15 (d, *J* = 7.3 Hz, 1H), 4.34 (q, *J* = 7.2 Hz, 2H), 4.02 (dd, *J* = 3.2, 0.7 Hz, 1H), 3.94-3.75 (m, 5H), 1.36 (t, *J* = 7.2 Hz, 3H). ^{13}C NMR (75 MHz, D_2O): δ 168.5, 160.6, 131.5, 124.0, 115.9, 99.9, 75.5, 72.4, 70.3, 68.3, 62.0, 60.6, 13.3. LRMS (ESI): *m/z* calcd. for $C_{15}H_{20}NaO_8$ $[M+Na]^+$ 351.1, found 351.1.

Isopropyl 4-(2,3,4,6-tetra-O-acetyl-ß-D-glucopyranosyloxy)benzoate (40a)
Compound **40a** was prepared in a similar manner as **2a** from 1,2,3,4,6-penta-*O*-acetyl-β-D-galactopyranose (500 mg, 1.28 mmol), **isopropyl-**4-hydroxybenzoate (460 mg, 2.56 mmol) with boron trifluoride diethyl etherate (0.47 mL, 3.84 mmol). Column chromatography (3:2 hexanes/EtOAc) afforded **40a** as a colorless oil (420 mg, 64%). ^{1}H NMR (300 MHz, $CDCl_3$): δ 7.99 (d, *J* = 8.96 Hz, 2H), 7.08 (d, *J* = 8.89 Hz, 2H), 5.82 (d, *J* = 7.84 Hz, 1H), 5.58-5.49 (m, 2H), 5.31-5.19 (m, 2H), 4.27 (t, *J* = 6.90 Hz, 1H), 4.12-4.01 (m, 2H), 2.15 (s, 3H), 2.05 (s, 3H), 2.01 (m, 3H), 1.91 (m, 3H), 1.55 (s, 1H), 1.33 (d, *J* = 6.26 Hz, 6H).). ^{13}C NMR (75 MHz, $CDCl_3$): δ 170.6, 170.5, 170.4, 170.2, 165.7, 159.8, 131.7, 125.8, 116.3, 94.7, 68.5, 68.0, 67.8, 67.7, 67.6, 61.6, 22.2, 20.9, 20.9, 20.8, 20.8. LRMS (ESI): *m/z* calcd. for $C_{24}H_{30}NaO_{12}$ $[M+Na]^+$ 533.2, found 532.9.

Isopropyl 4-(ß-D-glucopyranosyloxy)benzoate (40)
Compound **40a** (420 mg, 0.82 mmol) was dissolved in a solution of sodium methoxide in methanol (10 mL) and stirred for three hour at room temperature. The solution was then neutralized with Amberlite® IR-120 (H^+) ion-exchange resin, filtered and concentrated. The filtrate was concentrated and the product was lyophilized to yield **40** as a white powder (218 mg, 78%). ^{1}H NMR (400 MHz, D_2O): δ 7.99 (d, *J* = 8.87 Hz, 2H), 7.22 (d, *J* = 8.81 Hz, 2H), 5.78 (d, *J* = 7.54 Hz, 1H), 5.20-5.14 (m, 1H), 4.10-4.07 (m, 1H), 4.04-3.96 (m, 3H), 3.72-3.65 (m, 2H), 1.34 (d, *J* = 6.28 Hz, 6H). ^{13}C NMR (100 MHz, D_2O): δ 168.2, 160.2, 131.5, 124.3, 116.5, 96.7, 72.0, 70.1, 69.4, 69.1, 68.0, 61.0, 21.0. LRMS (ESI): *m/z* calcd. for $C_{16}H_{22}NaO_8$ $[M+Na]^+$ 365.3, found 365.5.

Ethyl 4-(2,3,4,6-tetra-O-acetyl-ß-D-glucopyranosyloxy)benzoate (41a)
Compound **41a** was prepared in a similar manner as **2a** from 1,2,3,4,6-penta-*O*-acetyl-β-D-galactopyranose (254 mg, 0.65 mmol), ethyl-4-hydroxybenzoate (119 mg, 0.716 mmol) with boron trifluoride diethyl etherate (0.80 mL, 6.5 mmol). Column chromatography (4:1 hexanes/EtOAc) afforded **41a** as a light yellow oil (33 mg, 11%). Characterization is consistent with that previously published.[17]

Ethyl 4-(β-D-glucuopyranosyloxy)benzoate (41)

Compound **41a** (33 mg, 0.066 mmol) and potassium carbonate (1 mg, 0.0066 mmol) were dissolved in methanol (3 mL) in a 10 mL flame dried, round bottom flask and stirred overnight at room temperature. Amberlite® IR-120 (H^+) ion-exchange resin was added to the mixture, which was stirred for an additional 10 minutes until a pH of 5 to 6. The mixture was filtered and the solvent was removed under reduced pressure. The crude product was recrystallized in MeOH:Et_2O to yield **41** as white crystals (13 mg, 59%). Characterization is consistent with that previously published.[17, 18]

4-Pentanamidophenyl-2,3,4,6-tetra-O-acetyl-β-D-glucopyranoside (42a)

Compound **42a** was prepared in a similar manner as **2a** from 1,2,3,4,6-penta-*O*-acetyl-β-D-glucopyranose (300 mg, 0.77 mmol), *N*-(4-hydroxyphenyl)pentanamide (163 mg, 0.846 mmol) with boron trifluoride diethyl etherate (1.42 mL, 11.5 mmol). Column chromatography (3:2 hexanes/EtOAc) afforded **42a** as a white solid (245 mg, 61%). ^{1}H NMR (500 MHz, $CDCl_3$): δ 0.93 (t, *J* = 7.4 Hz, 3H), 1.39 (hex, *J* = 7.5 Hz, 2H), 1.69 (quin, *J* = 7.4 Hz, 2H), 2.02 (s, 3H), 2.04 (s, 3H), 2.06 (s, 3H), 2.07 (s, 3H), 2.33 (d, *J* = 7.6 Hz, 2H), 3.81-3.85 (m, 1H), 4.15 (dd, *J* = 12.3, 2.4 Hz, 1H), 4.27 (dd, *J* = 12.3, 5.4 Hz, 1H), 5.00 (d, *J* = 7.6 Hz, 1H), 5.15 (dd, *J* = 9.2, 9.2 Hz, 1H), 5.21- 5.30 (m, 2H), 6.94 (d, *J* = 8.8 Hz, 2H), 7.23 (br s, 1H), 7.42 (d, *J* = 8.8 Hz, 2H). ^{13}C NMR (75 MHz, $CDCl_3$): δ 13.9, 20.6, 20.7, 20.7, 20.8, 22.4, 27.8, 37.3, 62.0, 68.3, 71.2, 72.0, 72.8, 99.7, 117.7, 121.5, 133.8, 153.4, 169.5, 169.5, 170.3 170.7, 171.8. LRMS (ESI): *m/z* calcd. for $C_{25}H_{32}NO_{11}$ $[M-H]^-$ 523.2, found 522.1.

4-Pentanamidophenyl-β-D-glucopyranoside (42)

Compound **42a** (245 mg, 0.468 mmol) and potassium carbonate (6.5mg, 0.0153 mmol) were dissolved in methanol (10 mL) in a 50 mL round bottom flask and stirred overnight at room temperature. Amberlite® IR-120 (H^+) ion-exchange resin was added to the mixture, which was stirred for an additional 10 minutes until a pH of 5 to 6. The mixture was filtered and the solvent was removed under reduced pressure to yield **42** as white crystals (147 mg, 88%). ^{1}H NMR (300 MHz, D_2O/MeOD): δ 0.97 (t, *J* = 7.4 Hz, 3H), 1.41 (hex, *J* = 7.6 Hz, 2H), 1.67 (quin, *J* = 7.6 Hz, 2H), 2.34 (t, *J* = 7.6 Hz, 2H), 3.35- 3.46 (m, 4H), 3.70 (dd, *J* = 12.1, 5.4 Hz, 1H), 3.89 (dd, *J* = 12.1, 1.9 Hz, 1H), 4.82 (m, 1H), 7.06 (d, *J* = 9.0 Hz, 2H), 7.45 (d, *J* = 9.0 Hz, 2H). ^{13}C NMR (75 MHz, D_2O/MeOD): δ 12.8, 22.0, 27.7, 36.2, 61.1, 70.0, 73.5, 76.6, 76.7, 101.3, 116.7, 121.2, 133.1, 154.3, 173.1. LRMS (ESI): *m/z* calcd. for $C_{17}H_{24}NO_7$ $[M-H]^-$ 355.2, found 354.2.

Methyl 3-(2,3,4,6-tetra-O-acetyl-ß-D-galactopyranosyloxy)benzoate (43a)

Compound **43a** was prepared in a similar manner as **1a** from 1,2,3,4,6-penta-*O*-acetyl-β-D-galactopyranose (300 mg, 0.77 mmol), methyl-3-hydroxybenzoate (129 mg, 0.846 mmol) with boron trifluoride diethyl etherate (0.57 mL, 4.6 mmol). Column chromatography (1:3 hexanes/EtOAc) afforded **43a** as a light yellow oil (274 mg, 74%). ^{1}H NMR (300 MHz, $CDCl_3$): δ 2.02 (s, 3H), 2.07 (s, 3H), 2.08 (s, 3H), 2.19 (s, 3H), 3.92 (s, 3H), 4.07-4.13 (m, 3H), 5.09- 5.15 (m, 2H), 5.46-5.54 (m, 2H), 7.20 (ddd, *J* = 8.2, 2.7, 1.1 Hz, 1H), 7.37 (t, *J* = 8.1 Hz, 1H), 7.66 (dd, *J* = 2.7, 1.6 Hz, 1H), 7.76 (dt, *J* = 7.7, 1.1 Hz, 1H). ^{13}C NMR (75 MHz, $CDCl_3$): δ 20.7, 20.8, 20.9, 52.4, 61.6, 67.1, 68.7, 70.9, 71.3, 99.5, 117.5, 122.0, 124.6, 129.7, 131.8, 156.9, 166.6, 169.6, 170.3, 170.4, 170.7. LRMS (ESI): *m/z* calcd. for $C_{22}H_{26}NaO_{12}$ $[M+Na]^+$ 505.1, found 505.2.

Methyl 3-(β-D-galactopyranosyloxy)benzoate (43)

Compound **43a** (216 mg, 0.448 mmol) and potassium carbonate (6.2 mg, 0.0448 mmol) were dissolved in methanol (10 mL) in a 50 mL flame dried, round bottom flask and stirred overnight at room temperature. Amberlite® IR-120 (H^+) ion-exchange resin was added to the mixture, which was stirred for an additional 10 minutes until a pH of 5 to 6. The mixture was filtered and the solvent was removed under reduced pressure. The crude product was recrystallized in MeOH:Et_2O to yield **43** as white crystals (96 mg, 68%). ^{1}H NMR (300 MHz, MeOD): δ 3.59 (dd, *J* = 9.7, 3.4 Hz, 1H), 3.69-3.83 (m, 4H), 3.90 (s, 3H), 3.92 (d, *J*

= 3.4 Hz, 1H), 4.91 (d, J = 7.7 Hz, 1H), 7.33-7.43 (m, 2H), 7.67 (dt, J = 7.1, 1.4 Hz, 1H), 7.74 (m, 1H). ^{13}C NMR (75 MHz, MeOD): δ 52.7, 62.3, 70.2, 72.7, 74.8, 77.0, 103.0, 122.7, 124.3, 130.6, 132.6, 159.2. LRMS (ESI): *m/z* calcd. for $C_{14}H_{18}NaO_8$ [M+Na]$^+$ 337.1, found 337.2.

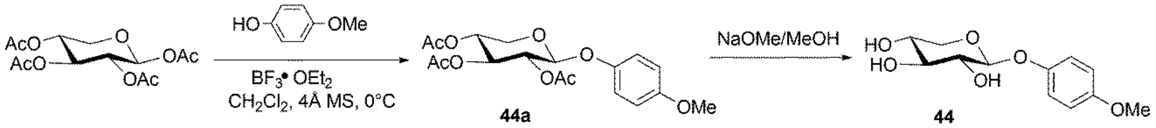

4-Methoxyphenyl-2,3,4-tri-O-acetyl-ß-D-xylopyranoside (44a)

Compound **44a** was prepared in a similar manner as **2a** from 1,2,3,4-penta-*O*-acetyl-β-D-xylopyranose (340 mg, 1.07 mmol), 4-methoxyphenol (186 mg, 1.5 mmol) with boron trifluoride diethyl etherate (402 μL, 3.2 mmol). Column chromatography (7:3 hexanes/ethyl acetate) afforded **44a** as a white powder (228 mg, 60%). Characterization data is consistent with that previously reported.[19] ^{1}H NMR (500 MHz, $CDCl_3$): δ 6.94 (d, J = 9.1 Hz, 2H), 6.82 (d, J = 9.1 Hz, 2H), 5.22 (t, J = 8.1 Hz, 1H), 5.15 (dd, J = 8.2, 6.4 Hz, 1H), 5.03-4.99 (m, 2H), 4.20 (dd, J = 12.0, 4.8 Hz, 1H), 3.77 (s, 3H), 3.47 (dd, J = 12.0, 8.1 Hz, 1H), 2.09 (s, 3H), 2.07 (s, 3H), 2.07 (s, 3H). ^{13}C NMR (126 MHz, $CDCl_3$): δ 167.0, 169.8, 169.4, 155.6, 150.6, 118.5, 114.6, 99.8, 71.0, 70.4, 68.6, 61.9, 55.6, 20.7, 20.7. LRMS (ESI): *m/z* calcd. for $C_{18}H_{22}NaO_9$ [M+Na]$^+$ 405.4; found, 405.3.

4-Methoxyphenyl-ß-D-xylopyranoside (44)

Compound **44a** (74 mg, 0.19 mmol) was dissolved in a solution of sodium methoxide in methanol (5 mL) and stirred for one hour at room temperature. The solution was then neutralized with Amberlite® IR-120 (H^+) ion-exchange resin, filtered and concentrated. The filtrate was concentrated and the product was lyophilized to yield **44** as a white powder (44 mg, 89%). Characterization data is consistent with that previously reported.[20] ^{1}H NMR (500 MHz, D_2O): δ 7.12-7.09 (m, 2H), 7.00-6.97 (m, 2H), 4.97 (d, J = 7.5 Hz, 1H), 4.00 (dd, J = 11.6, 5.5 Hz, 1H), 3.82 (s, 3H), 3.72-3.68 (m, 1H), 3.56-3.50 (m, 2H), 3.43 (dd, J = 11.5, 10.6 Hz, 1H). ^{13}C NMR (126 MHz, D_2O): δ 154.8, 150.6, 118.3, 115.0, 101.8, 75.5, 72.8, 69.0, 65.1, 55.7. LRMS (ESI): *m/z* calcd. for $C_{12}H_{16}NaO_6$ [M+Na]$^+$ 279.2; found, 279.4.

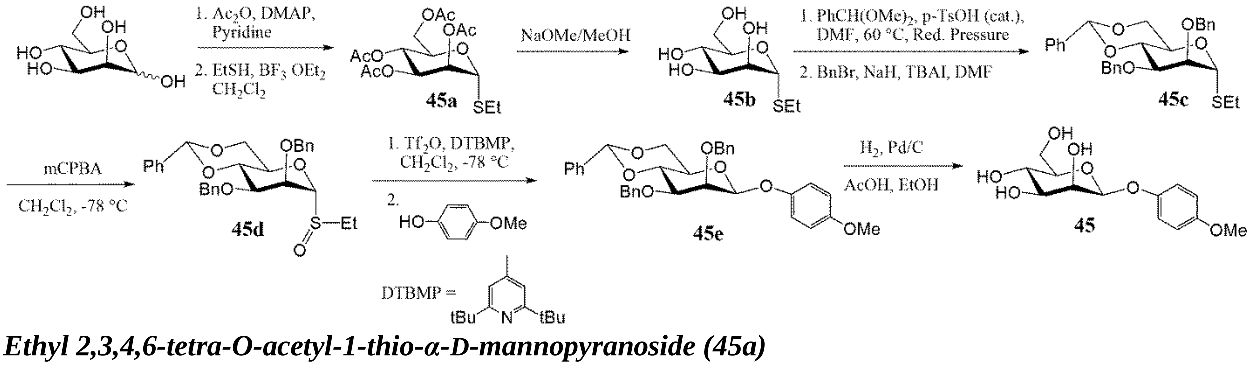

Ethyl 2,3,4,6-tetra-O-acetyl-1-thio-α-D-mannopyranoside (45a)

To a solution of D-mannose (2.0 g, 11.1 mmol) in dry pyridine (45 mL) was added acetic anhydride (26 mL) and the reaction mixture was stirred at 0 °C for 1 hour. A catalytic amount of 4-dimethylaminopyridine was added and the mixture was stirred at room temperature overnight. Ethanol was added and the solvent was evaporated under *in vacuo* and the residue was diluted in ethyl acetate, washed with sodium bicarbonate, water and brine. The mixture was dried with $MgSO_4$, filtered and concentrated. The crude product was then added to a solution of ethanethiol (1.23 mL, 16. 6 mmol) and 4 Å MS in anhydrous CH_2Cl_2 (30 mL) stirring at 0 °C under Ar and boron trifluoride diethyl etherate (4.2 mL, 33.3 mmol) was added dropwise. The reaction mixture was stirred overnight, then diluted with CH_2Cl_2 and quenched with sodium bicarbonate. The solution was filtered through Celite®, then extracted with CH_2Cl_2. The organic layer was washed with sodium bicarbonate, water, saturated brine, then dried

over $MgSO_4$ and concentrated. Column chromatography (7:3 hexanes/ethyl acetate) afforded **45a** as a syrup (2.7 g, 62%). Characterization data is consistent with that previously reported.[21] ^{1}H NMR(300 MHz, $CDCl_3$): δ 5.34 (dd, *J* = 3.2, 1.6 Hz, 1H), 5.31-5.27 (m, 3H), 4.40 (td, *J* = 7.1, 2.5 Hz,1H), 4.32 (dd, *J* = 12.1, 5.3 Hz, 1H), 4.10 (dd, *J* = 12.1, 2.2 Hz, 1H), 2.71-2.57 (m, 2H), 2.17 (s,3H), 2.10 (s, 3H), 2.05 (s, 3H), 1.99 (s, 3H), 1.31 (t, *J* = 7.4 Hz, 3H). ^{13}C NMR (75 MHz, $CDCl_3$): δ 170.6, 170.0, 169.8, 169.7, 82.2, 71.1, 69.5, 68.9, 66.3, 62.4, 25.4, 20.9, 20.7, 20.7, 20.6, 14.7. LRMS (ESI): *m/z* calcd. for $C_{16}H_{24}NaO_9S$ $[M+Na]^+$ 415.4; found, 415.2.

Ethyl 1-thio-α-D-mannopyranoside (45b)
Compound **45a** (2.7 g, 6.9 mmol) was dissolved in a solution of sodium methoxide in methanol (10 mL) and stirred for one hour at room temperature. The solution was neutralized with Amberlite® IR-120 (H^+) ion-exchange resin, filtered and concentrated to yield **45b** as a solid (1.5 g, 95%). Characterization data is consistent with that previously reported.[22] ^{1}H NMR (300 MHz, $CDCl_3$): δ 5.31 (d, *J* = 1.5 Hz, 1H), 4.03 (dd, *J* = 3.3, 1.6 Hz, 1H), 4.01-3.96 (m, 1H), 3.87 (dd, *J* = 12.3, 2.3 Hz, 1H), 3.79-3.72 (m, 2H), 3.65 (t, *J* = 9.7 Hz, 1H), 2.75-2.57 (m, 2H), 1.26 (t, *J* = 7.4 Hz, 3H). ^{13}C NMR (75 MHz, $CDCl_3$): δ 84.2, 73.0, 71.8, 71.0, 67.1, 60.8, 24.7, 14.0. LRMS (ESI): *m/z* calcd. for $C_8H_{15}O_5S$ $[M-H]^-$ 223.3; found, 223.0.

Ethyl 4,6-O-benzylidene-2,3-O-benzyl-1-thio-α-D-mannopyranoside (45c)
To a solution of **45b** (375 mg, 1.67 mmol) in DMF (3 mL) were added benzaldehyde dimethyl acetal (251 μL, 1.67 mmol) and catalytic *p*-toluenesulfonic acid. The solution was stirred under reduced pressure at 60 °C for 3 hours then cooled to room temperature and triethylamine (50 μL) was added. The mixture was then evaporated and crystallized from CH_2Cl_2/pet. ether to afford 360 mg (69%) of the crude product that was used without further purification. The crude product (166 mg, 0.53 mmol) was dissolved in DMF (5 mL) and to this solution was added NaH (85 mg, 2.12 mmol) and the mixture was stirred for 10 mins. Benzyl bromide (189 μL, 1.59 mmol) was added, followed by a catalytic amount of tetrabutylammonium iodide and the reaction mixture was stirred overnight. The mixture was quenched the following day with brine and diluted with ethyl acetate. The organic layer was washed with brine, then dried over $MgSO_4$ and concentrated. Purification by column chromatography (19:1 hexanes/ethyl acetate) yielded **45c** (221 mg, 91%, 63% overall) as a syrup. Characterization data is consistent with that previously reported.[23] ^{1}H NMR (300 MHz, $CDCl_3$): δ 7.53-7.50 (m, 2H), 7.39-7.28 (m, 13H), 5.65 (s, 1H), 5.31 (d, *J* = 0.8 Hz, 1H), 4.82-4.71 (m, 3H), 4.63 (d, *J* = 12.2 Hz, 1H), 4.32-4.16 (m, 3H), 3.94-3.87 (m, 3H), 2.68-2.48 (m, 2H), 1.24 (t, *J* = 7.4 Hz, 3H). ^{13}C NMR (76 MHz, $CDCl_3$): δ 138.4, 137.9, 137.6, 128.8, 128.4, 128.3, 128.2, 128.1, 127.8, 127.6, 127.5, 126.1, 101.4, 83.5, 79.2, 78.1, 76.4, 73.1, 73.0, 68.6, 64.6, 25.3, 14.9. LRMS (ESI): *m/z* calcd. for $C_{29}H_{33}O_5S$ $[M+H]^+$ 493.6; found, 493.2.

Ethyl 4,6-O-benzylidene-2,3-O-Benzyl-1-thio-α-D-mannopyranoside S-Oxide (45d)
To a stirred solution of **45c** (89 mg, 0.181 mmol) in dry dichloromethane (3 mL) at -78 °C was added 70% *meta*-chloroperbenzoic acid (41 mg, 0.181 mmol). The reaction mixture was stirred at -78 °C for 4 hours then warmed to 20 °C and quenched with a saturated sodium carbonate solution. The organic layer was washed with Na_2CO_3, water and brine, then dried over $MgSO_4$ and concentrated. Purification by column chromatography (2:1 Hex/EtOAc) yielded **45d** (78 mg, 85%). Characterization data is consistent with that previously reported.[24] ^{1}H NMR (300 MHz, $CDCl_3$): δ 7.47-7.25 (m, 15H), 5.61 (s, 1H), 4.85-4.77 (m, 2H), 4.71-4.64 (m, 2H), 4.59 (d, *J* = 1.4 Hz, 1H), 4.49 (dd, *J* = 3.4, 1.4 Hz, 1H), 4.32 (dd, *J* = 10.0, 9.0 Hz, 1H), 4.18 (dd, *J* = 9.8, 4.2 Hz, 1H), 4.10 (dd, *J* = 10.1, 3.4 Hz, 1H), 3.81-3.67 (m, 2H), 2.93-2.83 (m, 1H), 2.68-2.56 (m, 1H), 1.33 (t, *J* = 7.5 Hz, 3H). ^{13}C NMR (76 MHz, $CDCl_3$): δ 138.7, 137.4, 137.2, 128.6, 128.3, 128.3, 128.0, 127.9, 127.8, 127.6, 127.5, 125.6, 101.9, 92.2, 78.2, 77.1, 76.4, 73.1, 73.0, 68.3, 64.6, 45.4, 6.9. LRMS (ESI): *m/z* calcd. for $C_{29}H_{32}KO_6S$ $[M+K]^+$ 547.7; found, 547.1.

4'-Methoxyphenyl-4,6-O-benzylidene-2,3-O-benzyl-β-D-mannopyranoside (45e)
To a stirred solution of sulfoxide **45d** (17.6 mg, 0.035 mmol) and 2,6-di-*tert*-butyl-4-methylpyridine (14.2 mg, 0.069 mmol) in dry dichloromethane (1 mL) cooled to –78 °C under argon was added

trifluoromethanesulfonic anhydride (6.4 µL, 0.038 mmol). After stirring for 2-5 minutes a solution of 4-methoxyphenol (4.7 mg, 0.038 mmol) in dry dichloromethane (1 mL) was added dropwise. The reaction mixture was stirred at -78 °C for 2 hours then warmed to 0 °C over 2 hours and maintained at 0 °C for 30 minutes before quenching with saturated sodium bicarbonate, washing with brine, drying over $MgSO_4$ and concentrating. Purification by column chromatography over a gradient (19:1hexanes/ethyl acetate to 8:2 hexanes/ethyl acetate) gave a 3.5:1 β:α mixture and yielded pure β-anomer (as determined by ^{1}H NMR analysis and $J_{1,2}$ values) (10.1 mg, 53%) and pure α-anomer (2.9 mg, 15%). Characterization data for **45e** β-anomer: ^{1}H NMR (500 MHz; $CDCl_3$): δ 7.55-7.50 (m, 4H), 7.40-7.28 (m, 11H), 6.95-6.93 (m, 2H), 6.83-6.81 (m, 2H), 5.65 (s, 1H), 5.08 (d, *J* = 12.3 Hz, 1H), 5.01 (d, *J* = 12.2 Hz, 1H), 4.96 (d, *J* = 0.6 Hz, 1H), 4.75 (d, *J* = 12.5 Hz, 1H), 4.65 (d, *J* = 12.4 Hz, 1H), 4.34 (dt, *J* = 10.4, 5.2 Hz, 1H), 4.29 (d, *J* = 9.7 Hz, 1H), 4.11 (d, *J* = 3.0 Hz, 1H), 3.98 (t, *J* = 10.3 Hz, 1H), 3.77 (s, 3H), 3.68 (dd, *J* = 9.9, 3.1 Hz, 1H), 3.46-3.41 (m, 1H). ^{13}C NMR (126 MHz; $CDCl_3$): δ 155.3, 151.0, 138.2, 138.2, 137.5, 128.9, 128.7, 128.3, 128.2, 128.2, 27.7, 127.6, 127.6, 126.0, 117.9, 114.5, 101.5, 100.8, 78.5, 77.8, 76.0, 75.0, 72.6, 68.6, 67.6, 55.6. LRMS (ESI): *m/z* calcd. for $C_{34}H_{34}KO_7$ $[M+K]^+$ 593.7; found, 593.3.

4-Methoxyphenyl-ß-D-mannopyranoside (45)

A solution **45e** (8.6 mg, 0.016 mmol) in 4 mL of a 3:1 mixture of EtOH/AcOH and 5% Pd/C was stirred for 16 hours under an atmosphere of H_2. The flask was purged with air, the catalyst was removed by filtration through Celite® and the solvents were removed *in vacuo*. Purification by C-18 solid-phase extraction cartridge over a gradient (water to 9:1 water/ACN to 8:2 water/ACN to 1:1 water/ACN) afforded **45** (3.3 mg, 74%) as a white solid. ^{1}H NMR (500 MHz, D_2O): δ 7.11 (d, *J* = 9.1 Hz, 2H), 7.00 (d, *J* = 9.1 Hz, 2H), 5.28 (s, 1H), 4.20 (d, *J* = 2.9 Hz, 1H), 3.94 (dd, *J* = 12.3, 2.2 Hz, 1H), 3.82 (s, 3H), 3.79-3.74 (m, 2H), 3.68 (t, *J* = 9.7 Hz, 1H), 3.51 (ddd, *J* = 9.3, 6.5, 2.5 Hz, 1H). ^{13}C NMR (126 MHz, D_2O): δ 154.4, 150.5, 117.7, 114.9, 98.3, 76.2, 72.7, 70.5, 66.5, 60.8, 55.7. LRMS (ESI): *m/z* calcd. for $C_{13}H_{17}O_7$ $[M-H]^-$ 285.3; found, 285.1.

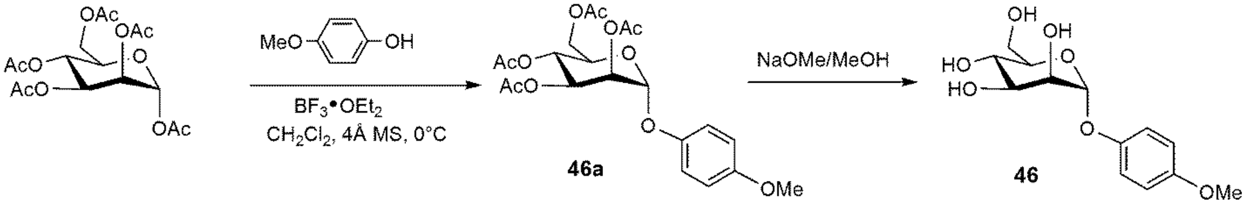

4-Methoxyphenyl-2,3,4,6-Tetra-O-Acetyl-α-D-Mannopyranoside (46a)

Compound **46a** was prepared in a similar manner as **2a** from 1,2,3,4,6-penta-*O*-acetyl-α-D-mannopyranose (250 mg, 0.64 mmol), 4-methoxyphenol (95 mg, 0.77 mmol) with boron trifluoride diethyl etherate (113 µL, 0.9 mmol). Column chromatography (3:2 hexanes/EtOAc) afforded **46a** as a white powder (124 mg, 43%). Characterization data is consistent with that previously reported.[25] ^{1}H NMR (500 MHz, $CDCl_3$): δ 7.03-7.00 (m, 2H), 6.84-6.80 (m, 2H), 5.54 (dd, *J* = 10.0, 3.5 Hz, 1H), 5.43 (dd, *J* = 3.5, 1.8 Hz, 1H), 5.41 (d, *J* = 1.8 Hz, 1H), 5.35 (t, *J* = 10.1 Hz, 1H), 4.28 (dd, *J* = 12.2, 5.3 Hz, 1H), 4.13 (ddd, *J* = 10.1, 5.3, 2.2 Hz, 1H), 4.08 (dd, *J* = 12.1, 2.3 Hz, 1H), 3.77 (s, 3H), 2.19 (s, 3H), 2.05 (s, 3H), 2.05 (s, 3H), 2.03 (s, 3H). ^{13}C NMR (126 MHz, $CDCl_3$): δ 170.6, 170.0, 169.9, 169.8, 155.4, 149.6, 117.7, 114.6, 96.6, 69.5, 69.0, 68.9, 66.0, 62.2, 55.6, 20.9, 20.7, 20.7. LRMS (ESI): *m/z* calcd. for $C_{21}H_{30}NO_{11}$ $[M+NH_4]^+$ 472.5; found, 472.4.

4-Methoxyphenyl-α-D-Mannopyranoside (46)

Compound **46a** (103 mg, 3.15 mmol) was dissolved in a solution of sodium methoxide in methanol (5 mL) and stirred for one hour at room temperature. The solution was then neutralized with Amberlite® IR-120 (H^+) ion-exchange resin, filtered and concentrated. The filtrate was concentrated and the product was lyophilized to yield **46** as a white powder (61 mg, 94%). ^{1}H NMR (500 MHz, D_2O): δ 7.13-7.09 (m, 2H), 6.98-6.94 (m, 2H), 5.47 (d, *J* = 1.8 Hz, 1H), 4.14 (dd, *J* = 3.4, 1.8 Hz, 1H), 4.00 (dd, *J* = 8.9,

3.4 Hz, 1H), 3.78-3.74 (m, 1H), 3.78 (s, 3H), 3.75-3.69 (m, 3H). ^{13}C NMR (76 MHz, D_2O): δ 154.5, 149.5, 118.7, 114.9, 99.2, 73.3, 70.3, 69.9, 66.5, 60.6, 55.7. LRMS (ESI): *m/z* calcd. for $C_{13}H_{17}O_7$ $[M-H]^-$ 285.3; found, 285.2.

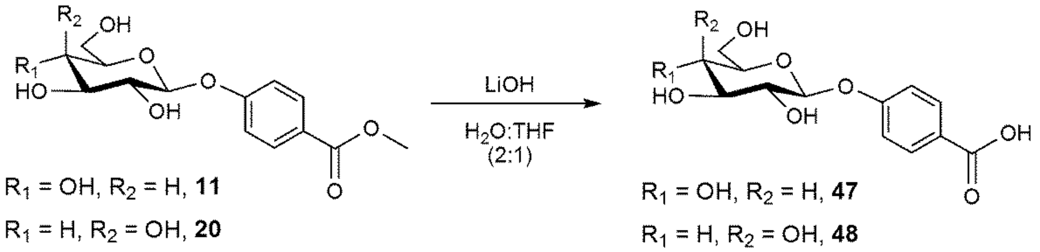

4-(β-D-Glucopyranosyloxy)-benzoic acid (47)

Compound **11** (150 mg, 0.477 mmol) and lithium hydroxide (34 mg, 1.413 mmol) was dissolved in a 2:1 mixture of H_2O:THF (10 mL) in a 50 mL flame-dried round bottom flask. The reaction mixture was stirred for 3 hours at room temperature. Amberlite IR-120 (H^+) ion-exchange resin was then added to the mixture, which was stirred for an additional 10 minutes until a pH of 5 to 6. The mixture was filtered and the solvent evaporated under reduced pressure. The crude product was dissolved in a minimum of methanol and precipitated with diethyl ether to afford **47** as white crystals (136 mg, 95%). ^1H NMR (500 MHz, D_2O): δ 3.53 (dd, *J* = 9.2, 9.2 Hz, 1H), 3.59-3.71 (m, 3H), 3.78 (dd, *J* = 12.5, 5.6 Hz, 1H), 3.96, (dd, *J* = 12.2, 1.8 Hz, 1H), 5.23 (d, *J* = 7.4 Hz, 1H), 7.17 (d, *J* = 8.7 Hz, 2H), 7.91 (d, *J* =8.7 Hz, 2H). ^{13}C NMR (125 MHz, D_2O): δ 61.1, 70.0, 73.5, 76.1, 76.8, 100.3, 116.4, 130.2, 131.8, 159.7, 174.7. LRMS (ESI): *m/z* calcd. for $C_{13}H_{15}O_8$ $[M-H]^-$ 300.1, found 298.9.

4-(β-D-Galactopyranosyloxy)-benzoic acid (48)

Compound **20** (160 mg, 0.509 mmol) and lithium hydroxide (37 mg, 1.527 mmol) were dissolved in a 2:1 mixture of H_2O:THF (10 mL) in a 50 mL flame-dried round bottom flask. The reaction mixture was stirred for 3 hours at room temperature. Amberlite IR-120 (H^+) ion-exchange resin was added to the mixture, which was stirred for an additional 10 minutes until a pH of 5 to 6. The mixture was filtered and the solvent evaporated under reduced pressure to yield **48** as white crystals (153 mg, 99%). ^1H NMR (300 MHz, D_2O): δ 3.73-3.87 (m, 4H), 3.90 (dd, *J* = 6.0, 1.0 Hz, 1H), 4.00 (dd, *J* = 3.2, 0.9 Hz, 1H), 5.14, (d, *J* = 7.3 Hz, 1H), 7.16 (dd, *J* = 8.9, 2.0 Hz, 2H), 7.96 (dd, *J* = 8.9, 2.0 Hz, 2H). ^{13}C NMR (75 MHz, D_2O): δ 60.6, 68.4, 70.4, 72.4, 75.5, 100.0, 115.9, 125.3, 131.7, 160.3, 171.1. LRMS (ESI): *m/z* calcd. for $C_{13}H_{16}KO_8$ $[M+K]^+$ 339.2, found 339.1.

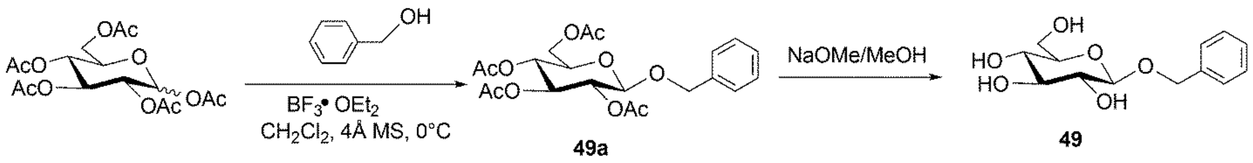

Benzyl 2,3,4,6-tetra-O-acetyl-β-D-glucopyranoside (49a)

Compound **49a** was prepared in a similar manner as **2a** from 1,2,3,4,6-penta-*O*-acetyl-D-glucopyranose, benzyl alcohol with boron trifluoride diethyl etherate**.** The crude product was recrystallized from EtOH to give **49a**. Characterization data is consistent with that previously reported.[26]

Benzyl β-D-Glucopyranoside (49)

Compound **49a** was dissolved in a solution of sodium methoxide in methanol and stirred for one hour at room temperature. The solution was then neutralized with Amberlite® IR-120 (H^+) ion-exchange resin, filtered and concentrated to give **49**. Characterization data is consistent with that previously reported.[26]

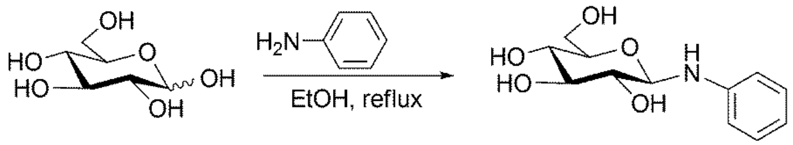

50

***N*-Phenyl-ß-D-glucopyranoside (50)**

Compound **50** was synthesized in a similar manner as **53** as previously published.[27] To a solution of D-glucose in ethanol was added aniline. The solution was refluxed for 1 h, cooled to room temperature, then 0°C to precipitate the product as a white solid. Characterization is consistent with that previously published.[28]

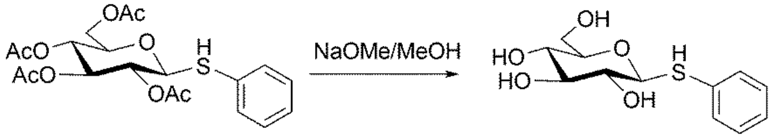

51

***S*-Phenyl-1-thio-β-D-glucopyranoside (51)**

Compound **51** was synthesized in a similar manner as **54**. S-phenyl-1-thio-β-D-glucopyranoside was dissolved in a solution of sodium methoxide in methanol and stirred for one hour at room temperature. The solution was then neutralized with Amberlite® IR-120 (H^+) ion-exchange resin, filtered and concentrated. Purification by flash chromatography (1% → 20% MeOH in CH_2Cl_2) gave **51**. Characterization data is consistent with that previously published.[28]

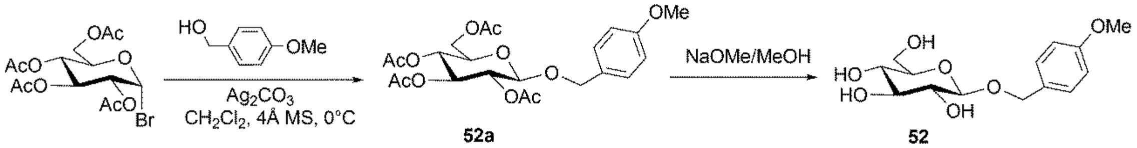

52a **52**

4-Methoxybenzyl-2,3,4,6-tetra-O-acetyl-ß-D-glucopyranoside (52a)

To a mixture of 4-methoxybenzyl alcohol (109 mg, 0.79 mmol), Ag_2CO_3 (250 mg, 0.91 mmol) and 4 Å MS in anhydrous CH_2Cl_2 (3 mL) stirring in the dark under Ar was added bromo-2,3,4,6-tetra-*O*-acetyl-β-D-glucopyranose[29] (250 mg, 0.61 mmol) in anhydrous CH_2Cl_2 (2 mL) dropwise over 20 minutes**.** The reaction mixture was diluted with CH_2Cl_2 and filtered through Celite®, then washed with sodium bicarbonate, water, saturated brine, then dried over $MgSO_4$ and concentrated. Column chromatography over a gradient (8:2 hexanes/EtOAc to 3:2 hexanes/EtOAc) afforded **52a** as a white powder (88 mg, 31%). ^{1}H NMR (400 MHz, $CDCl_3$): δ 7.22-7.19 (m, 2H), 6.89-6.85 (m, 2H), 5.18-5.07 (m, 2H), 5.03 (dd, *J* = 9.5, 8.0 Hz, 1H), 4.82 (d, *J* = 11.9 Hz, 1H), 4.56 (d, *J* = 11.9 Hz, 1H), 4.51 (d, *J* = 7.9 Hz, 1H), 4.27 (dd, *J* = 12.3, 4.7 Hz, 1H), 4.17 (dd, *J* = 12.3, 2.4 Hz, 1H), 3.81 (s, 3H), 3.66 (ddd, *J* = 9.7, 4.7, 2.5 Hz, 1H), 2.11 (s, 3H), 2.01 (s, 3H), 1.99 (s, 3H), 1.99 (s, 3H). ^{13}C NMR (101 MHz, $CDCl_3$): δ 170.7, 170.3, 169.4, 169.3, 159.5, 129.5, 128.5, 113.9, 98.8, 72.8, 71.8, 71.3, 70.4, 68.4, 62.0, 55.3, 20.8, 20.7, 20.6, 20.6. LRMS (ESI): *m/z* calcd. for $C_{22}H_{32}NO_{11}$ $[M+NH_4]^+$ 486.5; found, 486.1.

4-Methoxybenzyl-ß-D-glucopyranoside (52)

Compound **52a** (70 mg, 0.15 mmol) was dissolved in a solution of sodium methoxide in methanol (3 mL) and stirred for one hour at room temperature. The solution was then neutralized with Amberlite® IR-120 (H^+) ion-exchange resin, filtered and concentrated. The product was purified by recrystallization in hot ethyl acetate to afford **52** as a white powder (34 mg, 76%). ^{1}H NMR (400 MHz, D_2O): δ 7.44-7.41 (m, 2H), 7.05-7.01 (m, 2H), 4.88 (d, *J* = 11.3 Hz, 1H), 4.70 (d, *J* = 11.4 Hz, 1H), 4.50 (d, *J* = 8.0 Hz, 1H), 3.92 (dd, *J* = 12.3, 2.0 Hz, 1H), 3.85 (s, 3H), 3.73 (dd, *J* = 12.3, 5.7 Hz, 1H), 3.47-3.38 (m, 3H), 3.28 (dd, *J* = 9.0, 8.1 Hz, 1H). ^{13}C NMR (101 MHz, D_2O): δ 158.9, 130.6, 129.1, 114.1, 100.9, 75.9, 75.8, 73.1, 71.1, 69.7, 60.8, 55.4. LRMS (ESI): *m/z* calcd. for $C_{14}H_{24}NO_7$ $[M+NH_4]^+$ 318.3; found, 318.2.

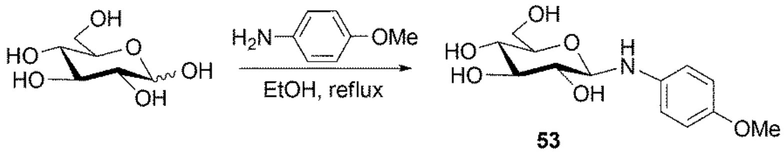

N-(4-Methoxyphenyl)-1-ß-D-glucopyranoside (53)

To a solution of D-glucose (50 mg, 0.28 mmol) in ethanol (2 mL) was added *para*-anisidine. The solution was refluxed for 1 h, cooled to room temperature, then 0°C to precipitate the product. The product was filtered and lyophilized to yield **53** as a white powder (68 mg, 86%). ^{1}H NMR (500 MHz, D_2O): δ 6.81-6.79 (m, 2H), 6.76-6.73 (m, 2H), 4.55 (d, *J* = 8.8 Hz, 1H), 3.74 (dd, *J* = 12.4, 2.3 Hz, 1H), 3.66 (s, 3H), 3.57 (dd, *J* = 12.4, 5.7 Hz, 1H), 3.45 (t, *J* = 9.1 Hz, 1H), 3.40 (ddd, *J* = 9.8, 5.7, 2.3Hz, 1H), 3.32-3.26 (m, 2H). ^{13}C NMR (126 MHz, D_2O): δ 152.4, 139.7, 115.9, 115.2, 85.7, 76.8, 76.4, 72.7, 69.7, 60.7, 55.8. LRMS (ESI): *m/z* calcd. for $C_{13}H_{19}KNO_6$ $[M+K]^+$ 324.3; found, 324.2.

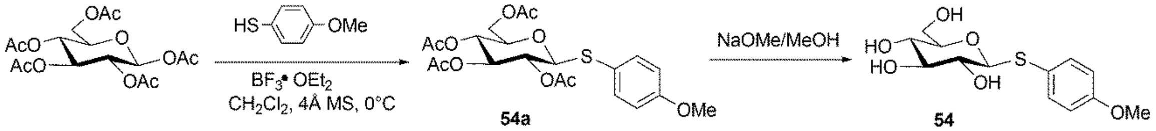

4-Methoxyphenyl-2,3,4,6-tetra-O-acetyl-1-thio-ß-D-glucopyranoside (54a)

Compound **54a** was prepared in a similar manner as **2a** from 1,2,3,4,6penta-*O*-acetyl-β-D-glucopyranose (200 mg, 0.51 mmol), 4methoxythiophenol (76 *µ*L, 0.62 mmol) with boron trifluoride diethyl etherate (97 *µ*L, 0.77 mmol)**.** Column chromatography (7:3 hexanes/EtOAc) afforded **54a** as a white powder (188 mg, 78%). Characterization data is consistent with that previously reported in the literature.[30] ^{1}H NMR (500 MHz, $CDCl_3$): δ 7.46-7.43 (m, 2H), 6.86-6.83 (m, 2H), 5.20 (t, *J* = 9.3 Hz, 1H), 5.00 (t, *J* = 9.8 Hz, 1H), 4.89 (dd, *J* = 10.0, 9.3 Hz, 1H), 4.55 (d, *J* = 10.0 Hz, 1H), 4.20-4.18 (m, 2H), 3.82 (s, 3H), 3.69-3.66 (m, 1H), 2.11 (s, 3H), 2.08 (s, 3H), 2.01 (s, 3H), 1.98 (s, 3H). ^{13}C NMR (126 MHz, $CDCl_3$): δ 170.6, 170.2, 169.4, 169.3, 136.6, 120.8, 114.4, 85.7, 75.7, 74.0, 69.8, 68.1, 62.0, 55.3, 20.2, 20.8, 20.8. LRMS (ESI): *m/z* calcd. for $C_{21}H_{27}O_{10}S$ $[M+H]^+$ 471.5; found, 471.3.

4-Methoxyphenyl-1-thio-ß-D-glucopyranoside (54)

Compound **54a** (108 mg, 0.23 mmol) was dissolved in a solution of sodium methoxide in methanol (5 mL) and stirred for one hour at room temperature. The solution was then neutralized with Amberlite® IR-120 (H^+) ion-exchange resin, filtered and concentrated. The filtrate was concentrated and the product was lyophilized to yield **54** as a white powder (59 mg, 85%). ^{1}H NMR (500 MHz, D_2O): δ 7.54 (dd, *J* = 8.9, 0.6 Hz, 2H), 6.99 (dd, *J* = 9.0, 0.6 Hz, 2H), 4.60 (d, *J* = 9.8 Hz, 2H), 3.87-3.84 (m, 1H), 3.83 (dd, *J* = 10.6, 1.2 Hz, 3H), 3.70-3.66 (m, 1H), 3.47 (td, *J* = 9.0, 0.6 Hz, 1H), 3.42-3.38 (m, 1H), 3.35-3.31 (m, 1H), 3.23 (td,*J* = 9.4, 0.6 Hz, 1H). ^{13}C NMR (126 MHz, D_2O): δ 159.4, 135.1, 122.0, 114.7, 87.7, 79.8, 77.1, 71.5, 69.3, 60.7, 55.4. LRMS (ESI): *m/z* calcd. for $C_{13}H_{17}O_6S$ $[M-H]^-$ 302.3; found, 302.0.

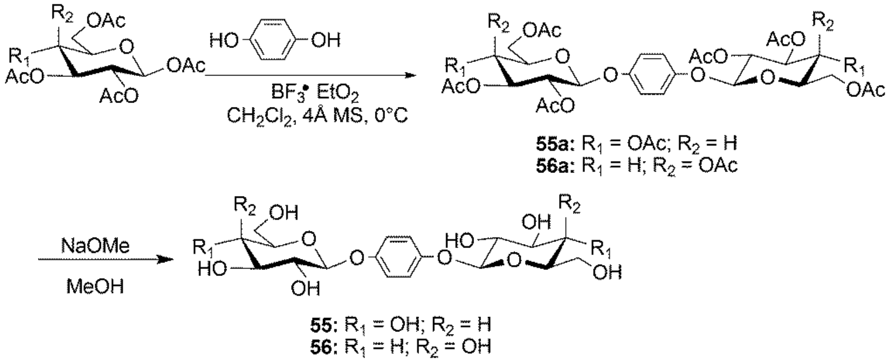

1,4-Bis-(2,3,4,6- tetraO-acetyl-ß-D-glucopyranosyl)benzene (55a)

Compound **55a** was prepared in a similar manner as **2a** from 1,2,3,4,6-penta-*O*-acetyl-β-D-glucopyranose (1 g, 2.56 mmol), hydroquinone (211 mg, 1.92 mmol) with boron trifluoride diethyl etherate (482 μL, 3.84 mmol). Column chromatography (7:3 hexanes/ethyl acetate) afforded **55a**[4] (491 mg, 33%) as a white powder. ^{1}H NMR (400 MHz, $CDCl_3$): δ 6.92 (s, 4H), 5.30-5.21 (m, 4H), 5.16 (t, *J* = 9.6 Hz, 2H), 4.98 (d, *J* = 7.6 Hz, 2H), 4.28 (dd, *J* = 12.3, 5.1 Hz, 2H), 4.16 (dd, *J* = 12.3, 2.5 Hz, 2H), 3.81 (ddd, *J* = 9.9, 5.1, 2.5 Hz, 2H), 2.07 (s, 6H), 2.06 (s, 6H), 2.04 (s, 7H), 2.03 (s, 6H). ^{13}C NMR (101 MHz, $CDCl_3$): δ 170.5, 170.2, 169.3, 169.2, 152.8, 118.4, 99.8, 72.6, 72.0, 71.1, 68.2, 61.8, 20.7, 20.6, 20.6, 20.6. LRMS (ESI): *m/z* calcd. for $C_{34}H_{42}KO_{20}$ $[M+K]^+$ 809.9; found, 809.2.

1,4-Bis-(2,3,4,6-tetra-O-acetyl-ß-D-galactopyranosyl)benzene (56a)

Compound **56a** were prepared in a similar manner as **1a** from 1,2,3,4,6-penta-*O*-acetyl-β-D-galactopyranose (1 g, 2.56 mmol), hydroquinone (211 mg, 1.92 mmol) with boron trifluoride diethyl etherate (482 μL, 3.84 mmol). Column chromatography (7:3 hexanes/ethyl acetate) afforded **56a**[4] (451 mg, 30%) as a white powder. ^{1}H NMR (400 MHz, $CDCl_3$): δ 6.94 (s, 4H), 5.48-5.44 (m, 4H), 5.11-5.07 (m, 2H), 4.95 (d, *J* = 7.9 Hz, 2H), 4.25-4.20 (m, 2H), 4.17-4.12 (m, 2H), 4.03-4.00 (m, 2H), 2.18 (s, 6H), 2.07 (s, 6H), 2.05 (s, 6H), 2.01 (s, 6H). ^{13}C NMR (101 MHz, $CDCl_3$): δ 170.3, 170.2, 170.1, 169.3, 152.9, 118.3, 100.4, 71.0, 70.8, 68.6, 66.8, 61.2, 20.7, 20.7, 20.6. LRMS (ESI): *m/z* calcd. for $C_{34}H_{46}NO_{20}$ $[M+NH_4]^+$ 788.8; found, 788.6.

1,4-Bis-(ß-D-glucopyranosyl)benzene (55)

Compound **55a** (300 mg, 0.4 mmol) was dissolved in a solution of sodium methoxide in methanol (5 mL) and stirred for one hour at room temperature. The solution was then neutralized with Amberlite® IR-120 (H^+) ion-exchange resin, filtered and concentrated. The filtrate was concentrated and the product was lyophilized to yield **55** as a white powder (160 mg, 95%). ^{1}H NMR (400 MHz, D_2O): δ 7.11 (s, 4H), 5.04 (d, *J* = 7.5 Hz, 2H), 3.91 (dd, *J* = 12.4, 2.2 Hz, 2H), 3.74 (dd, *J* = 12.5, 5.7 Hz, 2H), 3.61-3.45 (m, 8H). ^{13}C NMR (101 MHz, D_2O): δ 152.3, 118.0, 100.8, 76.1, 75.5, 72.9, 69.4, 60.5. LRMS (ESI): *m/z* calcd. for $C_{18}H_{26}NaO_{12}$ $[M+Na]^+$ 457.4; found, 457.2.

1,4-Bis-(ß-D-galactopyranosyl)benzene (56)

Compound **56a** (100 mg, 0.13 mmol) was dissolved in a solution of sodium methoxide in methanol (5 mL) and stirred for one hour at room temperature. The solution was then neutralized with Amberlite® IR-120 (H^+) ion-exchange resin, filtered and concentrated. The filtrate was concentrated and the product was lyophilized to yield **56** as a white powder (53 mg, 94%). ^{1}H NMR (400 MHz, D_2O): δ 7.11 (s, 4H), 4.98 (d, *J* = 7.0 Hz, 2H), 3.98 (d, *J* = 2.8 Hz, 2H), 3.85-3.72 (m, 11H). ^{13}C NMR (101 MHz, D_2O): δ 152.4, 117.9, 101.4, 75.4, 72.5, 70.6, 68.5, 60.7. LRMS (ESI): *m/z* calcd. for $C_{18}H_{26}NaO_{12}$ $[M+Na]^+$ 457.4; found, 457.3.

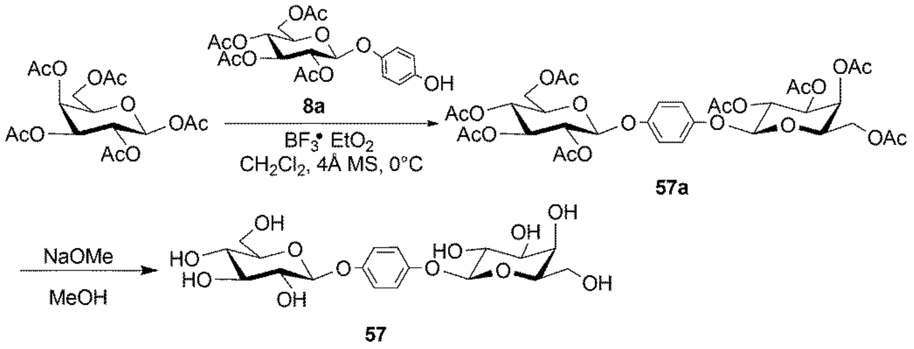

1-O-(ß-D-Glucopyranosyl)-4-O-(ß-D-galactopyranosyl)benzene (57)

Compound **57a** was prepared in a similar manner as **55a** to yield (10 mg, 0.013 mmol) which was dissolved in a solution of sodium methoxide in methanol (5 mL) and stirred for one hour at room

temperature. The solution was then neutralized with Amberlite® IR-120 (H^+) ion-exchange resin, filtered and concentrated. The filtrate was concentrated and the product was lyophilized to yield **57** as a white powder (5 mg, 94%). ^1H NMR (400 MHz, D_2O): δ 7.14-7.12 (m, 4H), 5.05 (d, *J* = 7.5 Hz, 1H), 4.99 (d, *J* = 7.2 Hz, 1H), 3.99 (dd, *J* = 3.0, 0.7 Hz, 1H), 3.92 (dd, *J* = 12.4, 2.2 Hz, 1H), 3.84-3.81 (m, 1H), 3.79-3.72 (m, 6H), 3.62-3.46 (m, 6H). ^{13}C NMR (101 MHz, D_2O): δ 152.5, 152.3, 152.2, 118.0, 117.9, 101.4, 100.9, 76.1, 75.6, 75.4, 72.9, 72.6, 70.6, 69.4, 68.5, 60.8, 60.5. LRMS (ESI): *m/z* calcd. for $C_{18}H_{26}NaO_{12}$ $[M+Na]^+$ 457.4; found, 457.0.

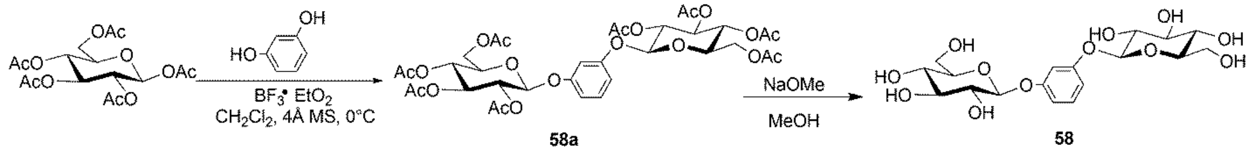

1,3-Bis-(2,3,4,6-tetra-O-acetyl-ß-D-glucopyranosyl)benzene (58a)
Compound **58a** were prepared in a similar manner as **2a** from 1,2,3,4,6-penta-*O*-acetyl-β-D-glucopyranose (1 g, 2.56 mmol), resorcinol (170 mg, 1.54 mmol) with boron trifluoride diethyl etherate (804 μL, 6.4 mmol). Column chromatography (3:2 hexanes/ethyl acetate) afforded **58a**[4] (200 mg, 17%) as a white powder. ^1H NMR (400 MHz, $CDCl_3$): δ 7.20 (t, *J* = 8.3 Hz, 1H), 6.69 (dd, *J* = 8.3, 2.3 Hz, 2H), 6.61 (d, *J* = 2.2 Hz, 1H), 5.26 (dq, *J* = 16.9, 8.6 Hz, 4H), 5.17-5.09 (m, 4H), 4.27 (dd, *J* = 12.3, 5.4 Hz, 2H), 4.15 (dd, *J* = 12.3, 2.4 Hz, 2H), 3.86 (ddd, *J* = 10.0, 5.4, 2.5 Hz, 2H), 2.07 (s, 6H), 2.05 (s, 6H), 2.04 (s, 6H), 2.03 (s, 6H). ^{13}C NMR (101 MHz, $CDCl_3$): δ 170.6, 170.2, 169.4, 169.2, 157.7, 130.1, 111.1, 106.4, 98.6, 72.7, 72.0, 71.1, 68.3, 61.9, 20.6, 20.6, 20.6, 20.6. LRMS (ESI): *m/z* calcd. for $C_{34}H_{42}NaO_{20}$ $[M+Na]^+$ 793.7; found, 793.1.

1,3-Bis-(ß-D-glucopyranosyl)benzene (58)
Compound **58a** (85 mg, 0.11 mmol) was dissolved in a solution of sodium methoxide in methanol (5 mL) and stirred for one hour at room temperature. The solution was then neutralized with Amberlite® IR-120 (H^+) ion-exchange resin, filtered and concentrated. The filtrate was concentrated and the product was lyophilized to yield **58** as a white powder (28 mg, 58%). ^1H NMR (400 MHz, D_2O): δ 7.36-7.32 (m, 1H), 6.93-6.86 (m, 3H), 5.12 (d, *J* = 7.3 Hz, 2H), 3.93 (m, 2H), 3.74 (dd, *J* = 12.4, 6.0 Hz, 3H), 3.67-3.45 (m, 9H). ^{13}C NMR (101 MHz, D_2O): δ 157.6, 130.7, 111.1, 105.0, 100.0, 76.2, 75.6, 72.9, 69.5, 60.6. LRMS (ESI): *m/z* calcd. for $C_{18}H_{30}NO_{12}$ $[M+NH_4]^+$ 452.5; found, 452.2.

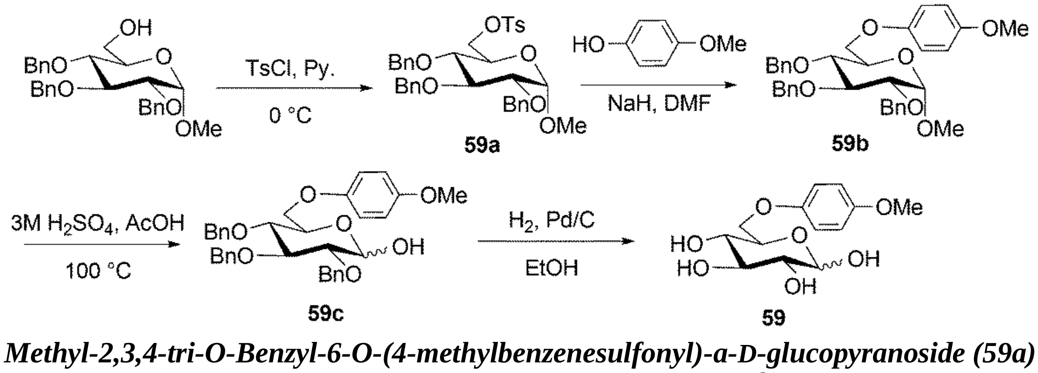

Methyl-2,3,4-tri-O-Benzyl-6-O-(4-methylbenzenesulfonyl)-a-D-glucopyranoside (59a)
To a solution of methyl-2,3,4-tri-*O*-benzyl-α-D-glucopyranoside[31] (122 mg, 0.26 mmol) and pyridine (47 μL, 0.58 mmol) in dry CH_2Cl_2 stirring at 0°C under argon was added TsCl (86 mg, 0.45 mmol). The reaction mixture was stirred overnight then diluted with CH_2Cl_2. The organic layer was washed with water, brine, then dried over $MgSO_4$ and concentrated. Column chromatography (19:1 toluene/EtOAc) afforded **59a** as a white powder (143 mg, 88%). Characterization data is consistent with that previously reported.[32] ^1H NMR (500 MHz, $CDCl_3$): δ 7.77 (d, *J* = 8.3 Hz, 2H), 7.36-7.28 (m, 15H), 7.15 (dt, *J* = 4.8, 2.3 Hz, 2H), 4.98 (d, *J* = 10.9 Hz, 1H), 4.80 (td, *J* = 14.6, 8.3 Hz, 3H), 4.64 (d, *J* = 12.1 Hz, 1H), 4.53 (d,

J = 3.5 Hz, 1H), 4.44 (d, J = 10.7 Hz, 1H), 4.20 (qd, J = 9.8, 3.2 Hz, 2H), 3.96 (t, J = 9.3 Hz, 1H), 3.76 (ddd, J = 10.1, 3.9, 2.1 Hz, 1H), 3.49-3.43 (m, 2H), 3.32 (s, 3H), 2.40 (s, 3H). ^{13}C NMR (126 MHz, $CDCl_3$): δ 144.8, 138.5, 137.9, 137.7, 132.8, 129.8, 128.5, 128.4, 128.1, 128.0, 127.9, 127.9, 127.9, 127.8, 127.6, 98.0, 81.8, 79.6, 76.8, 75.7, 74.9, 73.4, 68.5, 68.4, 55.3, 21.6. LRMS (ESI): *m/z* calcd. for $C_{35}H_{38}NaO_8S$ $[M+Na]^+$ 657.7; found, 657.5.

Methyl-2,3,4-tri-O-benzyl-6-O-(4-methoxyphenyl)-a-D-glucopyranoside (59b)

To a stirred solution of 66% sodium hydride (9 mg, 0.23 mmol) in dry DMF under argon was added *para*-methoxyphenol (26 mg, 0.21mmol). The mixture was stirred for 20 min and **59a** (65 mg, 0.11 mmol) was added and the reaction mixture was stirred overnight. The reaction was quenched with brine, diluted with ethyl acetate and the organic layer was washed with saturated sodium thiosulfate, water, brine, then dried over $MgSO_4$ and concentrated. Column chromatography (9:1 pet. ether/EtOAc) afforded **59b** as a white powder (45 mg, 75%). ^{1}H NMR (500 MHz, $CDCl_3$): δ 7.39-7.18 (m, 15H), 6.85-6.80 (m, 4H), 5.02 (d, J = 10.8 Hz, 1H), 4.86 (dt, J = 15.5, 10.6 Hz, 3H), 4.70 (d, J = 12.1 Hz, 1H), 4.66 (d, J = 3.5 Hz, 1H), 4.53 (d, J = 10.9 Hz, 1H), 4.12-4.03 (m, 3H), 3.91 (dt, J = 10.0, 2.8 Hz, 1H), 3.77 (s, 3H), 3.75 (t, J = 7.7 Hz, 1H), 3.62 (dd, J = 9.6, 3.6 Hz, 1H), 3.40 (s, 3H). ^{13}C NMR (126 MHz, $CDCl_3$): δ 153.9, 152.7, 138.7, 138.1, 138.0, 128.4, 128.4, 128.1, 128.0, 127.9, 127.7, 127.6, 115.5, 114.5, 98.2, 82.1, 79.8, 77.4, 75.8, 75.2, 73.4, 69.3, 67.1, 55.6, 55.2. LRMS (ESI): *m/z* calcd. for $C_{35}H_{38}NaO_7$ $[M+K]^+$ 609.7; found, 609.5.

2,3,4-Tri-O-benzyl-6-O-(4-methoxyphenyl)-D-glucopyranoside (59c)

A solution of **59b** (45 g, 0.08 mmol) in 3 M H_2SO_4 in acetic acid (1.2 mL) was refluxed gently for 2 h. Following this, the mixture was cooled to room temperature, diluted with water and extracted with ethyl acetate. The organic layer was extract with water, brine, then dried over $MgSO_4$ and concentrated**.** Column chromatography (4:1 hexanes/ethyl acetate) afforded **59c** as a white solid (12 mg, 27%). ^{1}H NMR (500 MHz, $CDCl_3$): δ 7.36-7.28 (m, 20H), 7.24 (d, J = 1.5 Hz, 5H), 7.18-7.16 (m, 5H), 6.84-6.79 (m, 8H), 5.26 (d, J = 3.6 Hz, 1H), 4.97 (d, J = 10.9 Hz, 3H), 4.89-4.85 (m, 4H), 4.80 (d, J = 11.8 Hz, 2H), 4.71 (d, J = 11.7 Hz, 1H), 4.55 (t, J = 10.3 Hz, 1H), 4.18 (dt, J = 10.0, 2.8 Hz, 1H), 4.09 (d, J = 2.9 Hz, 2H), 4.02 (t, J = 9.3 Hz, 2H), 3.79-3.75 (m, 9H), 3.64 (dd, J = 9.4, 3.6 Hz, 2H), 3.47-3.43 (m, 1H). ^{13}C NMR (126 MHz, $CDCl_3$): δ 154.0, 152.8, 152.7, 138.6, 138.4, 138.2, 138.0, 137.8, 137.7, 128.5, 128.5, 128.4, 128.4, 128.2, 128.1, 128.1, 128.0, 128.0, 127.9, 127.9, 127.8, 127.7, 97.6, 91.4, 84.5, 83.1, 81.7, 80.0, 77.5, 77.4, 75.8, 75.7, 75.2, 75.1, 74.8, 74.0, 73.4, 69.8, 67.6, 67.2, 55.7. LRMS (ESI): *m/z* calcd. for $C_{34}H_{36}NaO_7$ $[M+Na]^+$ 579.6; found, 579.4.

6-O-(4-Methoxyphenyl)-D-glucopyranoside (59)

To a solution of **59c** (8 mg, 0.15mmol) in ethanol (2 mL) was added Pd/C (5 mol %) and the reaction mixture was purged three times with H_2. The mixture was stirred under H_2 atmosphere overnight, filtered over Celite® and the solvent was evaporated to afforded **59** as a white powder (4 mg, 98%)**.** ^{1}H NMR (500 MHz, D_2O): δ 7.02-6.96 (m, 8H), 5.24 (d, J = 3.8 Hz, 1H), 4.67 (dd, J = 8.0, 0.6 Hz, 1H), 4.31-4.29 (m, 1H), 4.24 (dd, J = 10.9, 2.0 Hz, 1H), 4.17 (ddd, J = 18.6, 11.1, 5.4 Hz, 2H), 4.10-4.07 (m, 1H), 3.79 (d, J = 19.4 Hz, 6H), 3.73 (t, J = 9.6 Hz, 2H), 3.60-3.55 (m, 3H), 3.52-3.49 (m, 1H), 3.28 (dd, J = 9.0, 8.1 Hz, 1H). ^{13}C NMR (126 MHz, D_2O): δ 153.6, 153.5, 152.4, 152.3, 116.3, 116.3, 115.1, 96.0, 92.1, 75.6, 74.1, 74.0, 72.7, 71.4, 69.7, 69.5, 69.5, 68.0, 67.8, 55.8. LRMS (ESI): *m/z* calcd. for $C_{13}H_{18}KO_7$ $[M+K]^+$ 325.3; found, 325.1.

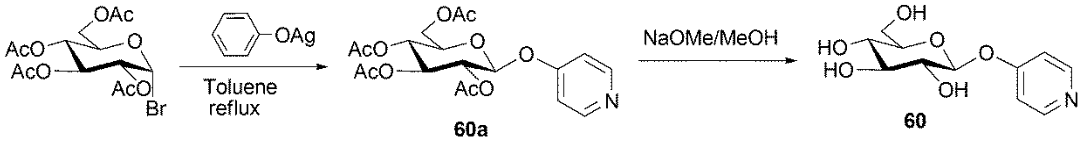

(4-Pyridinyl)-2,3,4,6-tetra-O-acetyl-β-D-glucopyranoside (60a)

Bromo-2,3,4,6-tetra-*O*-acetyl-β-D-glucopyranose[29] (250 mg, 0.61 mmol) and silver 4-pyridoxide[33] (150 mg, 0.73 mmol) were refluxed in toluene (4 mL) for one hour. After cooling the mixture was filtered through Celite®, washed with saturated aqueous sodium bicarbonate and water, dried over Mg_2SO_4, and purified by column chromatography (7:3 – 1:1 DCM/EtOAc) to give **60a** as a white solid (102 mg, 40%). %). ^{1}H NMR (500 MHz, $CDCl_3$): δ 8.47 (d, *J* = 6.2 Hz, 2H), 6.86 (d, *J* = 6.4 Hz, 2H), 5.30-5.25 (m, 2H), 5.2 (d, *J* = 6.3 Hz, 1H), 5.15 (t, *J* = 9.5 Hz, 1H), 4.26 (dd, *J* = 5.5, 12.4 Hz, 1H), 4.14 (dd, *J* = 2.5, 12.3 Hz, 1H), 3.92-3.98 (m, 1H), 2.04-2.02 (m, 12H). ^{13}C NMR (101 MHz, $CDCl_3$): δ 170.7, 170.4, 169.6, 169.4, 162.8, 151.6, 111.8, 97.5, 72.7, 72.6, 71.1, 68.3, 62.0, 20.9, 20.8. LRMS (ESI): *m/z* calcd. for $C_{19}H_{23}NaNO_{10}$ $[M+Na]^+$ 448.4, found 448.2.

(4-Pyridinyl)-β-D-glucopyranoside (60)

Compound **60a** (50 mg, 0.18 mmol) was dissolved in a solution of sodium methoxide in methanol (2 mL) and stirred for one hour at room temperature. The solution was then neutralized with Amberlite® IR-120 (H^+) ion-exchange resin, filtered and concentrated. The filtrate was concentrated and the product was lyophilized to yield **60** as a white powder (2.2 mg, 7%). ^{1}H NMR (300 MHz, D_2O): δ 8.55 (d, *J* = 5.2 Hz, 2H), 7.41 (d, *J* = 6.0 Hz, 2H), 5.41 (d, *J* = 7.1 Hz, 1H), 3.98-3.91 (m, 1H), 3.80-3.65 (m, 4H), 3.57-3.54 (m, 1H). ^{13}C NMR (101 MHz, D_2O): δ 164.2, 149.5, 137.7, 98.8, 76.3, 75.4, 76.6, 69.2, 60.4. LRMS (ESI): *m/z* calcd. for $C_{11}H_{15}NaNO_6$ $[M+Na]^+$ 280.2, found 280.4.

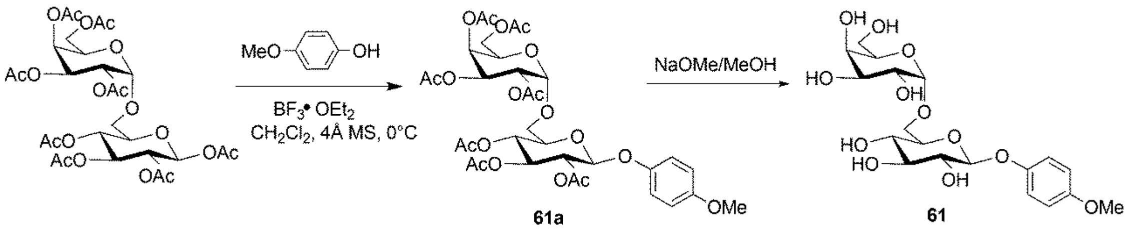

4-Methoxyphenyl-6-O-(2,3,4,6-tetra-O-acetyl-α-D-galactopyranosyl)-2,3,4-tri-O-acetyl-ß-D-glucopyranoside (61a)

Compound **61a** was prepared in a similar manner as **2a** from 6-*O*-(2,3,4,6-tetra-*O*-acetyl-α-D-galactopyranosyl)-1,2,3,4-tetra-*O*-acetyl-β-D-glucopyranoside[34] (175 mg, 0.26 mmol), 4-methoxyphenol (45 mg, 0.36 mmol) with boron trifluoride diethyl etherate (42 μL, 0.34 mmol). Column chromatography (1:1 hexanes/EtOAc) afforded **61a** as a white powder (150 mg, 78%). ^{1}H NMR (400 MHz, $CDCl_3$): δ 6.93-6.87 (m, 4H), 5.35-5.23 (m, 3H), 5.17 (dd, *J* = 9.7, 7.9 Hz, 1H), 5.10-5.00 (m, 4H), 4.15-4.12 (m, 1H), 3.89-3.88 (m, 2H), 3.84-3.74 (m, 5H), 3.54-3.51 (m, 1H), 2.09 (s, 5H), 2.05 (s, 6H), 2.01 (s, 3H), 1.98 (s, 3H), 1.94 (s, 3H). ^{13}C NMR (101 MHz, $CDCl_3$): δ 170.5, 170.3, 170.2, 170.2, 169.8, 169.4, 169.4, 155.7, 150.4, 117.7, 114.7, 98.9, 96.0, 77.2, 72.7, 71.2, 68.8, 68.1, 67.3, 66.4, 66.3, 61.5, 55.6, 20.8, 20.6, 20.7, 20.6, 20.6. LRMS (ESI): *m/z* calcd. for $C_{33}H_{44}NO_{19}$ $[M+NH_4]^+$ 761.7; found, 761.2.

4-Methoxyphenyl-6-O-(α-D-galactopyranosyl)-β-D-glucopyranoside (61)

Compound **61a** (66 mg, 0.089 mmol) was dissolved in a solution of sodium methoxide in methanol (2 mL) and stirred for one hour at room temperature. The solution was then neutralized with Amberlite® IR-120 (H^+) ion-exchange resin, filtered and concentrated. The filtrate was concentrated and the product was lyophilized to yield **61** as a white powder (28 mg, 70%). ^{1}H NMR (500 MHz, D_2O): δ 7.11 (dd, *J* = 9.2, 1.1 Hz, 2H), 6.99 (dd, *J* = 9.2, 1.1 Hz, 2H), 5.12 (d, *J* = 7.7 Hz, 1H), 4.95 (s, 1H), 3.89-3.86 (m, 1H), 3.82 (s, 3H), 3.79-3.74 (m, 6H), 3.66-3.50 (m, 6H). ^{13}C NMR (126 MHz, D_2O): δ 154.5, 150.3, 117.7, 114.9, 100.0, 97.5, 75.7, 74.5, 72.9, 70.6, 69.6, 69.3, 69.1, 68.2, 65.4, 61.0, 55.8. LRMS (ESI): *m/z* calcd. for $C_{19}H_{27}O_{12}$ $[M-H]^-$ 447.4; found, 447.2.

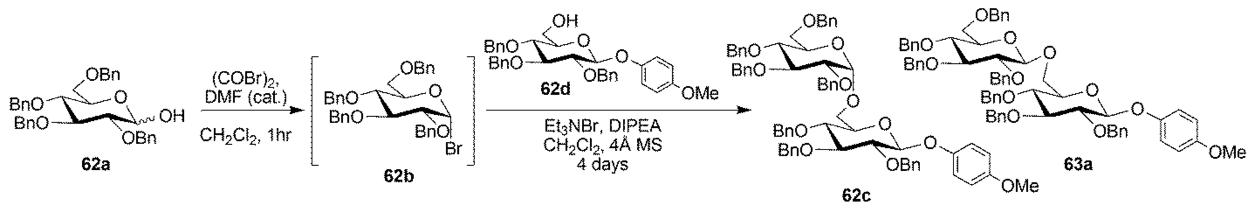

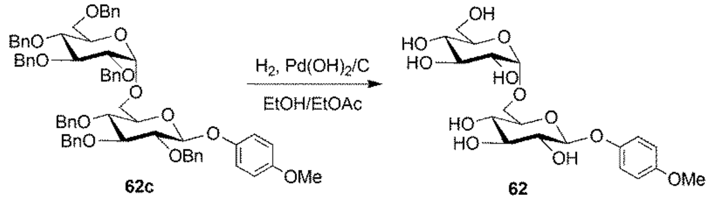

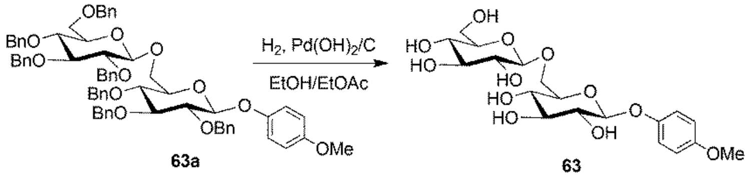

4-Methoxyphenyl-6-O-(2,3,4,6-tetra-O-benzyl-D-glucopyranosyl)-2:3,4-tri-O-benzyl-ß-D-glucopyranoside (62c and 63a)

Compound **62a**[35] was brominated as described previously.[36] To a solution of **62a** (273mg, 0.51 mmol) in dry CH_2Cl_2 (5 mL) and dry DMF (1 μL) under argon, oxalyl bromide (33 μL, 0.66 mmol) was added. The reaction mixture was stirred for 1 h, quenched with saturated $NaHCO_3$ and extracted with CH_2Cl_2. The organic phase was washed with saturated $NaHCO_3$, water, brine, dried over $MgSO_4$ and concentrated. The crude glycosyl bromide **62b** was dissolved three times with toluene and volatiles were removed as an azeotrope with water, then dissolved dry CH_2Cl_2 (1 mL) and added dropwise to a mixture of *para*-methoxyphenyl-2,3,4-tri-*O*-benzyl-β-D-glucopyranoside (compound **62d**) (140 mg, 0.25 mmol), tetraethylammonium bromide (106 mg, 0.51 mmol) and DIPEA (88 μL, 0.51 mmol) stirring in dry CH_2Cl_2 (5 mL) with 4Å molecular sieves under argon. The reaction mixture was stirred for 4 days, then diluted with CH_2Cl_2, washed with brine, dried over $MgSO_4$ and concentrated. Column chromatography over a gradient (9:1 then 7:3 hexanes/ethyl acetate) afforded a mixture of α/β (6:1) as a white solid (195 mg, 72%). Further purification with flash chromatography (50:50:1 CH_2Cl_2/hexanes/acetone) afforded pure **62c** (154 mg, 57%) and pure **63a** (18 mg, 7%).

Characterization data for 62c: ^{1}H NMR (500 MHz, $CDCl_3$): δ 7.39-7.22 (m, 30H), 7.10 (dd, *J* = 6.5, 2.8 Hz, 2H), 7.04-7.03 (m, 2H), 6.79-6.77 (m, 2H), 5.00-4.95 (m, 3H), 4.93-4.90 (m, 2H), 4.85-4.77 (m, 5H), 4.73 (dd, *J* = 11.5, 6.4 Hz, 2H), 4.66 (d, *J* = 11.1 Hz, 1H), 4.59 (d, *J* = 12.1 Hz, 1H), 4.45 (dd, *J* = 18.9, 11.6 Hz, 2H), 3.97 (t, *J* = 9.3 Hz, 1H), 3.85-3.82 (m, 2H), 3.77 (dd, *J* = 11.4, 1.4 Hz, 1H), 3.71-3.63 (m, 4H), 3.61-3.54 (m, 7H). ^{13}C NMR (126 MHz, $CDCl_3$): δ 155.4, 151.4, 138.8, 138.5, 138.5, 138.4, 138.3, 138.1, 137.9, 128.4, 128.4, 128.3, 128.3, 128.2, 128.2, 127.9, 127.9, 127.8, 127.8, 127.8, 127.7, 127.6, 127.5, 119.0, 114.6, 103.1, 97.3, 82.1, 81.8, 80.0, 77.6, 75.7, 75.1, 75.0, 74.9, 74.7, 73.3, 72.5, 70.1, 68.4, 66.0, 55.4. LRMS (ESI): *m/z* calcd. for $C_{68}H_{70}KO_{12}$ $[M+K]^+$ 1118.3; found, 1118.5.

Characterization data for 63a: ^{1}H NMR (500 MHz, $CDCl_3$): δ 7.36-7.28 (m, 30H), 7.02-6.99 (m, 2H), 6.68-6.67 (m, 2H), 5.05 (d, *J* = 10.9 Hz, 1H), 4.97-4.91 (m, 4H), 4.81 (ddd, *J* = 11.0, 7.1, 4.0 Hz, 5H), 4.67 (d, *J* = 11.0 Hz, 1H), 4.60-4.58 (m, 2H), 4.53-4.44 (m, 4H), 4.18 (d, *J* = 9.3 Hz, 1H), 3.76-3.68 (m, 7H), 3.60 (s, 4H), 3.56 (m, 1H), 3.47 (dd, *J* = 9.8, 7.0 Hz, 1H). ^{13}C NMR (126 MHz, $CDCl_3$): δ 156.2, 150.7, 138.8, 138.5, 138.5, 138.4, 138.3, 138.1, 137.9, 128.4, 128.4, 128.3, 128.3, 128.2, 128.2, 127.9, 127.9, 127.8, 127.8, 127.8, 127.7, 127.6, 127.5, 118.2, 114.8, 103.1, 99.8, 82.1, 81.2, 80.4, 77.6, 75.7, 75.1, 75.0, 74.9, 74.7, 73.9, 72.5, 71.1, 67.4, 66.0, 56.4. LRMS (ESI): *m/z* calcd. for $C_{68}H_{70}NaO_{12}$ $[M+K]^+$ 1102.3; found, 1102.0.

4-Methoxyphenyl-6-O-(α-D-glucopyranosyl)-ß-D-glucopyranoside (62)

To a solution of **62c** (117 mg, 0.11 mmol) in ethanol (4 mL) was added palladium hydroxide (5 mol %) and the reaction mixture was purged three times with H_2. The mixture was stirred under H_2 atmosphere overnight, filtered over Celite ® and the solvent was evaporated to afforded **62** as a white powder (45 mg, 93%). ^{1}H NMR (500 MHz, D_2O): δ 7.14-7.10 (m, 2H), 7.00-6.97 (m, 2H), 5.06 (d, *J* = 7.4 Hz, 1H), 4.93 (d, *J* = 3.7 Hz, 1H), 3.95 (dd, *J* = 11.4, 5.4 Hz, 1H), 3.81 (s, 3H), 3.78 (dd, *J* = 6.2, 2.2 Hz, 2H), 3.75-3.62 (m, 4H), 3.60-3.53 (m, 4H), 3.41 (t, *J* = 9.4 Hz, 1H). ^{13}C NMR (126 MHz, D_2O): δ 154.6, 150.7, 117.9, 115.0, 100.8, 97.8, 75.7, 74.4, 73.05, 72.9, 71.7, 71.4, 69.3, 69.3, 65.6, 60.3, 55.8. LRMS (ESI): *m/z* calcd. for $C_{19}H_{28}NaO_{12}$ $[M+Na]^+$ 471.4; found, 471.2.

4-Methoxyphenyl-6-O-(ß-D-glucopyranosyl)-ß-D-glucopyranoside (63)

To a solution of **63a** (117 mg, 0.11 mmol) in ethanol (4 mL) was added palladium hydroxide (5 mol %) and the reaction mixture was purged three times with H_2. The mixture was stirred under H_2 atmosphere overnight, filtered over Celite® and the solvent was evaporated to afforded **63** as a white powder (45 mg, 93%). ^{1}H NMR (500 MHz, D_2O): δ 7.15-7.11 (m, 2H), 7.01-6.98 (m, 2H), 5.06 (d, *J* = 7.9 Hz, 1H), 4.47 (d, *J* = 7.9 Hz, 1H), 4.19 (dd, *J* = 12.0, 1.7 Hz, 1H), 3.91-3.86 (m, 2H), 3.82 (s, 3H), 3.79-3.77 (m, 1H), 3.68 (dd, *J* = 12.4, 5.9 Hz, 1H), 3.60-3.52 (m, 3H), 3.44-3.26 (m, 4H). ^{13}C NMR (126 MHz, D_2O): δ 154.7, 150.7, 118.2, 115.0, 102.4, 100.9, 75.8, 75.59, 75.42, 75.33, 73.05, 72.86, 69.6, 69.3, 68.1, 60.6, 55.8. LRMS (ESI): *m/z* calcd. for $C_{19}H_{28}NaO_{12}$ $[M+Na]^+$ 471.4; found, 471.0.

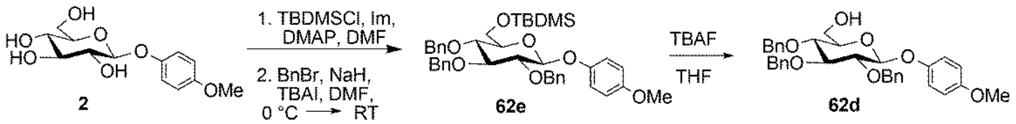

4-Methoxyphenyl-2,3,4-tri-O-benzyl-6-O-tert-butyldimethylsilyl-β-D-glucopyranoside (62e)

To a mixture of **2** (6.71 g, 23.4 mmol) in dry DMF (50 mL) under argon *tert*-butyldimethylsilyl chloride (4.24 g, 28.1 mmol), 4-dimethylaminopyridine (286 mg, 2.34 mmol) and imidazole (3.51 g, 51.5 mmol) were added. The reaction mixture was stirred overnight, then evaporated to a syrup that was dissolved in ethyl acetate and washed with 10% HCl, saturated $NaHCO_3$, water and brine, dried over $MgSO_4$ and concentrated. The crude silylated product was dissolved in 10 mL of dry DMF and added dropwise over 30 min to a mixture of 66% sodium hydride (2.46 g, 61.8 mmol), benzyl bromide (10 mL, 84.2 mmol) and tetrabutylammonium iodide (692 mg, 1.87 mmol) stirring at 0°C under argon. The reaction mixture was slowly warmed to room temperature and stirred overnight. The reaction was quenched with brine, then diluted with ethyl acetate and extracted. The aqueous layer was washed twice with ethyl acetate, and the combined organic extracts were dried over $MgSO_4$ and concentrated. Column chromatography (9:1 hexanes/ethyl acetate) afforded **62e** as a white solid (10.4 g, 83%). ^{1}H NMR (300 MHz, $CDCl_3$): δ 7.39-7.30 (m, 15H), 7.07-7.02 (m, 2H), 6.85-6.79 (m, 2H), 5.06 (d, *J* = 10.9 Hz, 1H), 4.94 (d, *J* = 10.8 Hz, 1H), 4.89-4.81 (m, 4H), 4.68 (d, *J* = 10.9 Hz, 1H), 3.88 (dd, *J* = 11.3, 1.8 Hz, 1H), 3.80-3.74 (m, 1H), 3.78 (s, 3H) 3.72-3.62 (m, 3H), 3.39 (ddd, *J* = 9.3, 4.9, 1.8 Hz, 1H), 0.90 (s, 9H), 0.05 (d, *J* = 9.5 Hz, 6H). ^{13}C NMR (76 MHz, $CDCl_3$): δ 151.6, 138.5, 138.4, 138.2, 128.4, 128.4, 128.1, 128.0, 128.0, 127.8, 127.7, 127.7, 118.7, 114.5, 103.0, 84.7, 82.3, 77.5, 76.1, 75.9, 75.0, 62.3, 55.6, 25.9, 18.3, -5.1, -5.4. LRMS (ESI): *m/z* calcd. for $C_{40}H_{54}NO_7Si$ $[M+NH_4]^+$ 688.4; found, 688.3.

4-Methoxyphenyl-2,3,4-tri-O-Benzyl-ß-D-glucopyranoside (62d)

To a solution of **62e** (10.4 g, 15.5 mmol) in dry THF under argon tetrabutylammonium fluoride (8.1 g, 30.9 mmol) was added. The reaction mixture was stirred overnight at room temperature, diluted with ethyl acetate and extracted. The organic phase was washed with brine, dried over $MgSO_4$, and concentrated. Recrystallization with hot chloroform and hexanes afforded **62d** as a white solid (8.1 g, 94%).Characterization data is consistent with that previously reported.[37] ^{1}H NMR (300 MHz, $CDCl_3$): δ

7.35-7.28 (m, 15H), 7.00-6.96 (m, 2H), 6.86-6.82 (m, 2H), 5.03 (d, J = 10.9 Hz, 1H), 4.97-4.95 (m, 2H), 4.90-4.80 (m, 3H), 4.66 (d, J = 10.9 Hz, 1H), 3.89 (ddd, J = 12.0, 6.1, 2.6 Hz, 1H), 3.77 (s, 3H), 3.75-3.62 (m, 4H), 3.47 (ddd, J = 9.6, 4.5, 2.6 Hz, 1H), 1.87 (t, J = 6.9, 1H). ^{13}C NMR (101 MHz, $CDCl_3$): δ 155.4, 151.2, 138.4, 138.1, 137.9, 128.5, 128.4, 128.2, 128.1, 128.0, 127.9, 127.8, 127.7, 118.1, 114.7, 102.5, 84.4, 82.1, 75.7, 75.3, 75.1, 75.1, 62.0, 55.6. LRMS (ESI): *m/z* calcd. for $C_{34}H_{40}NO_7$ $[M+NH_4]^+$ 574.8; found, 574.2.

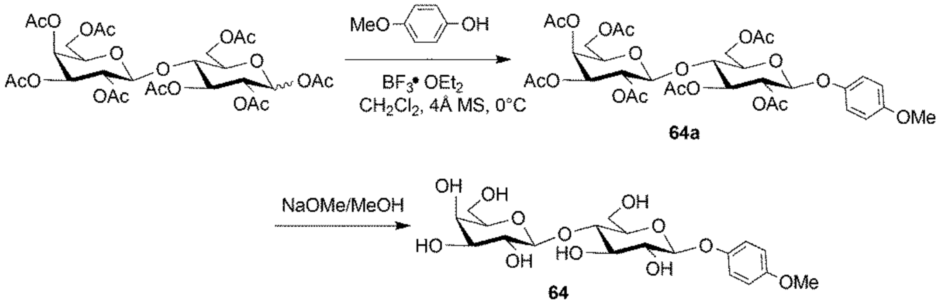

4-Methoxyphenyl-4-O-(2,3,4,6-tetra-O-acetyl-ß-D-galactopyranosyl)-2,3,6-tri-O-acetyl-ß-D-glucopyranoside (64a)

Compound **64a** was prepared in a similar manner as **2a** from 4-*O*-(2,3,4,6-tetra-*O*-acetyl-β-D-galactopyranosyl)-1,2,3,6-tetra-*O*-acetyl-β-D-glucopyranoside[34] (500 mg, 0.74 mmol), 4-methoxyphenol (128 mg, 1.03 mmol) with boron trifluoride diethyl etherate (460 µL, 3.68 mmol). Column chromatography (1:1 hexanes/EtOAc) afforded **64a** as a white powder (461 mg, 84%). ^{1}H NMR (500 MHz, $CDCl_3$): δ 6.94-6.90 (m, 2H), 6.82-6.79 (m, 2H), 5.35 (dd, J = 3.4, 0.9 Hz, 1H), 5.26 (t, J = 9.1 Hz, 1H), 5.16-5.10 (m, 2H), 3 4.96 (dd, J = 10.4, 3.4 Hz, 1H), 4.92 (d, J = 7.8 Hz, 1H), 4.51-4.49 (m, 2H), 4.16-4.12 (m, 2H), 4.08 (dd, J = 11.1, 7.4 Hz, 1H), 3.90-3.86 (m, 2H), 3.77 (s, 3H), 3.72 (ddd, J = 9.9, 5.6, 2.1 Hz, 1H), 2.16 (s, 3H), 2.10 (s, 3H), 2.07 (s, 6H), 2.07 (s, 3H), 2.05 (s, 3H), 1.97 (s, 3H). ^{13}C NMR (126 MHz, $CDCl_3$): δ 170.37, 170.32, 170.15, 170.08, 169.77, 169.63, 169.1, 155.7, 150.8, 118.6, 114.5, 101.1, 99.9, 76.2, 72.78, 72.68, 71.5, 70.9, 70.7, 69.0, 66.5, 61.9, 60.8, 55.6, 20.83, 20.73, 20.67, 20.65, 20.54. LRMS (ESI): *m/z* calcd. for $C_{33}H_{44}NO_{19}$ $[M+NH_4]^+$ 761.7; found, 761.5.

4-Methoxyphenyl-4-O-(ß-D-galactopyranosyl)-ß-D-glucopyranoside (64)

Compound **64a** (171 mg, 0.23 mmol) was dissolved in a solution of sodium methoxide in methanol (2 mL) and stirred for one hour at room temperature. The solution was then neutralized with Amberlite® IR-120 (H^+) ion-exchange resin, filtered and concentrated. The filtrate was concentrated and the product was lyophilized to yield **64** as a white powder (83 mg, 80%). ^{1}H NMR (500 MHz, D_2O): δ 6.95-6.93 (m, 2H), 6.82-6.80 (m, 2H), 4.87 (d, J = 7.9 Hz, 1H), 4.30 (d, J = 7.8 Hz, 1H),3.82-3.79 (m, 1H), 3.75 (d, J = 3.4 Hz, 1H), 3.67 (d, J = 4.3 Hz, 1H), 3.63 (s, 3H), 3.61-3.55 (m, 6H), 3.49 (dd, J = 10.0, 3.4 Hz, 1H), 3.42-3.36 (m, 3H). ^{13}C NMR (126 MHz, D_2O): δ 154.6, 150.7, 118.1, 114.9, 102.8, 100.9, 77.9, 75.3, 74.8, 74.1, 72.55, 72.39, 70.8, 68.4, 60.9, 59.7, 55.7. LRMS (ESI): *m/z* calcd. for $C_{19}H_{28}NaO_{12}$ $[M+Na]^+$ 471.4; found, 471.0.

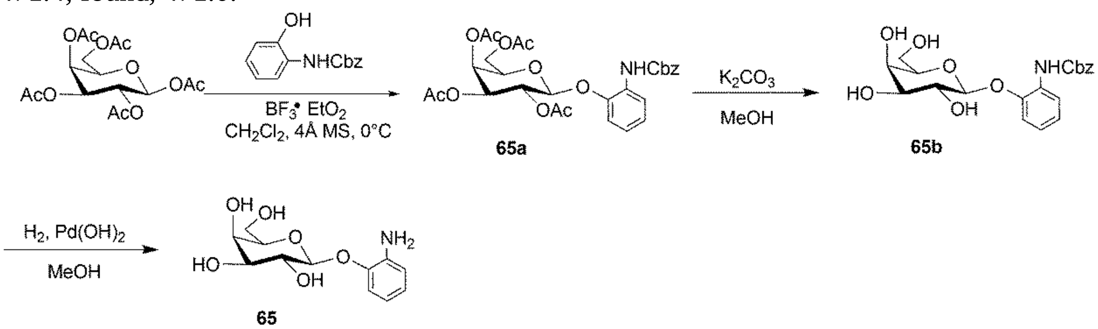

2-(Benzyloxycarbonyl)amino-2,3,4,6-tetra-O-acetyl-β-D-galactopyranoside (65a)

Compound **65a** was prepared in a similar manner as **1a** from 1,2,3,4,6-penta-*O*-acetyl-β-D-galactopyranoside (300 mg, 0.77 mmol), 2-(benzyloxycarbonylamino)-phenol (206 mg, 0.85 mmol) with boron trifluoride diethyl etherate (570 μL, 4.61 mmol). Column chromatography (8:2 – 7:3 hexanes/EtOAc) afforded **65a** (80 mg, 18%). ^{1}H NMR (300 MHz, $CDCl_3$): δ 1.95 (s, 3H), 2.00 (s, 3H), 2.03 (s, 3H), 2.04 (s, 3H), 4.01-4.21 (m, 4H), 4.94 (d, *J* =8.0 Hz, 1H), 5.10 (dd, *J* = 10.6, 3.4 Hz, 1H), 5.21 (dd, *J* = 28.7, 12.2 Hz, 2H), 5.44 (d, *J* = 4.3 Hz, 1H), 6.95 (td, *J* = 7.2, 1.6 Hz, 1H), 7.02 (dd, *J* = 8.2, 1.8 Hz, 1H), 7.08 (td, *J* = 8.3, 1.8 Hz, 1H), 7.30-7.46 (m, 6H), 8.14 (d, *J* = 7.9 Hz, 1H). ^{13}C NMR (75 MHz, $CDCl_3$): δ 20.5, 20.6, 20.7, 61.2, 66.7, 66.8, 68.8, 70.4, 71.1, 100.8, 115.6, 118.9, 122.8, 124.3, 128.1, 128.2, 128.5, 129.2, 136.2, 145.1, 153.2, 170.0, 170.1, 170.2, 170.3. LRMS (ESI): *m/z* calcd. for$C_{28}H_{31}NaNO_{12}$ $[M+Na]^+$ 596.2, found 596.3.

2-(Benzyloxycarbonyl)amino-β-D-galactopyranoside (65b)

Compound **65a** (80 mg, 0.139 mmol) and potassium carbonate (2 mg, 0.0139 mmol) were dissolved in methanol (5 mL) in a 50 mL flame dried, round bottom flask charged with an atmosphere of argon. The contents were stirred overnight at room temperature. Amberlite® IR-120 (H^+) ion-exchange resin was added to the mixture and stirred for 10 minutes to obtain pH 7. The mixture was filtered and the solvent was removed under reduced pressure to yield **65b** as a clear amorphous solid (50 mg, 89%). ^{1}H NMR (300 MHz, $(CD_3)_2CO$)): δ 3.59-3.67 (m, 1H), 3.70 (td, *J* = 5.8, 1.1 Hz, 1H), 3.78-3.98 (m, 6H), 4.14 (d, *J* = 5.7 Hz, 1H), 4.72 (d, *J* = 7.8 Hz, 1H), 4.94 (d, *J* = 4.2 Hz, 1H), 5.19 (s, 2H), 6.97 (td, *J* = 7.6, 1.8 Hz, 1H), 7.05 (td, *J* = 7.8, 1.6 Hz, 1H), 7.25 (dd, *J* = 8.0, 1.6 Hz, 1H), 7.30-7.47 (m, 5H), 8.09 (dd, *J* = 8.1, 1.4 Hz, 1H), 8.41 (s, 1H). ^{13}C NMR (75 MHz, $CDCl_3$): δ 62.2, 67.0, 69.8, 72.1, 74.5, 76.7, 104.9, 119.1, 119.9, 123.7, 124.2, 128.8, 128.9, 129.3, 130.9, 137.8, 147.6, 154.3. LRMS (ESI): *m/z* calcd. for $C_{20}H_{23}KNO_8$ $[M+K]^+$ 444.1, found 444.2.

2-Amino-β-D-galactopyranoside (65)

Compound **65b** (50 mg, 0.123 mmol) was dissolved in methanol (5 mL) in a 50 mL flame-dried round bottom flask. Paladium (II) hydroxide (1.7 mg, 0.0123 mmol) was added, the atmosphere was removed from the flask using a water aspirator, and a positive pressure of H_2 was added. The contents were stirred overnight at room temperature, after which the mixture was filtered over a bed of celite and the methanol evaporated under reduced pressure. The crude product was recrystallized in MeOH:Et_2O to yield **65** as light brown crystals (10 mg, 30%) ^{1}H NMR (300 MHz, D_2O): δ 3.72-3.84 (m, 5H), 3.98 (d, *J* = 3.4 Hz, 1H), 4.95 (d, *J* = 7.6 Hz, 1H), 6.84 (ddd, *J* = 8.1, 7.2, 1.9 Hz, 1H), 6.91 (dd, *J* = 7.8, 1.7 Hz, 1H), 6.99 (ddd, *J* = 7.9, 7.6, 1.4 Hz, 1H), 7.13 (dd, *J* = 8.1, 1.4 Hz, 1H). ^{13}C NMR (75 MHz, D_2O): δ 60.8, 68.4, 70.5, 72.5, 75.3, 102.0, 116.6, 117.4, 119.9, 124.1, 136.7, 145.2. LRMS (ESI): *m/z* calcd. for $C_{12}H_{17}NaNO_6$ $[M+Na]^+$ 294.3, found 294.2.

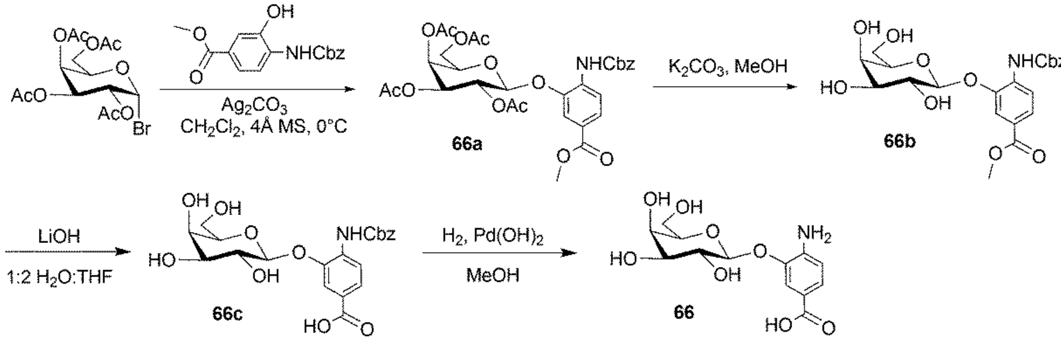

2-(Benzyloxycarbonyl)amino-methyl-3-benzoate-2,3,4,6-tetra-O-acetyl-β-D-galactopyranoside (66a)
To a mixture of methyl-4-(benzyloxycarbonylamino)-3-hydroxybenzoate (40 mg, 0.13 mmol), Ag_2CO_3 (50 mg, 0.18 mmol) and 4 Å MS in anhydrous CH_2Cl_2 (10 mL) stirring at 0 °C under Ar was added bromo-2,3,4,6-tetra-*O*-acetyl-β-D-glucopyranose[29] (250 mg, 0.61 mmol) in anhydrous CH_2Cl_2 (2 mL) dropwise over 20 minutes**.** The mixture was stirred overnight at room temperature, diluted with CH_2Cl_2 and filtered through Celite**®**, then washed with sodium bicarbonate, water, saturated brine, then dried over $MgSO_4$ and concentrated. Column chromatography over a gradient (8:2 hexanes/EtOAc to 3:2 hexanes/EtOAc) afforded **66a** as a yellow oil (11 mg, 14%). ^{1}H NMR (500 MHz, $CDCl_3$): δ 1.98 (s, 3H), 2.00 (s, 3H), 2.07 (s, 3H), 2.16 (s, 3H), 3.87 (s, 3H), 4.11 (t, *J* = 5.6 Hz, 1H), 4.17 (d, *J* = 6.6 Hz, 2H), 5.03 (d, J = 7.9 Hz, 1H), 5.12 (dd, *J* = 10.8, 3.31 Hz, 1H), 5.18 (d, *J* = 12.4 Hz, 1H), 5.55 (m, 2H), 7.29-7.45 (m, 5H), 7.55 (s, 1H), 7.66 (d, *J* = 1.7 Hz, 1H), 7.76 (dd, *J* = 8.7, 1.5 Hz, 1H), 8.23 (d, *J* = 8.7 Hz, 1H). ^{13}C NMR (125 MHz, $CDCl_3$): δ 20.5, 20.6, 20.7, 29.7, 30.9, 52.2, 61.5, 66.8, 67.2, 68.8, 70.2, 71.4, 100.3, 115.9, 117.7, 124.3, 126.0, 128.1, 128.3, 128.5, 133.4, 135.9, 144.3, 153.0, 166.4, 170.0, 170.2, 170.3, 170.78 LRMS (ESI): *m/z* calcd. for $C_{30}H_{33}NaNO_{14}$ $[M+Na]^+$ 654.1, found 654.3.

2-(Benzyloxycarbonyl)amino-methyl-3-benzoate-β-D-galactopyranoside (66b)
Compound **66a** (200 mg, 0.32 mmol) and potassium carbonate (4.4 mg, 0.032 mmol) were dissolved in methanol (5 mL) in a 50 mL round bottom flask and stirred overnight at room temperature. Amberlite IR-120 (H^+) ion-exchange resin was then added to the mixture, which was stirred for an additional 10 minutes until a pH of 5 to 6. The mixture was filtered and the solvent was removed under reduced pressure. The crude product was purified by column chromatography (95:5 CH_2Cl_2:MeOH) to afford **66b** as a yellow oil. ^{1}H NMR (500 MHz, MeOD): δ 3.59 (dd, *J* = 9.6, 3.4 Hz, 1H), 3.67 (t, *J* = 6.4 Hz, 1H), 3.77 (d, *J* = 6.1 Hz, 2H), 3.84 (dd, *J* = 10.1, 7.9 Hz, 1H), 3.86 (s, 3H), 3.92 (d, *J* = 3.7 Hz, 1H), 4.79 (d, *J* = 7.8 Hz, 1H), 5.21 (s, 2H), 7.30-7.43 (m, 5H), 7.71 (dd, *J* = 8.6, 1.9 Hz, 1H), 7.85 (d, *J* = 1.9 Hz, 1H), 8.16 (d, *J* = 8.7 Hz, 1H). ^{13}C NMR (125 MHz, MeOD): δ 49.6, 59.2, 65.1, 67.1, 69.1, 71.7, 74.2, 101.8, 116.4, 116.6, 122.7, 123.2, 126.3, 126.4, 126.6, 132.6, 134.7, 144.3, 152.2, 165.1. LRMS (ESI): *m/z* calcd. for $C_{22}H_{25}KNO_{10}$ $[M+K]^+$ 463.4, found 463.0.

2-(Benzyloxycarbonyl)amino-3-(β-D-galactopyranosyloxy)-benzoic acid (66c)
Compound **66b** (94 mg, 0.203 mmol) and lithium hydroxide (9.7 mg, 0.406 mmol) was dissolved in 2:1 mixture of THF:H_2O (6 mL) in a 50 mL flame dried round bottom flask. The reaction mixture was stirred overnight at room temperature, and then Amberlite® IR-120 (H+) ion exchange-resin was added to the mixture and stirred for 10 addition minutes. The mixture was filtered and the solvent evaporated under reduced pressure. The crude product was purified by column chromatography (4:1 DCM:MeOH) to yield **66c** as a yellow oil (54 mg, 59%). ^{1}H NMR (300 MHz, MeOD): δ 3.61 (dd, *J* = 9.8, 3.4 Hz, 1H), 3.68 (t, *J* = 5.7 Hz, 1H), 3.77 (d, *J* = 6.0 Hz, 2H), 3.81 (dd, *J* = 10.8, 8.1 Hz, 1H), 3.94 (d, *J* = 3.0 Hz, 1H), 4.80 (d, *J* = 7.8 Hz, 1H), 5.21 (s, 2H), 7.29-7.45 (m, 5H), 7.73 (dd, *J* = 8.8, 1.6 Hz, 1H), 7.85 (d, *J* = 1.6 Hz, 1H), 8.13 (d, *J* = 8.5 Hz, 1H). ^{13}C NMR (75 MHz, MeOD): δ 62.2, 68.1, 70.1, 72.2, 74.7, 77.1, 104.8, 119.6, 119.6, 126.4, 129.3, 129.4, 129.6, 135.1, 137.8, 147.3, 155.2. LRMS (ESI): *m/z* calcd. for $C_{21}H_{23}KNO_{10}$ $[M+K]^+$ 449.1, found 449.2.

2-Amino-3-(β-D-galactopyranosyloxy)-benzoic acid (66)
Compound **66c** (54 mg, 0.120 mmol) was dissolved in methanol (10 mL) in a 50 mL flame-dried round bottom flask. Paladium (II) hydroxide (2 mg, 0.0142 mmol) was added, the atmosphere was removed from the flask using a water aspirator, and a positive pressure of H_2 gas was added. The contents were stirred overnight at room temperature, after which the mixture was filtered over a bed of celite and the

methanol evaporated under reduced pressure. The resulting residue was purified on a preparative TLC plate (90:10 - 20:80 DCM:MeOH) to yield **66** as brown crystals. ^{1}H NMR (300 MHz, D_2O): δ 3.57-3.71 (m, 4H), 3.83 (d, *J* = 2.9 Hz, 1H), 4.83 (d, *J* = 7.7, 1H), 6.72 (d, *J* = 8.2 Hz, 1H), 7.33 (dd, *J* = 8.2, 1.8 Hz, 1H), 7.45 (d, *J* = 1.8 Hz, 1H). ^{13}C NMR (125 MHz, DMSO-*d*$_6$): δ 50.7, 60.4, 68.2, 70.9, 73.2, 75.7, 104.0, 113.2, 118.9, 126.1, 143.6, 162.8, 167.5. LRMS (ESI): *m/z* calcd. for $C_{13}H_{17}NaNO_8$ $[M+Na]^+$ 315.3, found 315.7.

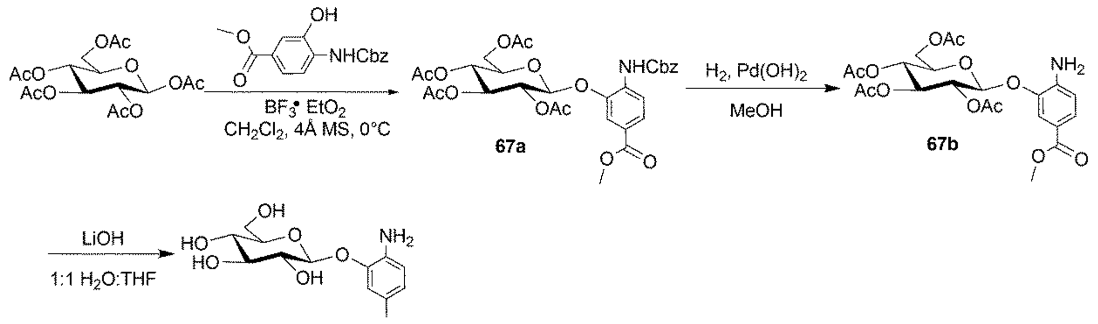

2-(Benzyloxycarbonyl)amino-methyl-3-benzoate-2,3,4,6-tetra-O-acetyl-β-D-glucopyranoside (67a)
Compound **67a** was prepared in a similar manner as **2a** from 1,2,3,4,6-penta-*O*-acetyl-β-D-glucopyranoside (100 mg, 0.26 mmol), methyl 4-(benzyloxycarbonylamino)-3-hydroxybenzoate (93 mg, 0.31 mmol) with boron trifluoride diethyl etherate (94 *μ*L, 0.72 mmol). Column chromatography (17:3 – 7:3 PE/EtOAc) afforded **67a** (44 mg, 28%). ^{1}H NMR (300 MHz, $CDCl_3$): δ 1.97 (s, 3H), 2.02 (s, 3H), 2.04 (s, 3H), 2.06 (s, 3H), 3.87 (s, 3H), 3.90-3.95 (m, 1H), 4.16 (dd, *J*= 12.3, 5.7 Hz, 1H), 5.05 (d, *J* = 7.4 Hz, 1H), 5.15 (t, *J* = 4.6 Hz, 1H), 5.24-5.34 (m, 2H), 7.31-7.45 (m, 5H), 7.50 (s, 1H), 7.67 (d, *J* = 1.7 Hz, 1H), 7.78 (dd, *J* = 8.6, 1.7 Hz, 1H), 8.24 (d, *J* = 6.4 Hz, 1H). ^{13}C NMR (75 MHz, $CDCl_3$): δ 52.3, 61.9, 67.3, 68.3, 71.3, 72.2, 72.4, 100.3, 116.6, 117.9, 124.5, 126.3, 128.3, 128.5, 128.5, 133.7, 134.0, 144.4, 153.0, 166.3, 169.6, 170.0, 170.1, 170.8. LRMS (ESI): *m/z* calcd. for $C_{30}H_{33}NaNO_{14}$ $[M+Na]^+$ 631.2, found 632.0.

2-Amino-methyl-3-benzoate-2,3,4,6-tetra-O-acetyl-β-D-glucopyranoside (67b)
Compound **67a** (44 mg, 0.0697 mmol) was dissolved in methanol (15 mL) in a 50 mL flame-dried round bottom flask. Paladium (II) hydroxide (0.98 mg, 0.00697 mmol) was added, the atmosphere was removed from the flask using a water aspirator, and a positive pressure of H_2 gas was added. The contents were stirred overnight at room temperature, after which the mixture was filtered over a bed of celite and the methanol evaporated under reduced pressure to obtain **67b** which was reacted crude in the following step.

2-Amino-3-(β-D-glucopyranosyloxy)-benzoic acid (67)
Compound **67b** (22 mg, 0.0443 mmol) and lithium hydroxide (11 mg, 0.443 mmol) were dissolved in a 1:1 mixture of THF:H_2O (10 mL) in a 50 mL flame-dried round bottom flask. The reaction mixture was stirred overnight at room temperature. Amberlite® IR-120 (H^+) ion-exchange resin was added and stirred for 10 minutes. The mixture was filtered and the solvent evaporated under reduced pressure to yield **67** (2.8 mg, 20%). ^{1}H NMR (300 MHz, $CDCl_3$): δ 3.35-3.40 (m, 1H), 3.47-3.51 (m, 3H), 3.61 (dd, *J* = 12.4, 5.8 Hz, 1H), 3.79 (dd, *J* = 12.4, 2.2 Hz, 1H), 5.01 (t, *J* = 4.0 Hz, 1H), 6.98 (d, *J* = 8.3 Hz, 1H), 7.56 (dd, *J* = 8.3, 1.8 Hz, 1H), 7.63 (d, *J* = 1.8 Hz, 1H). ^{13}C NMR (75 MHz, $CDCl_3$): δ 60.3, 69.3, 72.7, 75.4, 76.2,

101.0, 117.3, 118.4, 123.5, 126.1, 145.5, 169.9. LRMS (ESI): *m/z* calcd. for $C_{13}H_{17}NaNO_8$ $[M+Na]^+$ 315.1, found 315.2.

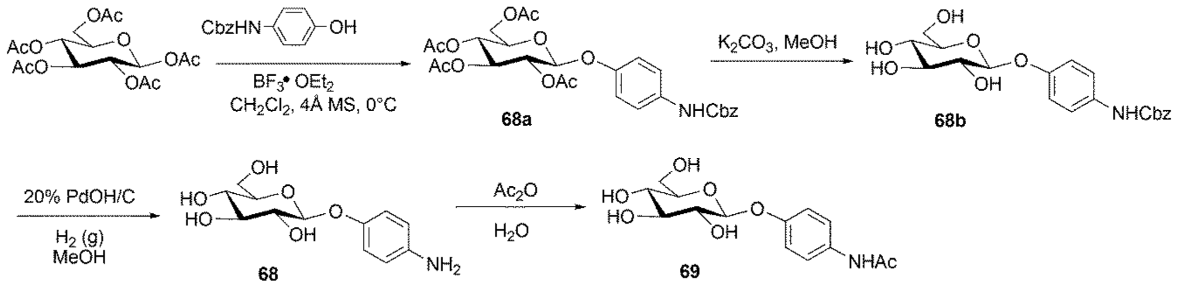

4-(Benzyloxycarbonyl)aminophenyl-2,3,4,6-tetra-O-acetyl-β-D-glucopyranoside (68a)

Compound **68a** was prepared in a similar manner as **2a** from 1,2,3,4,6-penta-*O*-acetyl-β-D-glucopyranose (300 mg, 0.77 mmol), 4-(benzyloxycarbonyl)aminophenol **(**206 mg, 0.85 mmol) with boron trifluoride diethyl etherate (1.09 mL, 15.37 mmol). Column chromatography (3:1 hexanes/EtOAc) afforded **68a** as a yellow oil (372 mg, 84%). ^{1}H NMR (300 MHz, $CDCl_3$): δ 2.03 (s, 3H), 2.04 (s, 3H), 2.06 (s, 3H), 2.07 (s, 3H), 3.79-3.86 (m, 1H), 4.15 (dd, *J* = 12.3, 2.4 Hz, 1H), 4.28 (dd, *J* = 12.2, 5.4 Hz, 1H), 5.00 (d, *J* = 7.6 Hz, 1H), 5.12-5.17 (m, 1H), 5.19 (s, 2H), 5.23-5.29 (m, 2H), 6.69 (br s, 1H), 6.95 (d, *J* = 9.0 Hz, 2H), 7.30 (d, *J* = 8.9 Hz, 2H), 7.35-7.42 (m, 5H). ^{13}C NMR (75 MHz, $CDCl_3$): δ 20.5, 20.5, 20.5, 20.6, 61.9, 68.2, 71.2, 71.8, 172.7, 99.5, 117.7, 120.2, 128.2, 128.3, 128.5, 133.6, 136.0, 152.9, 166.2, 169.5, 170.3, 170.7, 175.6. LRMS (ESI): *m/z* calcd. for $C_{28}H_{30}NO_{12}$ $[M-H]^-$ 573.2, found 572.0.

4-(Benzyloxycarbonylamino)phenyl-β-D-glucopyranoside (68b)

Compound **68a** (370 mg, 0.645 mmol) and potassium carbonate (9 mg, 0.0645 mmol) were dissolved in methanol (10 mL) in a 50 mL round bottom flask and stirred overnight at room temperature. Amberlite IR-120 (H^+) ion-exchange resin was then added to the mixture, which was stirred for an additional 10 minutes until a pH of 5 to 6. The mixture was filtered and the solvent was removed under reduced pressure. The crude product was purified by column chromatography (9:1 CH_2Cl_2:MeOH) to afford **68b** as white crystals (142 mg, 54%). ^{1}H NMR (300 MHz, MeOD): δ 3.36-3.46 (m, 4H), 3.96 (dd, *J* = 11.9, 4.9 Hz, 1H), 3.89 (d, *J* = 11.9 Hz, 1H), 4.83 (d, *J* = 7.6 Hz, 1H), 7.04 (d, *J* = 9.0 Hz, 2H), 7.28- 7.43 (m, 7H). ^{13}C NMR (75 MHz, MeOD): δ 62.7, 67.0, 71.4, 74.8, 77.9, 78.0, 102.7, 118.1, 120.6, 129.0, 129.1, 129.5, 134.7, 138.2, 154.7, 154.9. LRMS (ESI): *m/z* calcd. for $C_{20}H_{22}NO_8$ $[M-H]^-$ 405.1, found 403.9.

4-Aminophenyl-β-D-glucopyranoside (68)

Compound **68b** (142 mg, 0.35 mmol) was dissolved in methanol (10 mL) in a 50 mL flame-dried round bottom flask. 20% palladium (II) hydroxide on carbon (25 mg, 0.0350 mmol) was added, the atmosphere was removed from the flask using a water aspirator, and a positive pressure of hydrogen gas was added. The contents were stirred overnight at room temperature, after which the mixture was filtered over a bed of celite and the solvent evaporated under reduced pressure to give **68** as light brown crystals (96 mg, 100%). ^{1}H NMR (300 MHz, D_2O): δ 3.42-3.61 (m, 4H), 3.72 (dd, *J* = 12.3, 5.3 Hz, 1H), 3.90 (dd, *J* = 12.4, 2.2 Hz, 1H), 4.95 (d, *J* = 7.6 Hz, 1H), 6.80 (d, *J* = 9.0 Hz, 2H), 6.98 (d, *J* = 8.9 Hz, 1H). ^{13}C NMR (75 MHz, D_2O): δ 61.2, 70.1, 73.6, 76.2, 76.6, 102.0, 118.2, 118.8, 142.3, 150.7. LRMS (ESI): *m/z* calcd. for $C_{12}H_{16}NO_6$ $[M-H]^-$ 271.1, found 270.0.

4-Acetamidophenyl- β-D-glucopyranoside (69)

Compound **68** (86 mg, 0.320 mmol) was dissolved in water (3 mL) in a 50 mL round bottom flask. Acetic anhydride (32 uL, 0.337 mmol) was added, and the solution was stirred overnight at room temperature. The reaction mixture was concentrated under reduced pressure, and the crude product was dissolved in a minimum of methanol and precipitated with ether to afford **69** as light brown crystals (73 mg, 74%). ^{1}H NMR (300 MHz, D_2O): δ 2.14 (s, 3H), 3.45-3.65 (m, 4H), 3.74 (dd, *J* = 12.4, 5.7 Hz, 1H), 3.92 (dd, *J* =

12.4, 2.2 Hz, 1H), 5.10 (d, *J* = 7.5 Hz, 1H), 7.12 (d, *J* = 9.0 Hz, 2H), 7.35 (d, *J* = 9.0 Hz, 2H). ^{13}C NMR (75 MHz, D_2O): δ 22.5, 60.4, 69.3, 72.8, 75.4, 76.0, 100.3, 117.0, 124.1, 131.8, 154.0, 173.0. LRMS (ESI): *m/z* calcd. for $C_{14}H_{18}NO_7$ $[M-H]^-$ 313.1, found 312.0.

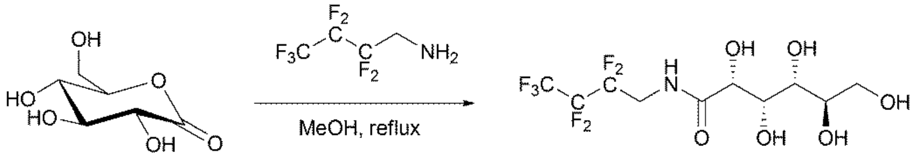

70

***N*-(2,2,3,3,4,4,4-Heptafluoro)butyl-D-gluconamide (70)**

To a solution of D-gluconic acid-*d*-lactone (0.89 g, 5.02 mmol) in MeOH (30 mL) was added 2,2,3,3,4,4,4-heptafluorobutylamine (0.69 mL, 5.02 mmol). The mixture was stirred under reflux for 48 hours. The solvent was evaporated and the residue was recrystallized in EtOH to afford **70** as a pale brown solid (21%). ^{1}H NMR (400 MHz, DMSO-*d*$_6$): δ 8.19 (t, *J* = 7.0 Hz, 1H), 5.56 (d, *J* = 5.4 Hz, 1H), 4.57 (d, *J* = 4.9 Hz, 1H), 4.51 (d, *J* = 5.8 Hz, 1H), 4.44 (d, *J* = 7.3 Hz, 1H), 4.36 (t, *J* = 5.8 Hz, 1H), 4.11 (dd, *J* = 4.9, 3.8 Hz, 1H), 4.06-3.86 (m, 3H), 3.59-3.55 (m, 2H), 3.45 (t, *J* = 2.6 Hz, 2H), 3.36-3.39 (m, 1H). ^{13}C NMR (100 MHz, DMSO-*d*$_6$): δ 173.7, 73.6, 72.2, 71.5, 70.1, 63.3. ^{19}F NMR (400 MHz, DMSO-*d*$_6$): δ -80.2, -117.7, -127.6. LRMS (ESI): *m/z* calcd. for $C_{10}H_{14}NaNO_6F_7$ $[M + Na]^+$ 400.2; found 400.2.

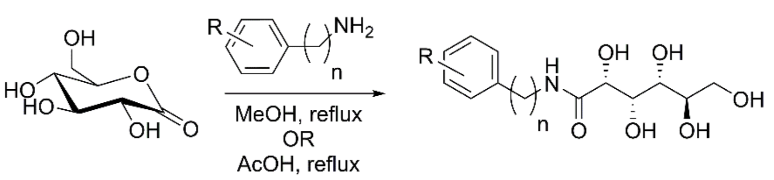

71: n = 0, R =H
72: n = 0, R = 3-F
73: n = 0, R = 4-F
74: n = 1, R = H
75: n = 1, R = 2-F
76: n = 1, R = 3-F
77: n = 1, R = 4-F
78: n = 2, R = H
79: n = 2, R = 2-F
80: n = 2, R = 3-F
81: n = 2, R = 4-F
82: n = 1, R = 2,5-difluoro
83: n = 0, R = 4-Br
84: n = 0, R = 2-F
85: n = 0, R = 3,5-difluoro
86: n = 0, R = 3-Br
87: n = 1, R = 2,6-difluoro
88: n = 0, R = 4-OMe
89: n = 1, R = 4-OMe
90: n = 1, R = 4-Me
91: n = 1, R = 2-OMe
92: n = 0, R = 2-OMe

***N*-Phenyl-D-gluconamide (71)**

To a solution of D-gluconic acid-*d*-lactone (0.50 g, 2.81 mmol) in MeOH (10 mL) was added aniline (0.26 mL, 2.81 mmol). The mixture was stirred under reflux for 48 hours. The solvent was evaporated and the residue was recrystallized in EtOH to afford **71** as a pale brown solid (87%). Characterization data is consistent with that of previously reported data.[38] ^{1}H NMR (400 MHz, DMSO-*d*$_6$): δ 8.68 (s, 1H), 6.86 (d, *J* = 8.1 Hz, 2H), 6.56 (t, *J* = 8.1 Hz, 2H), 6.21 (t, *J* = 8.1 Hz, 1H), 4.86 (d, *J* = 5.3 Hz, 1H), 3.76 (d, *J* = 5.2 Hz, 1H), 3.70-3.72 (m, 2H), 3.53 (t, *J* = 5.9 Hz, 1H), 5.33 (t, *J* = 4.8 Hz, 1H), 3.16-3.19 (m, 1H), 2.73-2.78 (m, 1H), 2.68 (t, *J* = 2.7 Hz, 2H), 2.52-2.58 (m, 1H). ^{13}C NMR (100 MHz, DMSO-*d*$_6$): δ 171.5, 138.5, 128.6, 123.4, 119.5, 74.2, 72.3, 71.5, 70.3, 63.3. LRMS (ESI): *m/z* calcd. for $C_{12}H_{18}NO_6$ $[M + H]^+$ 272.3, found 272.2.

***N*-3-Fluorophenyl-D-gluconamide (72)**

To a solution of D-gluconic acid-*d*-lactone (0.20 g, 1.12 mmol) in acetic acid (10 mL) was added 3-fluoroaniline (0.36 mL, 3.36 mmol). The mixture was stirred under reflux for 3 hours after which crude product was precipitated with hexanes and filtered to collect a dark brown solid. Recrystallized in EtOH afforded **72** as an off-white powder (62%). ^{1}H NMR (400 MHz, DMSO-*d*$_6$): δ 9.77 (s, 1H), 7.72 (dt, *J* = 11.9, 2.3 Hz, 1H), 7.51 (d, *J* = 8.4 Hz, 1H), 7.30-7.36 (m, 1H), 6.85-6.90 (m, 1H), 5.75 (d, *J* = 5.3 Hz, 1H), 4.60 (d, *J* = 5.0 Hz, 1H), 4.55-4.57 (m, 2H), 4.37 (t, *J* = 5.6 Hz, 1H), 4.18 (t, *J* = 4.6 Hz, 1H), 4.00-4.02 (m, 1H), 3.56-3.61 (m, 1H), 3.50-3.52 (m, 2H), 3.36-3.42 (m, 1H). ^{13}C NMR (100 MHz, DMSO-*d*$_6$): δ 172.0, 162.0 (d, $J_{C,F}$ = 242.4 Hz), 140.4 (d, $J_{C,F}$ = 2.9 Hz), 130.2 (d, $J_{C,F}$ = 9.5 Hz), 115.4 (d, $J_{C,F}$ = 8.2 Hz), 109.8 (d, $J_{C,F}$ = 20.6 Hz), 106.3 (d, $J_{C,F}$ = 26.1 Hz), 74.2, 72.2, 71.5, 70.3, 63.3. ^{19}F NMR (300 MHz, DMSO-*d*$_6$): δ -112.4. LRMS (ESI): *m/z* calcd. for $C_{12}H_{15}FNO_6$ $[M-H]^-$ 288.1, found 288.1.

***N*-4-Fluorophenyl-D-gluconamide (73)**

To a solution of D-gluconic acid-*d*-lactone (0.20 g, 1.12 mmol) in MeOH (10 mL) was added 4-fluoroaniline (0.11 mL, 1.12 mmol). The mixture was stirred under reflux for 48 hours. The solvent was evaporated and the residue was recrystallized in EtOH to afford **73** as a white powder (30%). ^{1}H NMR (400 MHz, DMSO-d_6): δ 8.80 (s, 1H), 6.90 (q, *J* = 5.5 Hz, 2H), 6.30 (t, *J* = 9.2 Hz, 2H), 4.86 (d, *J* = 5.0 Hz, 1H), 3.76 (d, *J* = 5.4, 1H), 3.69-3.72 (m, 2H), 3.53 (t, *J* = 6.0 Hz, 1H), 3.33 (t, *J* = 5.0 Hz, 1H), 3.16-3.19 (m, 1H), 2.73-2.77 (m, 1H), 2.67-2.69 (m, 2H), 2.53-2.58 (m, 1H). ^{13}C NMR (100 MHz, DMSO-d_6): δ 171.5, 158.1 (d, $J_{C,F}$ = 239.9 Hz), 135.0 (d, $J_{C,F}$ = 2.2 Hz), 121.4 (d, $J_{C,F}$ = 7.7 Hz), 115.1 (d, $J_{C,F}$ = 22.0 Hz), 74.2, 72.2, 71.5, 70.3, 63.3.^{19}F NMR (300 MHz, DMSO-d_6): δ -119.4. LRMS (ESI): *m/z* calcd. for $C_{12}H_{16}FNNaO_6$ [M+Na]$^+$ 312.3; found 312.2.

***N*-Benzyl-D-gluconamide (74)**

To a solution of D-gluconic acid-*d*-lactone (0.89 g, 5.02 mmol) in MeOH (30 mL) was added benzylamine (0.69 mL, 5.02 mmol). The mixture was stirred under reflux for 48 hours. The solvent was evaporated and the residue was recrystallized in EtOH to afford **74** as white crystals (97%). Characterization data is consistent with that of previously reported data.[38] ^{1}H NMR (400 MHz, DMSO-d_6): δ 8.17 (t, *J* = 6.4 Hz, 1H), 7.21-7.3 (m, 5H), 5.46 (d, *J* = 5.1 Hz, 1H), 4.58 (d, *J* = 4.8 Hz, 1H), 4.52 (d, *J* = 5.4, 1H), 4.47, (d, *J* = 7.7 Hz, 1H), 4.36 (t, *J* = 5.8 Hz, 1H), 4.31 (d, *J* = 6.5 Hz, 2H), 4.07 (t, *J* = 4.5 Hz, 1H), 3.96-3.98 (m, 1H), 3.56-3.58 (m, 1H), 3.51 (s, 2H), 3.36-3.39 (m, 1H). ^{13}C NMR (100 MHz, DMSO-d_6): δ 172.6, 139.6, 128.1, 127.1, 126.6, 73.7, 72.5, 71.6, 70.1, 63.4, 41.8. LRMS (ESI): *m/z* calcd. for $C_{13}H_{20}NO_6$ [M+H]$^+$ 286.1, found 286.1.

***N*-2-Fluorobenzyl-D-gluconamide (75)**

To a solution of D-gluconic acid-*d*-lactone (0.20 g, 1.12 mmol) in MeOH (10 mL) was added 2-fluorobenzylamine (0.13 mL, 1.12 mmol). The mixture was stirred under reflux for 48 hours. The solvent was evaporated and the residue was recrystallized in EtOH to afford **75** as white crystals (7%). ^{1}H NMR (400 MHz, DMSO-d_6): δ 7.34 (t, *J* = 6.1 Hz, 1H), 6.54 (t, *J* = 7.0 Hz, 1H), 7.29-7.25 (m, 1H), 7.17-7.11 (m, 2H), 5.51 (d, *J* = 5.3 Hz, 1H), 4.59 (d, *J* = 4.3 Hz, 1H), 4.54 (d, *J* = 4.6 Hz, 1H), 4.50 (d, *J* = 7.3 Hz, 1H), 4.38-4.34 (m, 3H), 4.09 (t, *J* = 4.3 Hz, 1H), 3.97-3.96 (m, 1H), 3.60-3.57 (m, 1H), 3.45 (s, 2H), 3.40-3.37 (m, 1H). ^{13}C NMR (100 MHz, DMSO-d_6): δ 172.9, 159.9 (d, $J_{C,F}$ = 244.0 Hz), 129.1 (d, $J_{C,F}$ = 4.8 Hz), 128.5 (d, $J_{C,F}$ = 8.2 Hz), 126.2 (d, $J_{C,F}$ = 15.1 Hz), 124.2 (d, $J_{C,F}$ = 3.3 Hz), 114.8 (d, $J_{C,F}$ = 21.4 Hz), 73.9, 72.5, 71.6, 70.1, 63.3, 35.6 (d, $J_{C,F}$ = 5.1 Hz). ^{19}F NMR (300 MHz, DMSO-d_6): δ -119.4. LRMS (ESI): *m/z* calcd. for $C_{13}H_{19}FNO_6$ [M+H]$^+$ 304.3; found 304.2.

***N*-3-Fluorobenzyl-D-gluconamide (76)**

To a solution of D-gluconic acid-*d*-lactone (0.20 g, 1.12 mmol) in MeOH (10 mL) was added 3-fluorobenzylamine (0.13 mL, 1.12 mmol). The mixture was stirred under reflux for 48 hours. The solvent was evaporated and the residue was recrystallized in EtOH to afford **76** as white crystals (62%). ^{1}H NMR (400 MHz, DMSO-d_6): δ 7.35 (t, *J* = 6.6 Hz, 1H), 6.48 (q, *J* = 8.0 Hz, 1H), 6.27 (m, 2H), 6.19 (m, 1H), 4.72 (d, *J* = 5.2 Hz, 1H), 3.74, (d, *J* = 5.2 Hz, 1H), 3.69 (d, *J* = 5.6 Hz, 1H), 3.64 (d, *J* = 7.3 Hz, 1H), 3.42-3.54 (m, 3H), 3.24 (t, *J* = 3.8 Hz, 1H), 3.12-3.14 (m, 1H), 2.72-2.76 (m, 1H), 2.66 (t, *J* = 2.7 Hz, 2H), 2.53-2.56 (m, 1H). ^{13}C NMR (100 MHz, DMSO-d_6): δ 172.8, 162.3 (d, $J_{C,F}$ = 246.2 Hz), 142.8 (d, $J_{C,F}$ = 7.4 Hz), 130.0 (d, $J_{C,F}$ = 8.1 Hz), 123.0 (d, $J_{C,F}$ = 2.3 Hz), 113.7 (d, $J_{C,F}$ = 21.3 Hz), 113.2 (d, $J_{C,F}$ = 21.4 Hz), 73.9, 72.6, 71.6, 70.1, 63.3, 41.3. ^{19}F NMR (300 MHz, DMSO-d_6): δ -113.7. LRMS (ESI): *m/z* calcd. for $C_{13}H_{18}FNNaO_6$ [M+Na]$^+$ 326.3; found 326.2.

***N*-4-Fluorobenzyl-D-gluconamide (77)**

To a solution of D-gluconic acid-*d*-lactone (0.20 g, 1.12 mmol) in MeOH (10 mL) was added 3-fluorobenzylamine (0.13 mL, 1.12 mmol). The mixture was stirred under reflux for 48 hours. The solvent was evaporated and the residue was recrystallized in EtOH to afford **77** as white crystals (62%). ^{1}H NMR (400 MHz, DMSO-d_6): δ 8.22 (t, *J* = 6.5 Hz, 1H), 7.31 (t, *J* = 7.1 Hz, 2H), 7.11, (t, *J* = 9.6 Hz, 2H), 5.46

(d, J = 5.0 Hz, 1H), 4.57 (d, J = 5.2 Hz, 1H), 4.52 (d, J = 5.6, 1H), 4.46 (d, J = 7.3, 1H), 4.36 (t, J = 5.6, 1H), 4.28 (d, J = 6.0, 2H), 4.06 (t, J = 4.7, 1H), 3.97-3.95 (m, 1H), 3.60-3.56 (m, 1H), 3.49 (t, J = 2.5, 2H), 3.40-3.36 (m, 1H). ^{13}C NMR (100 MHz, DMSO-d_6): δ 172.6, 161.1 (d, $J_{C,F}$ = 241.7 Hz), 135.8 (d, $J_{C,F}$ = 2.5 Hz), 129.1 (d, $J_{C,F}$ = 8.1 Hz), 114.8 (d, $J_{C,F}$ = 21.4 Hz), 73.9, 72.5, 71.5, 70.1, 63.4, 41.1. ^{19}F NMR (300 MHz, DMSO-d_6): δ -116.6. LRMS (ESI): *m/z* calcd. for $C_{13}H_{19}FNO_6$ $[M+H]^+$ 304.3; found 304.2.

***N*-2-Phenylethyl-D-gluconamide (78)**
To a solution of D-gluconic acid-*d*-lactone (0.20 g, 1.12 mmol) in MeOH (10 mL) was added phenethylamine hydrochloride (0.18 g, 1.12 mmol). Triethylamine (0.16 mL, 1.12 mmol) was added and the mixture was stirred under reflux for 48 hours. The solvent was evaporated and the residue was recrystallized in EtOH to afford **78** as white crystals (50%). Characterization data is consistent with that of previously reported data.[38] ^{1}H NMR (400 MHz, DMSO-d_6): δ 7.70 (t, J = 6.2 Hz, 1H), 7.18-7.31 (m, 5H), 5.40 (d, J = 5.2 Hz, 1H), 4.56 (d, J = 5.1 Hz, 1H), 4.50 (d, J = 5.9 Hz, 1H), 4.30 (d, J = 7.3 Hz, 1H), 4.35 (t, J = 6.1 Hz, 1H), 3.98 (t, J = 4.0 Hz, 1H), 3.90-3.93 (m, 1H), 3.55-3.59 (m, 1H), 3.47-3.48 (m, 2H), 3.38 (t, J = 6.0 Hz, 1H), 3.27-3.32 (m, 2H), 2.73 (t, J = 7.7 Hz, 2H). ^{13}C NMR (100 MHz, DMSO-d_6): δ 172.4, 139.5, 128.6, 128.4, 126.1, 73.6, 72.4, 71.5, 70.1, 63.4, 54.9, 35.2. LRMS (ESI): *m/z* calcd. for $C_{14}H_{20}NO_6$ $[M-H]^-$ 298.1, found 298.1.

***N*-2-(2-Fluoro)phenylethyl-D-gluconamide (79)**
To a solution of D-gluconic acid-*d*-lactone (0.20 g, 1.12 mmol) in MeOH (10 mL) was added 2-fluorophenethylamine (0.15 mL, 1.12 mmol). The mixture was stirred under reflux for 24 hours. The solvent was evaporated and the residue was recrystallized in EtOH to afford **79** as a white precipitate (51%). ^{1}H NMR (500 MHz, DMSO-d_6): δ 7.80 (t, J = 6.0 Hz, 1H), 7.28 (m, 2H), 7.14 (m, 2H), 5.39 (d, J = 6.0 Hz, 1H), 4.56 (d, J = 4.8 Hz, 1H), 4.49 (d, J = 5.1 Hz, 1H), 4.43 (d, J = 7.2 Hz, 1H), 4.35 (t, J = 5.2 Hz, 1H), 3.97 (t, J = 4.2 Hz, 1H), 3.90 (m, 1H), 3.57 (m, 1H), 3.47 (m, 2H), 3.37 (m, 1H), 3.30 (m, 2H), 2.76 (m, 2H). ^{13}C NMR (100 MHz, DMSO-d_6): δ 172.6, 160.7 (d, $J_{C,F}$ = 244.9 Hz), 131.2 (d, $J_{C,F}$ = 5.1 Hz), 128.3 (d, $J_{C,F}$ = 8.4 Hz), 126.1 (d, $J_{C,F}$ = 15.5 Hz), 124.4 (d, $J_{C,F}$ = 2.9 Hz), 115.1 (d, $J_{C,F}$ = 21.7), 73.6, 72.4, 71.5, 70.1, 63.4, 38.5, 28.5. ^{19}F NMR (300 MHz, DMSO-d_6): δ -118.80. LRMS (ESI): *m/z* calcd. for $C_{14}H_{21}FNO_6$ $[M+H]^+$ 318.1, found 318.2.

***N*-2-(3-Fluoro)phenylethyl-D-gluconamide (80)**
To a solution of D-gluconic acid-*d*-lactone (0.20 g, 1.12 mmol) in MeOH (10 mL) was added 3-fluorophenethylamine (0.15 mL, 1.12 mmol). The mixture was stirred under reflux for 24 hours. The solvent was evaporated and the residue was recrystallized in EtOH to afford **80** as a white precipitate (52%). ^{1}H NMR (400 MHz, DMSO-d_6): δ 7.72 (t, J = 5.9 Hz, 1H), 7.32 (m, 1H), 7.05 (m, 3H), 5.39 (d, J = 5.2 Hz, 1H), 4.55 (d, J = 5.2 Hz, 1H), 4.49 (d, J = 5.6 Hz, 1H), 4.42 (d, J = 7.4 Hz, 1H), 4.35 (t, J = 6.0 Hz, 1H), 3.98 (t, J = 4.0 Hz, 1H), 3.91 (m, 1H), 3.57 (m, 1H), 3.45 (m, 2H), 3.38 (m, 1H), 3.31 (m, 2H), 2.75 (t, J = 7.2 Hz, 2H). ^{13}C NMR (100 MHz, DMSO-d_6): δ 172.5, 162.2 (d, $J_{C,F}$ = 245.7 Hz), 142.5 (d, $J_{C,F}$ = 7.5 Hz), 130.2 (d, $J_{C,F}$ = 8.4 Hz), 124.8 (d, $J_{C,F}$ = 2.2 Hz), 115.3 (d, $J_{C,F}$ = 21.1 Hz), 112.9 (d, $J_{C,F}$ = 20.8 Hz), 73.6, 72.4, 71.5, 70.1, 63.4, 34.8. ^{19}F NMR (300 MHz, DMSO-d_6): δ -117.6. LRMS (ESI): *m/z* calcd. for $C_{14}H_{21}FNO_6$ $[M+H]^+$ 318.1, found 318.2.

***N*-2-(4-Fluoro)phenylethyl-D-gluconamide (81)**
To a solution of D-gluconic acid-*d*-lactone (0.20 g, 1.12 mmol) in MeOH (10 mL) was added 4-fluorophenethylamine (0.15 mL, 1.12 mmol). The mixture was stirred under reflux for 24 hours. The solvent was evaporated and the residue was recrystallized in EtOH to afford **81** as a white precipitate (97%). ^{1}H NMR (500 MHz, DMSO-d_6): δ 7.70 (t, J = 6.0 Hz, 1H), 7.25 (m, 2H), 7.10 (t, J = 9.0 Hz, 2H), 5.39 (d, J = 5.1 Hz, 1H), 4.56 (d, J = 5.2 Hz, 1H), 4.48 (d, J = 5.7 Hz, 1H), 4.42 (d, J = 7.2 Hz, 1H), 4.35 (t, J = 5.7 Hz, 1H), 3.97 (t, J = 3.9 Hz, 1H), 3.90 (m, 1H), 3.57 (m, 1H), 3.45 (m, 2H), 3.38 (m, 1H), 3.30 (m, 2H), 2.72 (t, J = 7.4 Hz, 2H). ^{13}C NMR (100 MHz, DMSO-d_6): δ 172.4, 160.8 (d, $J_{C,F}$ = 242.0 Hz), 135.6 (d, $J_{C,F}$ = 2.7 Hz), 130.4 (d, $J_{C,F}$ = 7.8 Hz), 115.0 (d, $J_{C,F}$ = 21.0 Hz), 73.6, 72.3, 71.5, 70.1, 63.4,

34.3. ^{19}F NMR (300 MHz, DMSO-*d*$_6$): δ -121.2. LRMS (ESI): *m/z* calcd. for $C_{14}H_{21}FNO_6$ $[M+H]^+$ 318.1, found 318.2.

***N*-2,5-Difluorobenzyl-D-gluconamide (82)**

To a solution of D-gluconic acid-*d*-lactone (0.20 g, 1.12 mmol) in MeOH (10 mL) was added 2,5-difluorobenzylamine (0.15 mL, 1.12 mmol). The mixture was stirred under reflux for 24 hours. The solvent was evaporated and the residue was recrystallized in EtOH to afford **82** as white crystals (79%). ^{1}H NMR (500 MHz, DMSO-*d*$_6$): δ 8.30 (t, *J* = 6.1 Hz, 1H), 7.18-7.23 (m, 2H), 7.08-7.12 (m, 1H), 5.59 (d, *J* = 5.0 Hz, 1H), 4.61 (d, *J* = 5.0 Hz, 1H), 4.57 (d, *J* = 5.4 Hz, 1H), 4.53 (d, *J* = 7.2 Hz, 1H), 4.35-4.39 (m, 2H), 4.27-4.31 (m, 1H), 4.11 (t, *J* = 4.0 Hz, 1H), 3.97-3.98 (m, 1H), 3.57-3.60 (m, 1H), 3.51 (s, 2H), 3.38-3.40 (m, 1H). ^{13}C NMR (100 MHz, DMSO-*d*$_6$): δ 173.1, 158.3 (d, $J_{C,F}$ = 240.4 Hz), 155.8 (d, $J_{C,F}$ = 240.4 Hz), 128.7 (dd, $J_{C,F}$ = 17.5 Hz, 7.8 Hz), 116.3 (dd, $J_{C,F}$ = 24.3 Hz, 8.9 Hz), 115.3 (dd, $J_{C,F}$ = 25.3 Hz, 4.8 Hz), 114.6 (dd, $J_{C,F}$ = 24.2 Hz, 8.4 Hz), 74.0, 72.6, 71.6, 70.2, 63.3, 35.5 (d, $J_{C,F}$ = 4.0 Hz). ^{19}F NMR (300 MHz, DMSO-*d*$_6$): δ -122.7 (d, $J_{F,F}$ = 18.2 Hz), -128.8 (d, $J_{F,F}$ = 18.3 Hz). LRMS (ESI): *m/z* calcd. for $C_{13}H_{18}$ F_2NO_6 $[M+H]^+$ 322.1, found 322.2.

***N*-4-Bromophenyl-D-gluconamide (83)**

To a solution of D-gluconic acid-*d*-lactone (0.20 g, 1.12 mmol) in MeOH (10 mL) was added 4-bromoaniline (0.15 mL, 1.12 mmol). The mixture was stirred under reflux for 24 hours. The solvent was evaporated and the residue was recrystallized in EtOH to afford **83** as a white powder (22%). Characterization data is consistent with that previously published.[39] ^{1}H NMR (500 MHz, DMSO-*d*$_6$): δ 9.71 (s, 1H), 7.72 (d, *J* = 8.7 Hz, 2H), 7.48 (d, *J* = 8.7 Hz, 2H), 5.74 (d, *J* = 5.0 Hz, 1H), 4.61 (br. s, 1H), 4.56 (d, *J* = 7.1 Hz, 2H), 4.37 (t, *J* = 5.6 Hz, 1H), 4.17 (t, *J* = 4.2 Hz, 1H), 4.00 (br. s, 1H), 3.58 (m, 1H), 3.50 (br. s, 1H), 3.39 (m, 1H). ^{13}C NMR (100 MHz, DMSO-*d*$_6$): δ 171.8, 138.0, 131.3, 121.6, 115.0, 74.2, 72.2, 71.5, 70.3, 63.3. LRMS (ESI): *m/z* calcd. for $C_{27}H_{39}N_2O_{13}Br_2$ $[2M + C_3H_7O]^+$ 759.4, found 759.1.

***N*-2-Fluorophenyl-D-gluconamide (84)**

To a solution of D-gluconic acid-*d*-lactone (0.50 g, 2.81 mmol) in acetic acid (5 mL) was added 2-fluoroaniline (0.81 mL, 8.42 mmol). The mixture was stirred under reflux for 1 hour. The crude product was precipitated with hexanes and filtered to obtain a dark brown sludge. The solid was recrystallized in EtOH to afford **84** as a white powder (33%). ^{1}H NMR (400 MHz, DMSO-*d*$_6$): δ 9.21 (br. s, 1H), 8.13 (td, *J* = 7.9, 2.1 Hz, 1H), 7.31-7.26 (m, 1H), 7.20-712 (m, 2H), 5.95 (d, *J* = 6.0 Hz, 1H), 4.67 (d, *J* = 6.9 Hz, 1H), 4.62 (dd, *J* = 8.4, 5.4 Hz, 2H), 4.38 (t, *J* = 5.7 Hz, 1H), 4.24 (dd, *J* = 4.9, 3.4 Hz, 1H), 4.04-4.01 (m, 1H), 3.62-3.49 (m, 3H), 3.42-3.33 (m, 1H). ^{13}C NMR (100 MHz, DMSO-*d*$_6$): δ 171.5, 152.5 (d, $J_{C,F}$ = 241.6 Hz), 125.9 (d, $J_{C,F}$ = 10.7 Hz), 124.7 (d, $J_{C,F}$ = 7.3 Hz), 124.5 (d, $J_{C,F}$ = 3.4 Hz), 122.0, 115.2 (d, $J_{C,F}$ = 18.8 Hz), 73.9, 72.2, 71.6, 70.2, 63.3. ^{19}F NMR (300 MHz, DMSO-*d*$_6$): δ -128.8. LRMS (ESI): *m/z* calcd. for $C_{12}H_{15}FNO_6$ $[M-H]^-$ 288.1, found 288.1.

***N*-3,5-Difluorophenyl-D-gluconamide (85)**

To a solution of D-gluconic acid-*d*-lactone (0.23 g, 1.29 mmol) in acetic acid (5 mL) was added 3,5-difluoroaniline (0.5 g, 3.87 mmol). The mixture was stirred under reflux for 1 hour. The crude product was precipitated with hexanes and filtered to obtain a dark brown sludge. The solid was recrystallized in EtOH to afford **85** as a white powder (22%). ^{1}H NMR (400 MHz, DMSO-*d*$_6$): δ 9.98 (s, 1H), 7.55 (d, *J* = 9.5 Hz, 2H), 6.90 (t, *J* = 9.5 Hz, 1H), 5.82 (d, *J* = 4.6 Hz, 1H), 4.61-4.56 (m, 3H), 4.37 (t, *J* = 5.6 Hz, 1H), 4.19 (t, *J* = 3.8 Hz, 1H), 4.02-4.00 (m, 1H), 3.60-3.57 (m, 1H), 3.51 (br. s, 2H), 3.43-3.38 (m, 1H). ^{13}C NMR (100 MHz, DMSO-*d*$_6$): δ 172.5, 162.5 (d, $J_{C,F}$ = 245.0 Hz), 162.0 (d, $J_{C,F}$ =242.8 Hz), 141.2 (t, $J_{C,F}$ = 14.2 Hz), 102.5 (dd, $J_{C,F}$ = 21.6, 8.3 Hz), 98.4 (t, $J_{C,F}$ = 26.4), 74.2, 72.1, 71.5, 70.4, 63.3. ^{19}F NMR (300 MHz, DMSO-*d*$_6$): δ -109.7 (t, $J_{F,F}$ = 10.0 Hz). LRMS (ESI): *m/z* calcd. for $C_{12}H_{14}F_2NO_6$ $[M-H]^-$ 306.1, found 306.1.

***N*-3-Bromophenyl-D-gluconamide (86)**

To a solution of D-gluconic acid-*d*-lactone (0.20 g, 1.12 mmol) in MeOH (15 mL) was added 3-bromoaniline (0.15 mL, 1.12 mmol). The mixture was stirred under reflux for 48 hours. The solvent was evaporated and the residue was recrystallized in EtOH to afford **86** as white crystals (60%). ^{1}H NMR (300 MHz, DMSO-d_6): δ 9.75 (s, 1H), 8.09 (d, *J* = 1.9 Hz, 1H), 7.65-7.69 (m, 1H), 7.24-7.29 (m, 2H), 5.73 (d, *J* = 5.1 Hz, 1H), 4.59 (d, *J* = 5.2 Hz, 1H), 4.55 (d, *J* = 6.8 Hz, 2H), 4.36 (t, *J* = 5.5 Hz, 1H), 4.18 (dd, *J* = 5.0, 3.7 Hz, 1H), 3.98-4.02 (m, 1H), 3.56-3.62 (m, 1H), 3.50-3.52 (m, 2H), 3.37-3.42 (m, 1H). ^{13}C NMR (100 MHz, DMSO-d_6): δ 172.1, 140.2, 130.5, 125.9, 122.0, 121.4, 118.5, 74.2, 72.1, 71.5, 70.4, 63.3. LRMS (ESI): *m/z* calcd. for $C_{12}H_{16}BrKNO_6$ $[M+K]^+$ 390.0, found 390.0.

***N*-2,6-Difluorobenzyl-D-gluconamide (87)**

To a solution of D-gluconic acid-*d*-lactone (0.20 g, 1.12 mmol) in MeOH (15 mL) was added 2,6-difluoroaniline (0.13 mL, 1.12 mmol). The mixture was stirred under reflux for 24 hours. The solvent was evaporated and the residue was recrystallized in EtOH to afford **87** as white crystals (89%). ^{1}H NMR (300 MHz, DMSO-d_6): δ 7.89 (t, *J* = 5.7 Hz, 1H), 7.33-7.43 (m, 1H), 7.07 (t, *J* = 8.1 Hz, 2H), 5.35 (d, *J* = 5.3 Hz, 1H), 4.53 (d, *J* = 5.0 Hz, 1H), 4.38-4.48 (m, 3H), 4.27-4.34 (m, 1H), 4.00 (dd, *J* = 5.2, 3.8 Hz, 1H), 3.87-3.91 (m, 1H), 3.53-3.59 (m, 1H), 3.44-3.45 (m, 2H), 3.35-3.39 (m, 1H). ^{13}C NMR (100 MHz, DMSO-d_6): δ 172.2, 162.2 (d, $J_{C,F}$ = 8.4 Hz), 159.8 (d, $J_{C,F}$ = 8.2 Hz), 129.8 (t, $J_{C,F}$ = 10.4 Hz), 114.2 (t, $J_{C,F}$ = 19.6 Hz), 111.5 (dd, $J_{C,F}$ = 18.9, 6.5 Hz), 73.5, 72.3, 71.5, 70.1, 63.4. ^{19}F NMR (300 MHz, DMSO-d_6): δ -114.5. LRMS (ESI): *m/z* calcd. for $C_{13}H_{17}F_2KNO_6$ $[M+K]^+$ 360.1, found 360.1.

***N*-4-Methoxyphenyl-D-gluconamide (88)**

To a solution of D-gluconic acid-*d*-lactone (0.20 g, 1.12 mmol) in MeOH (15 mL) was added *p*-anisidine (0.14 g, 1.12 mmol). The mixture was stirred under reflux for 24 hours. The solvent was evaporated and the residue was recrystallized in EtOH to afford **88** as white crystals (21%). Characterization data is consistent with that previously published.[39] ^{1}H NMR (400 MHz, DMSO-d_6): δ 9.41 (s, 1H), 7.61 (d, *J* = 9.3 Hz, 2H), 6.86 (d, *J* = 9.0 Hz, 2H), 5.65 (d, *J* = 5.3 Hz, 1H), 4.58 (d, *J* = 5.1 Hz, 1H), 4.53-4.51 (m, 2H), 4.36 (t, *J* = 5.9 Hz, 1H), 4.14 (dd, *J* = 4.1, 5.2 Hz, 1H), 4.01-3.99 (m, 1H), 3.72 (s, 3H), 3.61-3.56 (m, 1H), 3.51-3.49 (m, 2H), 3.41-3.36 (m, 1H). ^{13}C NMR (100 MHz, DMSO-d_6): δ 171.0, 155.3, 131.7, 121.0, 113.7, 74.1, 72.3, 71.5, 70.3, 63.3, 55.1. LRMS (ESI): *m/z* calcd. for $C_{13}H_{18}NO_7$ $[M-H]^-$ 300.1, found 300.1.

***N*-4-Methoxybenzyl-D-gluconamide (89)**

To a solution of D-gluconic acid-*d*-lactone (0.20 g, 1.12 mmol) in MeOH (15 mL) was added 4-methoxybenzylamine (0.15 mL, 1.12 mmol). The mixture was stirred under reflux for 24 hours. The solvent was evaporated and the residue was recrystallized in EtOH to afford **89** as white crystals (62%). ^{1}H NMR (400 MHz, DMSO-d_6): δ 8.06 (t, *J* = 6.2 Hz, 1H), 7.20 (d, *J* = 8.7 Hz, 2H), 6.85 (d, *J* = 8.8 Hz, 2H), 5.43 (d, *J* = 4.4 Hz, 1H), 4.57 (br.s, 1H), 4.51 (br.s, 1H), 4.44 (d, *J* = 7.4 Hz, 1H), 4.35 (t, *J* = 5.4 Hz, 1H), 4.23 (d, *J* = 6.8 Hz, 2H), 4.04 (t, *J* = 3.4 Hz, 1H), 3.96-3.94 (m, 1H), 3.71 (s, 3H), 3.60-3.56 (m, 1H), 3.49 (br. s, 2H), 3.40-3.36 (m, 1H). ^{13}C NMR (100 MHz, DMSO-d_6): δ 172.4, 158.1, 131.5, 128.5, 113.5, 73.8, 72.5, 71.5, 70.1, 63.4, 55.0, 41.2. LRMS (ESI): *m/z* calcd. for $C_{14}H_{20}NO_7$ $[M-H]^-$ 314.1, found 314.1.

***N*-4-Methylbenzyl-D-gluconamide (90)**

To a solution of D-gluconic acid-*d*-lactone (0.10 g, 0.56 mmol) in MeOH (10 mL) was added 4-methylbenzylamine (0.07 mL, 0.56 mmol). The mixture was stirred under reflux for 24 hours. The solvent was evaporated and the residue was recrystallized in EtOH to afford **90** as white crystals (88%). ^{1}H NMR (400 MHz; DMSO-d_6): δ 8.08 (t, *J* = 6.1 Hz, 1H), 7.17-7.08 (dd, 4H), 5.43 (d, *J* = 5.1 Hz, 1H), 4.53 (dd, *J* = 22.4, 5.4 Hz, 2H), 4.44 (d, *J* = 7.2 Hz, 1H), 4.34 (t, *J* = 5.7 Hz, 1H), 4.26 (d, *J* = 6.2 Hz, 2H), 4.05 (dd, *J* = 5.1, 3.7 Hz, 1H), 3.95 (m, 1H), 3.60-3.57 (m, 1H), 3.49 (m, 2H), 3.37 (dd, *J* = 11.0, 5.7 Hz, 1H), 2.26 (s, 3H). ^{13}C NMR (100 MHz; DMSO-d_6): δ 172.4, 136.5, 135.6, 128.7, 127.1, 73.8, 72.5, 71.5, 70.1, 63.4, 41.5, 30.7. LRMS (ESI): *m/z* calcd. for $C_{14}H_{20}NO_6$ $[M-H]^-$ 298.1, found 298.1.

***N*-2-Methoxybenzyl-D-gluconamide (91)**
To a solution of D-gluconic acid-*d*-lactone (0.10 g, 0.56 mmol) in MeOH (10 mL) was added 2-methoxybenzylamine (0.08 mL, 0.58 mmol). The mixture was stirred under reflux for 24 hours. The solvent was evaporated and the residue was recrystallized in EtOH to afford **91** as white crystals (51%). ^{1}H NMR (400 MHz, DMSO-d_6): δ 7.95 (t, J = 6.3 Hz, 1H), 7.20-7.24 (m, 2H), 6.95-6.97 (m, 1H), 6.86-6.89 (m, 1H), 5.50 (d, J = 4.7 Hz, 1H), 4.55 (dd, J = 18.5, 5.0 Hz, 2H), 4.48 (d, J = 7.2 Hz, 1H), 4.35 (t, J = 5.5 Hz, 1H), 4.27 (d, J = 6.2 Hz, 2H), 4.06-4.08 (m, 1H), 3.95-3.97 (m, 1H), 3.80 (s, 3H), 3.56-3.60 (m, 1H), 3.48-3.50 (m, 2H), 3.37-3.40 (m, 1H). ^{13}C NMR (100 MHz, DMSO-d_6): δ 172.5, 156.5, 127.8, 127.5, 126.7, 120.1, 110.2, 73.9, 72.5, 71.5, 70.1, 63.3, 55.3, 37.2. LRMS (ESI): *m/z* calcd. for $C_{14}H_{20}NO_7$ $[M-H]^-$ 314.1, found 314.1.

***N*-2-Methoxyphenyl-D-gluconamide (92)**
To a solution of D-gluconic acid-*d*-lactone (0.10 g, 0.56 mmol) in MeOH (10 mL) was added *o*-anisidine (0.07 g, 0.56 mmol). The mixture was stirred under reflux for 24 hours. The solvent was evaporated and the residue was recrystallized in EtOH to afford **92** as white crystals (65%). ^{1}H NMR (400 MHz; DMSO-d_6): δ 9.25 (s, 1H), 8.33 (d, J = 8.0 Hz, 1H), 7.07-7.05 (m, 2H), 6.95-6.90 (m, 1H), 6.03 (d, J = 4.6 Hz, 1H), 4.64-4.60 (m, 3H), 4.38 (t, J = 5.6 Hz, 1H), 4.20 (t, J = 3.4 Hz, 1H), 4.05-4.03 (m, 1H), 3.86 (s, 3H), 3.61-3.50 (m, 3H), 3.43-3.39 (m, 1H). ^{13}C NMR (100 MHz; DMSO-d_6): δ 170.9, 147.7, 127.1, 123.5, 120.5, 118.1, 110.8, 74.2, 72.5, 71.6, 70.1, 63.3, 55.8. LRMS (ESI): *m/z* calcd. for $C_{13}H_{19}NNaO_7$ $[M+Na]^+$ 324.1, found 324.0.

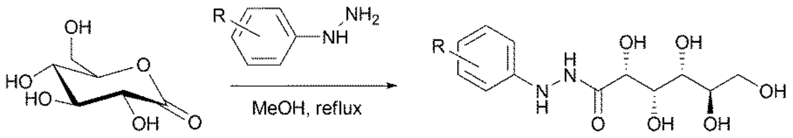

93: R = H
94: R = 2F

2-Phenylhydrazide-D-gluconic acid (93)
To a solution of D-gluconic acid-*d*-lactone (0.20 g, 1.12 mmol) in MeOH (15 mL) was added phenylhydrazine (0.11 mL, 1.12 mmol). The mixture was stirred under reflux for 24 hours. The solvent was evaporated and the residue was recrystallized in EtOH to afford **93** as white crystals (83%). ^{1}H NMR (400 MHz, DMSO-d_6): δ 9.45 (d, J = 2.9 Hz, 1H), 7.61 (d, J = 2.5 Hz, 1H), 7.10 (dd, J = 8.3, 7.5 Hz, 2H), 6.76 (d, J = 8.2 Hz, 2H), 6.68 (t, J = 7.0 Hz, 1H), 5.42 (d, J = 5.1 Hz, 1H), 4.57 (d, J = 4.5 Hz, 1H), 4.48 (t, J = 6.3 Hz, 2H), 4.36 (t, J = 5.6 Hz, 1H), 4.14 (t, J = 4.6 Hz, 1H), 3.94-3.95 (m, 1H), 3.57-3.62 (m, 1H), 3.45-3.52 (m, 2H), 3.36-3.40 (m, 1H).^{13}C NMR (100 MHz, DMSO-d_6): δ 172.1, 149.3, 129.0, 118.7, 112.8, 73.9, 72.5, 71.9, 70.8, 63.8. LRMS (ESI): *m/z* calcd. for $C_{12}H_{18}KN_2O_6$ $[M+K]^+$ 325.1, found 325.1.

2-(2-Fluoro)phenylhydrazide-D-gluconic acid (94)
To a solution of D-gluconic acid-*d*-lactone (0.20 g, 1.12 mmol) in MeOH (15 mL) was added 2-fluorophenylhydrazine hydrochloride (0.18 g, 1.12 mmol). Triethylamine (0.51 mL, 1.12 mmol) was added and the mixture was stirred under reflux for 24 hours. The solvent was evaporated and the residue was recrystallized in EtOH to afford **94** as white crystals (39%). ^{1}H NMR (400 MHz, DMSO-d_6): δ 9.55 (d, J = 2.3 Hz, 1H), 7.49 (br.s, 1H), 7.02-7.07 (m, 1H), 6.89-6.97 (m, 2H), 6.67-6.72 (m, 1H), 5.46 (d, J = 5.4 Hz, 1H), 4.59 (d, J = 4.8 Hz, 1H), 4.50-4.53 (m, 2H), 4.34-4.38 (m, 1H), 4.16 (dd, J = 5.1, 4.2 Hz, 1H), 3.94-3.97 (m, 1H), 3.57-3.62 (m, 1H), 3.49-3.52 (m, 2H), 3.37-3.41 (m, 1H). ^{13}C NMR (100 MHz, DMSO-d_6): δ 172.2, 150.2 (d, $J_{C,F}$ = 232.6 Hz), 136.8 (d, $J_{C,F}$ = 10.5 Hz), 124.4 (d, $J_{C,F}$ = 2.9 Hz), 118.5 (d, $J_{C,F}$ = 6.6 Hz), 114.5 (d, $J_{C,F}$ = 17.3 Hz), 114.1 (d, $J_{C,F}$ = 3.1 Hz), 73.6, 72.2, 71.5, 70.4, 63.4. ^{19}F NMR (300 MHz, DMSO-d_6): δ -133.4. LRMS (ESI): *m/z* calcd. for $C_{12}H_{17}FKN_2O_6$ $[M+K]^+$ 343.1, found 343.1.

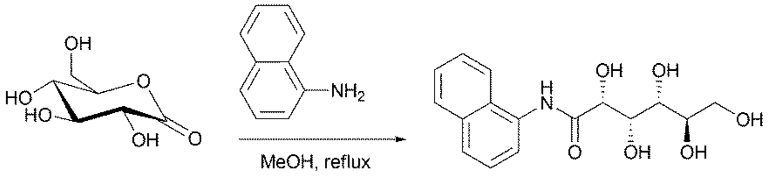

***N*-1-Naphthyl-D-gluconamide (95)**

To a solution of D-gluconic acid-*d*-lactone (0.20 g, 1.12 mmol) in MeOH (15 mL) was added 1-naphthylamine (0.16 g, 1.12 mmol). The mixture was stirred under reflux for 48 hours. The solvent was evaporated and the residue was recrystallized in EtOH to afford **95** as a white powder (65%). ^{1}H NMR (400 MHz, DMSO-d_6): δ 9.69 (br. s, 1H), 8.01-8.03 (m, 1H), 7.93-7.96 (m, 1H), 7.76-7.81 (m, 2H), 7.49-7.58 (m, 3H), 5.89 (d, *J* = 5.1 Hz, 1H), 4.74 (d, *J* = 7.2 Hz, 1H), 4.64 (dd, *J* = 5.1, 3.0 Hz, 2H), 4.39 (t, *J* = 5.5 Hz, 1H), 4.34 (dd, *J* = 4.8, 3.6 Hz, 1H), 4.10-4.13 (m, 1H), 3.54-3.66 (m, 3H), 3.40-3.46 (m, 1H). ^{13}C NMR (100 MHz, DMSO-d_6): δ 172.0, 133.7, 133.2, 128.2, 127.4, 126.0, 126.0, 125.6, 125.1, 122.2, 120.7, 74.4, 72.4, 71.6, 70.4, 63.4. LRMS (ESI): *m/z* calcd. for $C_{16}H_{19}NNaO_6$ $[M+Na]^+$ 344.1, found 344.1.

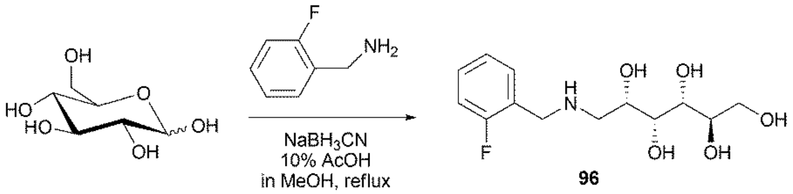

1-Deoxy-1-[(2-fluorophenylmethyl)amino]-D-glucitol (96)

To a solution of D-glucose (0.1 g, 0.56 mmol) and 2-fluorobenzylamine (0.45 mL, 3.9 mmol) in 10% acetic acid in methanol (15 mL), was added sodium cyanoborohydride (0.1 g, 1.67 mmol). The mixture was stirred under reflux for 3 hours. The solution was allowed to cool to room temperature and concentrated hydrochloric acid was added dropwise until the solution became slightly acidic. The solvent was evaporated and the product was purified by reverse phase column chromatography (0% - 50% ACN in H2O) to yield **96** (31%). ^{1}H NMR (500 MHz, DMSO-d_6): δ 9.20 (br.s, 1H), 7.69-7.73 (m, 1H), 7.45-7.50 (m, 1H), 7.25-7.30 (m, 2H), 5.48 (d, *J* = 4.7 Hz, 1H), 4.84-4.85 (m, 1H), 4.67 (d, *J* = 4.8 Hz, 1H), 4.61-4.62 (m, 1H), 4.47 (t, *J* = 5.7 Hz, 1H), 4.19 (br.s, 2H), 3.98-4.01 (m, 1H), 3.68-3.70 (m, 1H), 3.54-3.58 (m, 1H), 3.39-3.48 (m, 3H), 3.10-3.13 (m, 1H), 2.95-3.00 (m, 1H). ^{13}C NMR (100 MHz, DMSO-d_6): δ 133.0 (d, $J_{C,F}$ = 2.9 Hz), 132.5 (d, $J_{C,F}$ = 7.3 Hz), 125.5 (d, $J_{C,F}$ = 2.9 Hz), 116.4 (d, $J_{C,F}$ = 20.2 Hz), 71.7, 70.7, 70.6, 68.6, 63.6, 49.5, 44.3. LRMS (ESI): *m/z* calcd. for $C_{12}H_{18}FNNaO_5$ $[M+Na]^+$ 298.1, found 298.1.

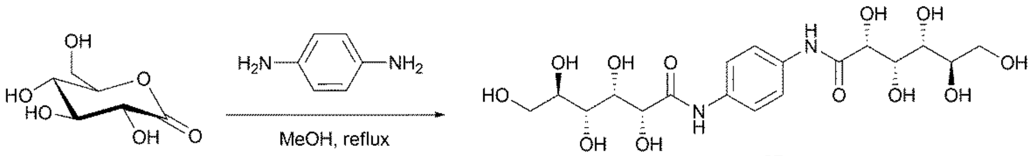

1,4-Bis-(D-gluconamide)benzene (97)

To a solution of D-gluconic acid-*d*-lactone (0.40 g, 2.25 mmol) in acetic acid (10 mL) was added *p*-phenylenediamine (0.12 g, 1.12 mmol). The mixture was stirred under reflux for 4 hours. The crude product was precipitated with hexanes and collected by suction filtration. The crude product was recrystallized in EtOH to afford **97** as a light brown powder (45%). ^{1}H NMR (400 MHz; DMSO-d_6): δ 9.47 (s, 2H), 7.63 (s, 4H), 5.67 (d, *J* = 5.3 Hz, 2H), 4.58 (d, *J* = 5.0 Hz, 2H), 4.52-4.55 (m, 4H), 4.35 (t, *J* = 5.7 Hz, 2H), 4.16 (dd, *J* = 5.0, 3.8 Hz, 2H), 3.99-4.02 (m, 2H), 3.57-3.61 (m, 2H), 3.49-3.52 (m, 4H),

3.36-3.42 (m, 2H). ^{13}C NMR (100 MHz; DMSO-d_6): δ 171.2, 134.1, 119.7, 74.2, 72.3, 71.5, 70.3, 63.3. LRMS (ESI): *m/z* calcd. for $C_{18}H_{27}N_2O_{12}$ [M-H]$^-$ 463.2, found 463.2.

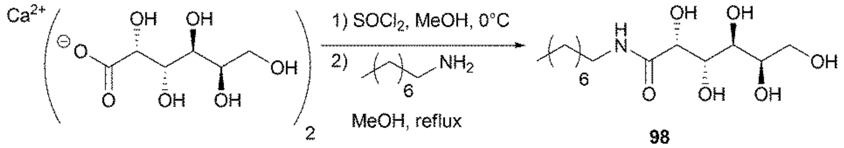

98

***N*-Octyl-D-galactonamide (98)**

A solution of calcium galactonate (200 mg, 0.46 mmol) in methanol (2 mL) was cooled to 0 °C and $SOCl_2$ (70 μL, 0.93 mmol) was added dropwise. The solution was slowly warmed to room temperature and was stirred overnight. The mixture was then evaporated and dried *in vacuo* to give 200 mg of a white powder. This powder was then dissolved in methanol (2 mL) and *n*-octylamine (230 μL, 1.44 mmol) was added. The mixture was refluxed for 2 hours then cooled in an ice bath. The precipitate was filtered off and washed with cold methanol to afford **98** as a white powder (112 mg, 38%). Characterization data is consistent with that previously reported in the literature.[40, 41] ^1H NMR (500 MHz, DMSO-d_6): δ 7.53, (t, *J* = 6.0 Hz, 1H), 5.07 (d, *J* = 7.2 Hz, 1H), 4.44 (t, *J* = 5.6 Hz, 1H), 4.28 (d, *J* = 8.1 Hz, 1H), 4.17 (d, *J* = 6.6 Hz, 1H), 4.12 (d, *J* = 7.5 Hz, 1H), 4.08 (d, *J* = 8.1 Hz, 1H), 3.78 (t, *J* = 8.7 Hz, 1H), 3.69 (q, *J* = 5.6 Hz, 1H), 3.45-3.37 (m, 3H), 3.07 (m, 2H), 1.40 (m, 2H), 1.31-1.18 (m, 10H), 0.86 (t, *J* = 6.6 Hz, 3H). ^{13}C NMR (125 MHz, DMSO-d_6): δ 173.3, 70.9, 70.7, 69.8, 69.1, 63.2, 38.3, 31.3, 29.3, 28.8, 28.7, 26.4, 22.1, 14.0. LRMS (ESI): *m/z* calcd. for $C_{14}H_{30}NaNO_6$ [M+Na]$^+$ 330.2; found 330.2.

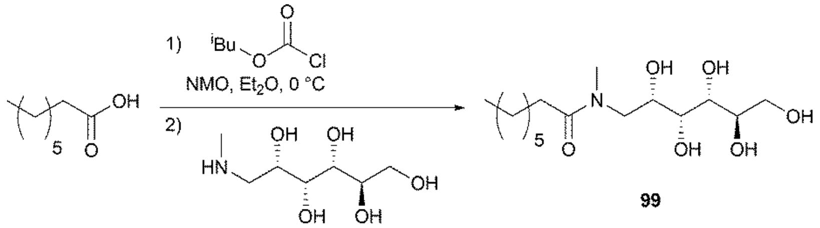

99

***N*-Octanoyl-*N*-methyl-D-glucamine (99)**

A solution of octanoic acid (0.89 mL, 5.63 mmol) in Et_2O (10 mL) was cooled to 0 ºC and isobutyl chloroformate (0.74 mL, 5.63 mmol) was added. After stirring for 5 min at 0 ºC, *N*-methylmorpholine (0.68 mL, 5.63 mmol) was added dropwise and the mixture was stirred for 10 min. The precipitate was removed by filtration trough Celite® and the filter cake washed with 10 mL of Et_2O. The solution of the crude mixed anhydride in Et_2O was then added by cannula to a cooled solution (0 ºC) of *N*-methyl-D-glucamine (500 mg, 2.56 mmol) in MeOH (10 mL). The mixture was warmed to room temperature, stirred for 1 hour and concentrated *in vacuo*. Recrystallization in MeOH afforded **99** (250 mg, 30%) as a white powder. ^1H NMR (500 MHz, DMSO-d_6, present as a 1.3:1 mixture of rotamers): δ 4.87 (d, *J* = 5.2 Hz, 1H, major), 4.72 (d, *J* = 5.1 Hz, 1H, minor), 4.51 (d, *J* = 5.4 Hz, 1H, major), 4.47 (d, *J* = 5.5 Hz, 1H, minor), 4.41-4.26 (m, 3H), 3.74 (m, 1H), 3.56 (m, 2H), 3.52-3.35 (m, 4H), 3.29 (dd, *J* = 14.4, 3.7 Hz, 1H, major), 3.21 (dd, *J* = 13.2, 8.1 Hz, 1H, minor), 2.99 (s, 3H, minor), 2.80 (s, 3H, major), 2.34 (m, 2H, major), 2.26 (t, *J* = 7.4 Hz, 2H, minor), 1.46 (m, 2H), 1.24 (m, 8H), 0.86 (t, *J* = 6.7 Hz, 3H). ^{13}C NMR (125 MHz, DMSO-d_6, present as a mixture of rotamers): δ (major) 172.4, 71.5, 71.5, 70.8, 69.9, 63.3, 51.9, 33.5, 32.2, 31.2, 28.9, 28.7, 24.9, 22.1, 14.0; δ (minor) 172.6, 72.5, 71.4, 71.3, 69.3, 63.3, 50.8, 36.7, 32.7, 31.2, 28.8, 28.7, 24.9, 22.1, 14.0. HRMS (ESI): *m/z* calcd. for $C_{15}H_{32}NO_6$ [M+H]$^+$ 322.223; found 322.285.

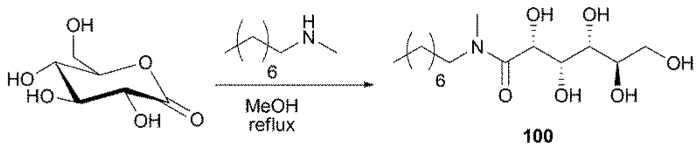

100

N-Methyl-N-octyl-D-gluconamide (100)
To a solution of D-gluconic acid-*d*-lactone (950 mg, 5.3 mmol) in MeOH (20 mL) was added *N*-methyl-*N*-octylamine (760 mg, 5.3 mmol). The mixture was stirred under reflux for 1 hour. The clear solution was cooled to room temperature and filtered successively through Dowex-1X8 resin (OH^-) followed by Amberlite IR-120 (H^+). The solvent was evaporated and the residue was dried *in vacuo* to afford **100** as a waxy solid (800 mg, 47%). ^{1}H NMR (500 MHz, DMSO-d_6, present as a 1.1:1 mixture of rotamers): δ 4.77 (d, *J* = 7.2 Hz, 1H, minor), 4.71 (d, *J* = 7.1 Hz, 1H, major), 4.50-4.25 (m, 5H), 3.82 (m, 1H), 3.57 (m, 1H), 3.47 (m, 1H), 3.423.20 (m, 4H), 2.99 (s, 3H, major), 2.80 (s, 3H, minor), 1.52 (quint, *J* = 6.7 Hz, 2H, minor), 1.43 (quint, *J* = 6.7 Hz, 2H, major), 1.25 (m, 10H), 0.86 (t, *J* = 6.7 Hz, 3H). ^{13}C NMR (125 MHz, DMSO-d_6, present as a mixture of rotamers): δ (major) 171.5, 71.6, 71.5, 70.4, 69.5, 63.4, 47.2, 34.4, 31.2, 28.8, 28.7, 26.4, 26.2, 22.1, 14.0; δ (minor) 171.6, 71.7, 71.1, 70.9, 69.2, 63.4, 48.5, 33.1, 31.2, 28.8, 28.7, 26.4, 26.2, 22.1, 14.0. HRMS (ESI): *m/z* calcd. for $C_{15}H_{31}NaNO_6$ $[M+Na]^+$ 322.223; found 322.260.

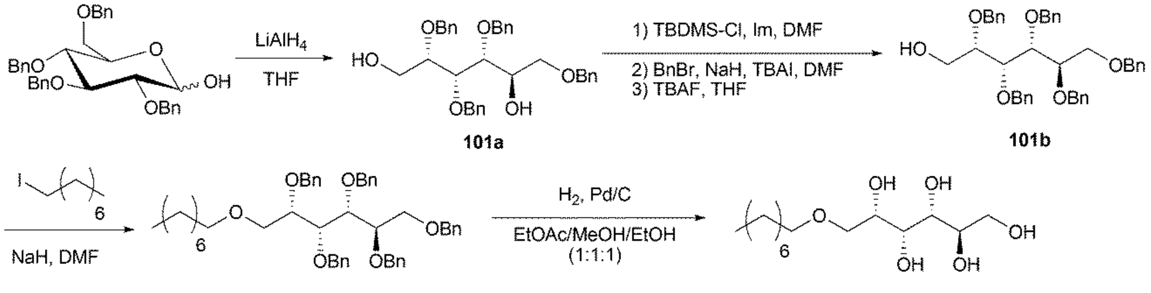

2,3,4,6-Tetra-O-benzyl-D-glucitol (101a)
To a stirred solution of 2,3,4,6-tetra-*O*-benzyl-D-glucopyranoside[35] (200 mg, 0.37 mmol) in THF was added $LiAlH_4$ (56 mg, 1.5 mmol). After stirring overnight at room temperature, the mixture was cooled to 0 °C and the excess of $LiAlH_4$ was carefully quenched with a few drops of water. The mixture was diluted with Et_2O and the organic phase was washed successively with 1% HCl, water and brine. The organic layer was dried over $MgSO_4$ and evaporated. Column chromatography (7:3 Pet. Ether/EtOAc) afforded **101a** (200 mg, quant.) as a viscous oil. ^{1}H NMR (400 MHz, $CDCl_3$): δ 7.39-7.18 (m, 20H), 4.70 (d, *J* = 11.7 Hz, 1H), 4.66 (d, *J* = 11.7 Hz, 1H), 4.65 (d, *J* = 11.7 Hz, 1H), 4.62 (d, *J* = 11.7 Hz, 1H), 4.58 (d, *J* = 11.2 Hz, 1H), 4.54 (d, *J* = 11.7 Hz, 1H), 4.53 (d, *J* = 11.2 Hz, 1H), 4.50 (d, *J* = 11.7 Hz, 1H), 4.02 (m, 1H), 3.88 (m, 1H), 3.77 (m, 2H), 3.72 (dd, J = 11.8, 4.5 Hz, 1H), 3.63 (m, 2H), 3.55 (dd, *J* = 11.7, 4.5 Hz, 1H), 2.27 (br, 2H). ^{13}C NMR (100 MHz, $CDCl_3$): δ 138.1, 137.9, 137.8, 137.8, 128.5, 128.4, 128.4, 128.4, 128.1, 128.0, 127.9, 127.9, 127.8, 127.8, 79.4, 79.1, 77.3, 74.5, 73.4, 73.2, 73.1, 71.1, 70.7, 61.8. LRMS (ESI): *m/z* calcd. for $C_{34}H_{42}NO_6$ $[M+NH_4]^+$ 560.3; found 560.3.

2,3,4,5,6-Penta-O-benzyl-D-glucitol (101b)
To a solution of **101a** (200 mg, 0.37 mmol) in DMF (2 mL) was added imidazole (60 mg, 0.89 mmol) and TBDMS-Cl (66 mg, 0.44 mmol), successively. After stirring overnight, the reaction mixture was diluted with Et_2O and washed with 1M $KHSO_4$, water and brine. The solvent was evaporated and crude material dried under high vacuum for several hours. The resulting alcohol was re-dissolved in DMF (5 mL) and NaH (18 mg, 0.44 mmol) was added. The mixture was stirred for 30 min and then benzyl bromide (66 µL, 0.55 mmol) and TBAI (14 mg, 0.04 mmol) were added. After stirring overnight, the mixture was diluted with water and organic phase was extracted with Et_2O. The organic layer was washed with water and brine, dried over $MgSO_4$ and the solvent evaporated. This crude material was re-dissolved in THF (2 mL) and TBAF (400 µL, 0.4 mmol) were added and the reaction stirred for 2 hours at room temperature. The mixture was diluted with Et_2O and the organic layerwas washed with water and brine, and the solvent was evaporated. Column chromatography (4:1 Pet. Ether/EtOAc) afforded **101b** (120 mg, 51%) as a colorless oil. ^{1}H NMR (500 MHz, $CDCl_3$): δ 7.33-7.19 (m, 25H), 4.74 (d, *J* = 11.4 Hz, 1H), 4.66 (d, *J* = 11.6 Hz,

1H), 4.63 (d, *J* = 12.0 Hz, 2H), 4.61 (d, *J* = 11.4 Hz, 1H), 4.57 (s, 2H), 4.50 (d, *J* = 11.9 Hz, 1H), 4.47 (d, *J* = 11.9 Hz, 1H), 4.43 (d, *J* = 11.6 Hz, 1H), 3.95 (t, *J* = 4.7 Hz, 1H), 3.86 (ddd, *J* = 8.3, 4.8, 3.5 Hz, 2H), 3.83 (dd, *J* = 5.4, 4.8 Hz, 1H), 3.70 (dd, *J* = 11.3, 6.0 Hz, 1H), 3.67 (m, 2H), 3.49 (q, *J* = 6.9 Hz, 1H). ^{13}C NMR ($CDCl_3$, 125 MHz): δ 138.6, 138.4, 138.3, 138.2, 138.2, 128.4, 128.3, 128.3, 128.3, 128.2, 128.1, 127.9, 127.7, 127.7, 127.6, 127.5, 127.4, 79.3, 79.3, 78.9, 78.6, 74.7, 73.8, 73.3, 72.7, 71.9, 69.6, 61.8. LRMS (ESI): *m/z* calcd. for $C_{41}H_{48}NO_6$ $[M+NH_4]^+$ 650.4; found 650.5.

2,3,4,5,6-Penta-O-benzyl-1-O-octyl-D-glucitol (101c)

To a solution of **101b** (95 mg, 0.15 mmol) in DMF (2 mL) was added NaH (12 mg, 0.30 mmol). The solution was stirred for 30 min then 1-iodooctane (54 µL, 0.30 mmol) was added and the mixture was stirred overnight. The following day, NaH (6 mg, 0.15 mmol) and 1-iodooctane (27 µL, 0.15 mmol) were added and the mixture was stirred for an additional 24 hours. The reaction mixture was then carefully quenched with water and extracted with Et_2O. The organic layer was washed with water and brine, dried over $MgSO_4$ and the solvent evaporated. Column chromatography (20:1 Pet. Ether/EtOAc) afforded **101c** (64 mg, 57%) as a viscous oil. ^{1}H NMR (400 MHz, $CDCl_3$): δ 7.32-7-20 (m, 25H), 4.72 (d, *J* = 11.9 Hz, 1H), 4.70 (d, *J* = 11.9 Hz, 1H), 4.62 (d, *J* = 11.9 Hz, 1H), 4.60 (s, 2H), 4.59 (d, *J* = 11.9 Hz, 1H), 4.59 (d, *J* = 11.9 Hz, 1H), 4.57 (d, *J* = 11.9 Hz, 1H), 4.42 (s, 2H), 3.96 (m, 3H), 3.89 (dd, *J* = 9.3, 4.7 Hz, 1H), 3.67 (dd, *J* = 10.0, 5.5 Hz, 1H) 3.64 (dd, *J* = 10.0, 4.7 Hz, 1H) 3.61 (dd, *J* = 10.1, 5.6 Hz, 1H) 3.57 (dd, *J* = 10.1, 4.7 Hz, 1H), 3.30 (t, *J* = 6.7 Hz, 2H), 1.50 (quint, *J* = 6.7 Hz, 2H), 1.32-1.20 (m, 10H), 0.87 (t, *J* = 7.0 Hz, 3H). ^{13}C NMR (100 MHz, $CDCl_3$): δ 138.9, 138.9, 138.8, 138.7, 138.2, 128.3, 128.2, 128.2, 127.8, 127.7, 127.7, 127.6, 127.5, 127.4, 127.3, 78.9, 78. 9, 78.4, 78.3, 73.7, 73.7, 73.2, 72.8, 72.6, 71.5, 71.0, 70.5, 31.8, 29.7, 29.5, 29.3, 26.2, 22.6, 14.1. LRMS (ESI): *m/z* calcd. for $C_{49}H_{64}NO_6$ $[M+NH_4]^+$ 762.5; found 762.6.

1-O-Octyl-D-glucitol (101)

A solution **101c** (60 mg, 0.13 mmol) in 3 mL of 1:1:1 mixture of EtOAc/EtOH/MeOH and 5% Pd/C (33 mg, 0.013 mmol) was stirred for 6 hours under an atmosphere of H_2. The flask was purged with N_2 and the catalyst removed by filtration through Celite®. The solvents were removed *in vacuo* and the solid was carefully washed with EtOAc to afford **101** (17 mg, 75%) as a white solid. ^{1}H NMR (500 MHz, D_2O): δ 3.90 (dt, *J* = 10.2, 6.1 Hz, 1H), 3.80-3.77 (m, 2H), 3.74-3.71 (m, 1H), 3.62-3.58 (m, 3H), 3.55-3.47 (m, 3H), 1.54 (quint, *J* = 6.7 Hz, 2H), 1.30-1.23 (m, 10H), 0.82 (t, *J* = 6.7 Hz, 3H). ^{13}C NMR (125 MHz, D_2O): δ 72.0, 71.8, 71.7, 71.6, 71.4, 70.0, 63.2, 31.7, 29.3, 29.2, 29.1, 25.9, 22.5, 13.8. LRMS (ESI): *m/z* calcd. for $C_{14}H_{30}NaO_6$ $[M+Na]^+$ 317.2; found 317.2.

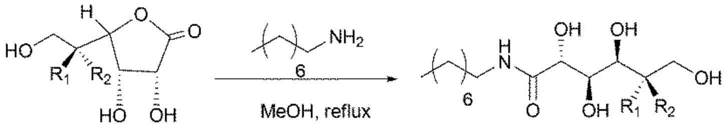

102: R_1 = OH, R_2 = H
103: R_1 = H, R_2 = OH

N-Octyl-L-Mannonamide (102)

To a solution of L-mannonic acid-γ-lactone (1.4 g, 7.86 mmol) in MeOH (30 mL) was added *n*-octylamine (1.3 mL, 7.86 mmol). The mixture was refluxed for 1 hour then cooled in an ice bath. The precipitate was filtered off and washed with cold MeOH to afford **102** as a white powder (1.25 g, 52%). Characterization data is consistent with that previously reported in the literature.[40, 41] ^{1}H NMR (300 MHz, DMSO-d_6): δ 7.88 (t, *J* = 5.8 Hz, 1H), 5.47 (d, *J* = 6.3 Hz, 1H), 4.47 (dt, *J* = 9.2, 4.9 Hz, 3H), 4.29 (t, *J* = 5.7 Hz, 1H), 3.87 (t, *J* = 6.7 Hz, 1H), 3.77 (t, *J* = 6.4 Hz, 1H), 3.62-3.57 (m, 1H), 3.49-3.45 (m, 2H), 3.39 (dd, *J* = 7.9, 4.4 Hz, 1H), 3.10-3.04 (m, 2H), 1.43-1.38 (m, 2H), 1.30-1.24 (m, 11H), 0.86 (t, *J* = 6.7 Hz, 3H). ^{13}C NMR (76 MHz, DMSO-d_6): δ 173.6, 71.9, 70.9, 70.5, 70.3, 63.8, 38.3, 31.3, 29.1, 28.78, 28.71, 26.4, 22.1, 14.0. LRMS (ESI): *m/z* calcd. for $C_{14}H_{30}NO_6$ $[M+H]^+$ 308.2; found 308.4.

***N*-Octyl-D-Gulonamide (103)**

To a solution of D-gulonic acid-*γ*-lactone (736 mg, 4.13 mmol) in MeOH (20 mL) was added *n*-octylamine (683 *µ*L, 4.13 mmol). The mixture was refluxed for 1 hour then cooled in an ice bath. The precipitate was filtered off and washed with cold MeOH to afford **103** as a white powder (697 mg, 55%). ^{1}H NMR (300 MHz, DMSO-d_6): δ 7.82 (t, *J* = 5.9 Hz, 1H), 5.44 (d, *J* = 6.5 Hz, 1H), 4.66 (d, *J* = 4.7 Hz, 1H), 4.48 (d, *J* = 4.6 Hz, 1H), 4.43 (t, *J* = 5.6 Hz, 1H), 4.36 (d, *J* = 5.9 Hz, 1H), 3.90 (t, *J* = 6.4 Hz, 1H), 3.62 (t, *J* = 5.0 Hz, 2H), 3.54 (t, *J* = 5.1 Hz, 1H), 3.43 (q, *J* = 5.4 Hz, 1H), 3.35 (t, *J* = 8.2 Hz, 1H), 3.09-3.03 (m, 2H), 1.42-1.38 (m, 2H), 1.28-1.20 (m, 10H), 0.86 (t, *J* = 6.7 Hz, 3H). ^{13}C NMR (76 MHz, DMSO-d_6): δ 173.6, 73.8, 73.3, 72.4, 70.0, 63.0, 38.8, 31.7, 29.5, 29.2, 29.2, 26.8, 22.6, 14.5. LRMS (ESI): *m/z* calcd. for $C_{14}H_{30}NO_6$ $[M+H]^+$ 308.2; found 308.3.

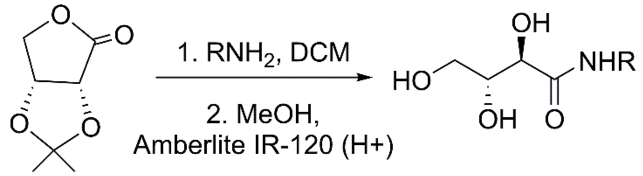

104: R = *n*-butyl
105: R = *n*-pentyl
106: R = *n*-hexyl
107: R = *n*-octyl

***N*-Butyl-erythronamide (104)**

To D-erythronolactone (0.2 g, 1.27 mmol) suspended in DCM (4 mL) was added butylamine (0.125 mL, 1.27 mmol). The reaction mixture was stirred at ambient temperature for 48 hours. Sodium bicarbonate was added to the crude mixture until the solid reside was dissolved. The aqueous layer was extracted three times with DCM, organics combined and washed once with brine, dried over $MgSO_4$ and solvent removed under reduced pressure to yield 99 mg of crude product. The crude product was dissolved in MeOH and Amberlite® IR-120 (H^+) ion-exchange resin was added and stirred for 24 hours. The mixture was filtered through celite, solvent removed under reduced pressure and product purified by flash chromatography (5% MeOH in DCM) to yield **104** as a white crystalline solid (41.6 mg, 42%). ^{1}H NMR (400 MHz, D_2O): δ 4.02 (d, *J* = 4.4 Hz, 1H), 3.77 (dt, *J* = 6.8, 4.2 Hz, 1H), 3.45 (m, 2H), 3.05 (t, *J* = 7.0 Hz, 2H), 1.32 (Quint, *J* = 7.5 Hz, 2H), 1.14 (Sext, *J* = 7.5Hz, 2H), 0.72 (t, *J* = 7.4Hz, 3H). ^{13}C NMR (100 MHz, D_2O): δ 173.5, 72.7, 72.6, 61.5, 38.7, 30.4, 19.3, 12.9. LRMS (ESI): *m/z* calcd. for $C_8H_{18}NO_4$ $[M+H]^+$ 192.1, found 192.1.

***N*-Pentyl-erythronamide (105)**

Compound **105** was made in a similar manner as **104** from D-erythronolactone (0.2 g, 1.27 mmol) and pentylamine (0.167 mL, 1.27 mmol). The product was purified by flash chromatography (6% MeOH in DCM) to yield **105** as a white crystalline solid (158 mg, 41%). ^{1}H NMR (400 MHz, D_2O): δ 4.01 (d, *J* = 4.5 Hz, 1H), 3.77 (dt, *J* = 6.8, 4.2 Hz, 1H), 3.46 (m, 2H), 3.06 (dt, *J* = 6.9, 1.5 Hz, 2H), 1.34 (Quint, *J* = 7.2 Hz, 2H), 1.11 (m, 4H), 0.69 (t, *J*= 7.1Hz, 3H). ^{13}C NMR (100 MHz, D_2O): δ 173.5, 72.7, 72.6, 61.4, 39.0, 28.2, 28.0, 21.6, 13.2. LRMS (ESI): *m/z* calcd. for $C_9H_{19}NNaO_4$ $[M+Na]^+$ 228.1, found 228.2.

***N*-Hexyl-erythronamide (106)**

Compound **106** was made in a similar manner as **104** from D-erythronolactone (1g, 8.48 mmol) and hexylamine (1.12 mL, 8.48 mmol). The product was purified by recrystallization in ethyl acetate to yield **106** as a white crystalline solid (0.78 g, 42%). ^{1}H NMR (400 MHz, D_2O): δ 4.01 (d, *J* = 4.5 Hz, 1H), 3.77 (dt, *J* = 6.8, 4.2 Hz, 1H), 3.46 (m, 2H), 3.04 (dt, *J* = 6.8, 1.5 Hz, 2H), 1.33 (quint, *J* = 7.2 Hz, 2H), 1.11 (m, 6H), 0.68 (t, *J* = 7.1 Hz, 3H). ^{13}C NMR (100 MHz, D_2O): δ 173.5, 72.7, 72.6, 61.5, 39.0, 30.6, 28.2, 25.6, 21.8, 13.2. LRMS (ESI): *m/z* calcd. for $C_{10}H_{21}NNaO_4$ $[M+Na]^+$ 242.1, found 242.2.

***N*-Octyl-erythronamide (107)**

Compound **107** was made in a similar manner as **104** from D-erythronolactone (1g, 8.48 mmol) and octylamine (1.40 mL, 8.48 mmol). The product was purified by recrystallization in ethyl acetate to yield **107** as a white crystalline solid (0.85 g, 43%). ^{1}H NMR (400 MHz, D_2O): δ 4.02 (d, *J* = 4.5 Hz, 1H), 3.77

(dt, J = 6.7, 4.3 Hz, 1H), 3.46 (m, 2H), 3.06 (dt, J = 6.8, 1.5 Hz, 2H), 1.33 (quint, J = 7.0 Hz, 2H), 1.11 (m, 10H), 0.68 (t, J= 7.1Hz, 3H). ^{13}C NMR (100 MHz, DMSO-d_6): d 172.6, 73.9, 73.3, 62.5, 38.6, 31.7, 29.6, 29.2, 29.1, 26.8, 22.6, 14.4. LRMS (ESI): *m/z* calcd. for $C_{12}H_{26}NO_4$ $[M+H]^+$ 248.2, found 248.3.

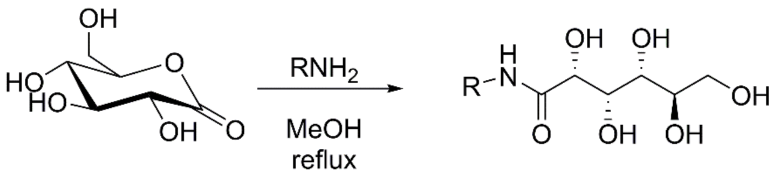

108: R = *n*-octyl
109: R = H
110: R = CH_3
111: R = CH_2CH_3
112: R = *n*-propyl
113: R = *n*-butyl
114: R = *n*-pentyl
115: R = *n*-hexyl
116: R = *n*-heptyl
117: R = *n*-nonyl

N-Octyl-D-gluconamide (108)
To a solution of D-gluconic acid-*d*-lactone (1.4 g, 7.86 mmol) in MeOH (30 mL), *n*-octylamine (1.3 mL, 7.86 mmol) was added. The mixture was heated to reflux for 1 hour then cooled in an ice bath. The precipitate was filtered off and washed with cold MeOH to afford **108** as a white powder (1.45 g, 60%). Characterization data is consistent with that previously reported in the literature.[40, 41] ^{1}H NMR (500 MHz, DMSO-d_6): δ 7.59, (t, J = 6.0 Hz, 1H), 5.34 (d, J = 5.1 Hz, 1H), 4.53 (t, J = 4.8 Hz, 1H), 4.47 (d, J = 5.1 Hz, 1H), 4.39 (d, J = 7.2 Hz, 1H), 4.33 (d, J = 5.8 Hz, 1H), 3.97 (dd, J = 4.9, 3.8 Hz, 1H), 3.89 (ddd, J = 7.2, 3.7, 2.2 Hz, 1H), 3.57 (m, 1H), 3.46 (m, 2H), 3.37 (m, 1H), 3.06 (m, 2H), 1.40 (quint, J = 6.6 Hz, 2H), 1.31-1.18 (m, 10H), 0.86 (t, J = 6.7 Hz, 3H). ^{13}C NMR (125 MHz, DMSO-d_6): δ 172.2, 73.6, 72.4, 71.5, 70.1, 63.4, 38.3, 31.3, 29.2, 28.8, 28.7, 26.4, 22.1, 14.0. LRMS (ESI): *m/z* calcd. for $C_{14}H_{30}NO_6$ $[M+H]^+$ 308.2; found 308.3.

Gluconamine (109)
To a solution of D-gluconic acid-*d*-lactone (0.5 g, 2.81 mmol) in MeOH (5 mL), ammonia (0.45 mL, 2.82 mmol) was added. The mixture was heated to reflux for 1 hour then cooled in an ice bath. The precipitate was filtered off and washed with cold MeOH to afford **109** as a white powder (0.40 g, 73%). ^{1}H NMR (400 MHz, D_2O): δ 4.19 (d, J = 3.6 Hz, 1H), 3.95 (t, J = 3.3 Hz, 1H), 3.70-3.50 (m, 4H). ^{13}C NMR (101 MHZ, D_2O): δ 177.6, 73.2, 72.1, 71.0, 70.2, 62.6. LRMS (ESI): *m/z c*alcd. for $C_6H_{14}NO_6$ $[M+H]^+$ 196.2, found 196.1.

N-Methyl-gluconamide (110)
To a solution of D-gluconic acid-*d*-lactone (0.5 g, 2.81 mmol) in MeOH (5 mL), methylamine hydrochloride (0.19 g, 2.81 mmol) was added, followed by 0.5 mL of DIPEA. The mixture was heated to reflux for 1 hour then cooled in an ice bath. The precipitate was filtered off and washed with cold MeOH to afford **110** as a white powder (0.36 g, 62%). ^{1}H NMR (400 MHz, D_2O): δ 4.17 (d, J = 3.6 Hz, 1H), 3.94 (t, J = 3.2 Hz, 1H), 3.70-3.50 (m, 4H), 2.65 (s, 3H). ^{13}C NMR (101 MHz, D_2O): δ 174.8, 73.3, 72.1, 71.1, 70.3, 62.6, 25.5. LRMS (ESI): *m/z* calcd. for $C_7H_{16}NO_6$ $[M+H]^+$ 210.2, found 210.1.

N-Ethyl-gluconamide (111)
To a solution of D-gluconic acid-*d*-lactone (0.5 g, 2.81 mmol) in MeOH (5 mL), ethylamine hydrochloride (0.23 g, 2.81 mmol) was added, followed by 0.5 mL of DIPEA. The mixture was heated to reflux for 1 hour then cooled in an ice bath. The precipitate was filtered off and washed with cold MeOH to afford **111** as a white powder (0.39 g, 63%). ^{1}H NMR (400 MHz, D_2O): δ 3.12 (q, J = 7.30 Hz, 1H), 3.73-3.45 (m, 1H), 4.00-3.87 (m, 1H), 4.14 (d, J = 3.73 Hz, 1H), 0.99 (t, J = 7.30 Hz, 1H). ^{13}C NMR (101 MHz, D_2O): δ 73.31, 72.10, 71.03, 70.29, 62.58, 13.57. LRMS (ESI): *m/z* calcd. for $C_8H_{18}NO_6$ $[M+H]^+$ 224.2, found 224.1.

N-Propyl-gluconamide (112)
To a solution of D-gluconic acid-*d*-lactone (0.5 g, 2.81 mmol) in MeOH (5 mL), propylamine (0.23 mL, 2.81 mmol) was added. The mixture was heated to reflux for 1 hour then cooled in an ice bath. The precipitate was filtered off and washed with cold MeOH to afford **112** as a white powder (0.42 g, 63%). ^{1}H NMR (300 MHz, D_2O): δ 0.77 (t, *J* = 7.5 Hz, 1H), 1.40 (td, *J* = 7.6, 3.9, 3.9 Hz, 1H), 3.75-3.45 (m, 1H), 4.02-3.88 (m, 1H), 3.09 (dd, *J* = 10.5, 3.6 Hz, 1H), 4.17 (dd, *J* = 2.2, 1.6 Hz, 1H). ^{13}C NMR (100 MHz, D_2O): δ 10.5, 21.9, 40.9, 62.6, 70.3, 71.1, 72.2, 73.4, 174.1. LRMS (ESI): *m/z* calcd. for $C_9H_{20}NO_6$ $[M+H]^+$ 238.3, found 238.2.

N-Butyl-gluconamide (113)
To a solution of D-gluconic acid-*d*-lactone (0.5 g, 2.81 mmol) in MeOH (5 mL), butylamine (0.28 mL, 2.81 mmol) was added. The mixture was heated to reflux for 1 hour then cooled in an ice bath. The precipitate was filtered off and washed with cold MeOH to afford **113** as a white powder (0.48 g, 68%). ^{1}H NMR (400 MHz, D_2O): δ 4.15 (d, *J* = 3.7 Hz, 1H), 0.77 (t, *J* = 11.8, 7.4 Hz, 1H), 1.18 (qd, *J* = 14.4, 7.3, 7.3, 7.2 Hz, 1H), 1.45-1.28 (m, 1H), 3.10 (t, *J* = 7.0 Hz, 1H), 3.97-3.88 (m, 1H), 3.72-3.45 (m, 1H). ^{13}C NMR (101 MHz, D_2O): δ 12.9, 19.3, 30.5, 38.8, 62.6, 70.3, 71.0, 72.2, 73.4, 174.0. LRMS (ESI): *m/z* *c*alcd. for $C_{10}H_{22}NO_6$ $[M+H]^+$ 252.3, found 252.3.

N-Pentyl-gluconamide (114)
To a solution of D-gluconic acid-*d*-lactone (0.5 g, 2.81 mmol) in MeOH (5 mL), pentylamine (0.33 mL, 2.81 mmol) was added. The mixture was heated to reflux for 1 hour then cooled in an ice bath. The precipitate was filtered off and washed with cold MeOH to afford **114** as a white powder (0.45 g, 61%). ^{1}H NMR (400 MHz, D_2O): δ 0.73 (t, *J* = 7.0 Hz, 1H), 1.22-1.10 (m, 1H), 1.44-1.33 (m,1H), 3.10 (t, *J* = 7.0 Hz, 1H), 3.70-3.55 (m, 1H), 3.50 (dd, *J* = 11.5, 5.9 Hz,1H), 3.95-3.91 (m, 1H), 4.14 (d, *J* = 3.8 Hz, 1H). ^{13}C NMR (101 MHz, D_2O): δ 13.2, 21.6, 28.0, 28.2, 39.1, 62.6, 70.3, 71.0, 72.2, 73.4, 174.0. LRMS (ESI): *m/z* calcd. for $C_{11}H_{24}NO_6$ $[M+H]^+$ 266.3, found 266.2.

N-Hexyl-gluconamide (115)
To a solution of D-gluconic acid-*d*-lactone (0.5 g, 2.81 mmol) in MeOH (5 mL), hexylamine (0.37 mL, 2.81 mmol) was added. The mixture was heated to reflux for 1 hour then cooled in an ice bath. The precipitate was filtered off and washed with cold MeOH to afford **115** as a white powder (0.47 g, 61%). ^{1}H NMR (400 MHz, DMSO-d_6): δ 0.82 (t, *J* = 6.8 Hz, 1H), 1.43-1.07 (m, 1H), 5.31 (d, *J* = 4.9 Hz, 1H), 4.58-4.24 (m, 1H), 4.01-3.76 (m, 1H), 3.63-3.37 (m, 1H), 3.02 (dd, *J* = 13.5, 6.8 Hz, 1H), 7.56 (t, *J* = 5.9 Hz, 1H). ^{13}C NMR (101 MHz, DMSO-d_6): δ 14.4, 22.5, 26.5, 29.6, 31.5, 38.7, 63.8, 70.6, 72.0, 72.9, 74.1, 172.7. LRMS (ESI): *m/z c*alcd. for $C_{12}H_{26}NO_6$ $[M+H]^+$ 280.3, found 280.2.

N-Heptyl-gluconamide (116)
To a solution of D-gluconic acid-*d*-lactone (0.5 g, 2.81 mmol) in MeOH (5 mL), heptylamine (0.42 mL, 2.81 mmol) was added. The mixture was heated to reflux for 1 hour then cooled in an ice bath. The precipitate was filtered off and washed with cold MeOH to afford **135** as a white powder (0.56 g, 68%). ^{1}H NMR (400 MHz, DMSO-d_6): δ 0.82 (t, *J* = 6.9 Hz, 1H), 1.42-1.13 (m, 1H), 2.46 (td, *J* = 3.6, 1.8 Hz, 1H), 3.15-2.94 (m, 1H), 3.42 (dd, *J* = 4.8, 2.3 Hz, 1H), 3.97-3.81 (m, 1H), 4.50 (d, *J* = 4.9 Hz, 1H), 4.43 (d, *J* = 5.3 Hz, 1H), 4.38-4.25 (m, 1H), 5.31 (d, *J* = 5.1 Hz, 1H), 7.56 (s, 1H). ^{13}C NMR (101 MHz, DMSO-d_6): δ 14.4, 26.8, 28.9, 29.6, 31.7, 38.7, 63.8, 70.6, 72.0, 72.9, 74.1, 172.7. LRMS (ESI): *m/z* calcd. for $C_{13}H_{28}NO_6$ $[M+H]^+$ 294.4, found 294.1.

N-Nonyl-gluconamide (117)
To a solution of D-gluconic acid-*d*-lactone (0.5 g, 2.81 mmol) in MeOH (5 mL), nonylamine (0.52 mL, 2.81 mmol) was added. The mixture was heated to reflux for 1 hour then cooled in an ice bath. The

precipitate was filtered off and washed with cold MeOH to afford **136** as a white powder (0.62 g, 69%). ^{1}H NMR (400 MHz, DMSO-d_6): δ 0.82 (t), 1.43-1.12 (m), 3.14-2.94 (m), 3.47-3.40 (m), 3.95-3.81 (m), 7.56 (t), 5.31 (d), 4.50 (d), 4.43 (d), 4.38-4.27 (m), 2.46 (td). ^{13}C NMR (101 MHz, DMSO-d_6): δ 14.4, 22.6, 26.8, 29.2, 29.3, 29.5, 29.6, 31.8, 38.7, 63.8, 70.6, 72.0, 72.9, 74.1, 172.7. LRMS (ESI): *m/z* calcd. for $C_{15}H_{32}NO_6$ $[M+H]^+$ 322.4, found 322.2.

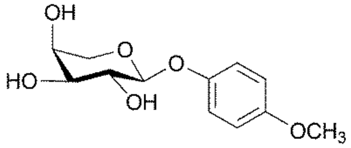
118

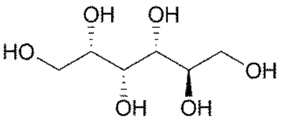
119

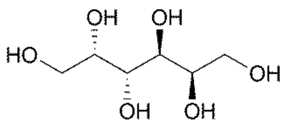
120

Compounds **118-120** are commercially available.

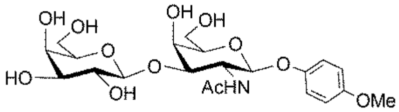
121

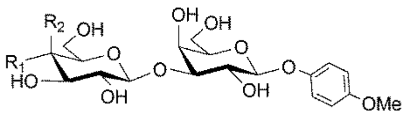
122: R_1 = OH, R_2 = H
123: R_1 = H, R_2 = OH

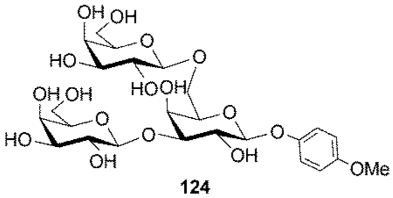
124

Compounds **121-124** were graciously provided by Dr. Mark S. Taylor's laboratory from the Department of Chemistry at the University of Toronto.

Synthesis of Compounds used for the Prediction Set:

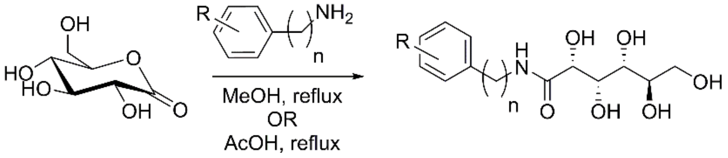

125: n = 0, R = 4-CF_3
126: n = 0, R = 4-CH_3
127: n = 1, R = 3-CF_3
128: n = 1, R = 4-CF_3
129: n = 0, R = 2-OH
130: n = 0, R = 3-CF_3
131: n = 0, R = 4-OCF_3
132: n = 0, R = 2,5-dichloro
133: n = 1, R = 2-CH_3
134: n = 1, R = 4-OCF_3
135: n = 1, R = 2-OCF_3
136: n = 0, R = 2-Cl
137: n = 0, R = 4-Cl
138: n = 1, R = 4-Cl
139: n = 0, R = 2-CH_3
140: n = 0, R = 2,6-dimethyl
141: n = 0, R = 2,5-dimethyl

N-4-(Trifluoromethyl)phenyl-D-gluconamide (125)

To a solution of D-gluconic acid-*d*-lactone (0.10 g, 0.56 mmol) in acetic acid (5 mL) was added 4-(trifluoromethyl)aniline (0.10 mL, 0.56 mmol). The mixture was stirred under reflux for 2 hours. The crude product was precipitated with hexanes, filtered and crude solid was recrystallized in EtOH to afford **125** as white crystals (89 mg, 47%). ^{1}H NMR (500 MHz, DMSO-d_6): δ 9.94 (s, 1H), 7.96 (d, *J* = 8.9 Hz, 2H), 7.67 (d, *J* = 8.9 Hz, 2H), 5.78 (d, *J* = 5.2 Hz, 1H), 4.57-4.62 (m, 3H), 4.38 (t, *J* = 5.7 Hz, 1H), 4.21 (dd, *J* = 3.9, 5.3 Hz, 1H), 4.01-4.03 (m, 1H), 3.57-3.61 (m, 1H), 3.50-3.52 (m, 2H), 3.37-3.41 (m, 1H). ^{13}C NMR (100 MHz, DMSO-d_6): δ 172.3, 142.2, 125.8 (d, *J* = 3.8 Hz), 123.3 (d, *J* = 33.0 Hz), 119.5, 74.3, 72.1, 71.5, 70.4, 63.3. LRMS (ESI): *m/z* calcd. for $C_{13}H_{16}F_3NNaO_6$ $[M+Na]^+$ 362.1, found 362.0.

N-4-Methylphenyl-D-gluconamide (126)

To a solution of D-gluconic acid-*d*-lactone (0.20 g, 1.12 mmol) in methanol (10 mL) was added 4-methylaniline (0.12 g, 1.12 mmol). The mixture was stirred under reflux for 24 hours. The solvent was

evaporated and the crude product was recrystallized in EtOH to afford **126** as white crystals (12 mg, 5%). ^{1}H NMR (400 MHz, DMSO-*d₆*): δ 9.41 (s, 1H), 7.58 (d, *J* = 8.4 Hz, 2H), 7.10 (d, *J* = 8.6 Hz, 2H), 5.66 (d, *J* = 5.3 Hz, 1H), 4.58 (d, *J* = 5.1 Hz, 1H), 4.51-4.54 (m, 2H), 4.35 (t, *J* = 5.7 Hz, 1H), 4.15 (dd, *J* = 4.8, 3.8 Hz, 1H), 3.99-4.02 (m, 1H), 3.56-3.61 (m, 1H), 3.49-3.52 (m, 2H), 3.37-3.41 (m, 1H), ^{13}C NMR (100 MHz, DMSO-d_6): δ 172.2, 136.0, 132.2, 128.9. 119.5, 74.2, 72.3, 71.5, 70.3, 63.3, 20.5. LRMS (ESI): *m/z* calcd. for $C_{13}H_{19}NNaO_6$ [M+Na]$^+$ 308.1, found 308.1.

N-3-(Trifluoromethyl)benzyl-D-gluconamide (127)
To a solution of D-gluconic acid-*d*-lactone (0.20 g, 1.12 mmol) in methanol (10 mL) was added 3-(trifluoromethyl)benzylamine (0.32 mL, 2.24 mmol). The mixture was stirred under reflux for 24 hours. The solvent was evaporated and the crude product was recrystallized in EtOH to afford **127** as white crystals (245 mg, 62%). ^{1}H NMR (400 MHz, DMSO-d_6): δ 8.36 (t, *J* = 6.4 Hz, 1H), 7.51-7.65 (m, 4H), 5.5 (d, *J* = 5.1 Hz, 1H), 4.55 (dd, *J* = 17.8, 5.1 Hz, 2H), 4.34-4.45 (m, 4H), 4.09 (dd, *J* = 3.5, 5.1 Hz, 1H), 3.95-3.98 (m, 1H), 3.56-3.61 (m,1H), 3.49-3.51 (m, 2H), 3.35-3.40 (m, 1H). ^{13}C NMR (100 MHz, DMSO-d_6): δ 172.9, 141.2, 131.2, 129.1, 123.6, 123.2, 73.8, 72.5, 71.6, 70.2, 63.3, 41.4. LRMS (ESI): *m/z* calcd. for $C_{14}H_{18}F_3NNaO_6$ [M+Na]$^+$ 376.1, found 375.9.

N-4-(Trifluoromethyl)benzyl-D-gluconamide (128)
To a solution of D-gluconic acid-*d*-lactone (0.20 g, 1.12 mmol) in methanol (10 mL) was added 4-(trifluoromethyl)benzylamine (0.32 mL, 2.24 mmol). The mixture was stirred under reflux for 24 hours. The solvent was evaporated and the crude product was recrystallized in EtOH to afford **128** as white crystals (367 mg, 93%). ^{1}H NMR (400 MHz, DMSO-d_6): δ 8.34 (t, *J* = 6.4 Hz, 1H), 7.65 (d, *J* = 8.5 Hz, 2H), 7.5 (d, *J* = 8.2 Hz, 2H), 5.49 (d, *J* = 4.9 Hz, 1H), 4.55 (dd, *J* = 18.6, 4.1 Hz, 2H), 4.49 (d, *J* = 7.3 Hz, 1H), 4.34-4.45 (m, 3H), 4.09 (t, *J* = 3.7 Hz, 1H), 3.95-3.98 (m, 1H), 3.56-3.61 (m, 1H), 3.49-3.51 (m, 2H), 3.35-3.41 (m, 1H). ^{13}C NMR (100 MHz, DMSO-d_6): δ 172.9, 144.6, 127.7, 124.9, 73.9, 72.4, 71.5, 70.2, 63.3, 41.5. LRMS (ESI): *m/z* calcd. for $C_{14}H_{18}F_3NNaO_6$ [M+Na]$^+$ 376.1, found 375.9.

N-2-Hydroxyphenyl-D-gluconamide (129)
To a solution of D-gluconic acid-*d*-lactone (0.10 g, 0.56 mmol) in acetic acid (5 mL) was added 2-aminphenol (0.12 g, 1.12 mmol). The mixture was stirred under reflux for 2 hours. The crude product was precipitated with hexanes, filtered and crude solid was recrystallized in EtOH to afford **129** as white crystals (100 mg, 62%). ^{1}H NMR (500 MHz, DMSO-d_6): δ 11.05 (s, 1H), 10.23 (s, 1H), 9.21 (dd, *J* = 1.4, 7.7 Hz, 1H), 7.85-7.89 (m, 2H), 7.74-7.77 (m, 1H), 6.99 (d, *J* = 4.6 Hz, 1H), 5.59-5.63 (m, 3H), 5.38 (t, *J* = 5.7 Hz, 1H), 5.16 (dd, *J* = 3.1, 4.6 Hz, 1H), 5.00-5.03 (m, 1H), 4.53-4.59 (m, 2H), 4.47-4.51 (m, 1H), 4.36-4.40 (m, 1H). ^{13}C NMR (100 MHz, DMSO-d_6): δ 170.8, 145.9, 126.3, 123.4, 119.1, 118.8, 114.6, 74.1, 72.5, 71.6, 70.1, 63.3. LRMS (ESI): *m/z* calcd. for $C_{12}H_{17}NNaO_7$ [M+Na]$^+$ 310.1, found 310.1.

N-3-(Trifluoromethyl)phenyl-D-gluconamide (130)
To a solution of D-gluconic acid-*d*-lactone (0.60 g, 3.37 mmol) in acetic acid (10 mL) was added 3-(trifluoromethyl)aniline (0.84 mL, 6.74 mmol). The mixture was stirred under reflux for 1 hour. The crude product was precipitated with hexanes, filtered and crude solid was recrystallized in EtOH to afford **130** as white crystals (685 mg, 59%). ^{1}H NMR (400 MHz, DMSO-d_6): δ 9.93 (s, 1H), 8.26 (t, *J* = 2.0 Hz, 1H), 7.96 (dd, *J* = 2.3, 8.4 Hz, 1H), 7.54 (t, *J* = 8.1 Hz, 1H), 7.4 (dd, *J* = 1.8, 7.7 Hz, 1H), 5.74 (d, *J* = 5.2 Hz, 1H), 4.55-4.59 (m, 3H), 4.36 (t, *J* = 5.6 Hz, 1H), 4.21 (dd, *J* = 3.7, 5.2 Hz, 1H), 4.01-4.04 (m, 1H), 3.57-3.62 (m, 1H), 3.50-3.53 (m, 2H), 3.37-3.42 (m, 1H). ^{13}C NMR (100 MHz, DMSO-d_6): δ 172.3, 139.4, 129.7, 123.2, 119.6, 115.8, 74.2, 72.1, 71.5, 70.4, 63.3. LRMS (ESI): *m/z* calcd. for $C_{13}H_{16}F_3NNaO_6$ [M+Na]$^+$ 362.1, found 361.9.

N-4-(Trifluoromethoxy)phenyl-D-gluconamide (131)
To a solution of D-gluconic acid-*d*-lactone (0.50 g, 2.80 mmol) in acetic acid (10 mL) was added 4-(trifluoromethoxy)aniline (0.75 mL, 5.60 mmol). The mixture was stirred under reflux for 1 hour. The

crude product was precipitated with hexanes, filtered and crude solid was recrystallized in EtOH to afford **131** as white crystals (580 mg, 58%). ^{1}H NMR (400 MHz, DMSO-d_6): δ 9.77 (s, 1H), 7.84 (d, *J* = 9.1 Hz, 2H), 7.31 (d, *J* = 9.0 Hz, 2H), 5.72 (d, *J* = 5.2 Hz, 1H), 4.58 (d, *J* = 4.0 Hz, 1H), 4.53-4.55 (m, 2H), 4.35 (t, *J* = 5.7 Hz, 1H), 4.19 (dd, *J* = 3.9, 5.3 Hz, 1H), 3.99-4.03 (m, 1H), 3.57-3.60 (m,1H), 3.51-3.52 (m, 2H), 3.37-3.42 (m, 1H). ^{13}C NMR (100 MHz, DMSO-d_6): δ 171.8, 143.6, 137.8, 121.4, 121.0, 74.2, 72.2, 71.5, 70.3, 63.3. LRMS (ESI): *m/z* calcd. for $C_{13}H_{16}F_3NNaO_7$ [M+Na]$^+$ 378.1, found 377.9.

***N*-2,5-(Dichloro)phenyl-D-gluconamide (132)**

To a solution of D-gluconic acid-*d*-lactone (0.20 g, 1.12 mmol) in acetic acid (10 mL) was added 2,5-(dichloro)aniline (0.22 g, 1.34 mmol). The mixture was stirred under reflux for 3 hours. The crude product was precipitated with hexanes, filtered and crude solid was recrystallized in EtOH to afford **132** as white crystals (17 mg, 5%). ^{1}H NMR (400 MHz, DMSO-d_6): δ 9.43 (s, 1H), 8.39 (s, 1H), 7.53 (d, *J* = 8.7 Hz, 1H), 7.18 (d, *J* = 8.2 Hz, 1H), 6.16 (d, *J* = 4.3 Hz, 1H), 4.71 (d, *J* = 6.9 Hz, 1H), 4.61 (t, *J* = 6.1 Hz, 2H), 4.35 (t, *J* = 6.1 Hz, 1H), 4.24 (s, 1H), 4.00 (s, 1H), 3.54 (s, 2H), 3.47 (s, 1H), 3.36 (quint, *J* = 5.2, 5.6 Hz, 1H). ^{13}C NMR (100 MHz, DMSO-d_6): δ 172.5, 135.9, 132.6, 131.1, 124.8, 121.4, 120.5, 74.4, 72.5, 72.1, 70.8, 63.7. LRMS (ESI): *m/z* calcd. for $C_{12}H_{15}Cl_2NNaO_6$ [M+Na]$^+$ 363.1, found 363.9.

***N*-2-Methylbenzyl-D-gluconamide (133)**

To a solution of D-gluconic acid-*d*-lactone (0.10 g, 0.56 mmol) in methanol (10 mL) was added 2-methylbenzylamine (0.10 mL, 0.56 mmol). The mixture was stirred under reflux for 24 hours. The solvent was evaporated and the crude product was recrystallized in EtOH to afford **133** as white crystals (123 mg, 73%). ^{1}H NMR (400 MHz, DMSO-d_6): δ 7.97 (t, *J* = 6.1 Hz, 1H), 7.27-7.11 (m, 4H), 5.46 (d, *J* = 4.8 Hz, 1H), 4.58 (d, *J* = 3.9 Hz, 1H), 4.52 (d, *J* = 4.2 Hz, 1H), 4.46 (d, *J* = 6.3 Hz, 1H), 4.35 (t, *J* = 4.8 Hz, 1H), 4.28 (d, *J* = 5.9 Hz, 2H), 4.10 (t, *J* = 4.6 Hz, 1H), 3.98-3.95 (m, 1H), 3.60-3.58 (m, 1H), 3.50 (s, 2H), 3.39-3.37 (m, 1H), 2.26 (s, 3H). ^{13}C NMR (100 MHz, DMSO-d_6): δ 172.4, 137.0, 135.3, 129.7, 127.2, 126.6, 125.6, 73.9, 72.5, 71.5, 70.2, 63.4, 18.6. LRMS (ESI): *m/z* calcd. for $C_{14}H_{22}NO_6$ [M + H]$^+$ 300.1, found 300.1.

***N*-4-(Trifluoromethoxy)benzyl-D-gluconamide (134)**

To a solution of D-gluconic acid-*d*-lactone (0.10 g, 0.56 mmol) in methanol (10 mL) was added 4-(trifluoromethoxy)benzylamine (0.17 mL, 0.56 mmol). The mixture was stirred under reflux for 24 hours. The solvent was evaporated and the crude product was recrystallized in EtOH to afford **134** as white crystals (180 mg, 87%). ^{1}H NMR (500 MHz, DMSO-d_6): δ 8.28 (t, *J* = 6.4 Hz, 1H), 7.4 (d, *J* = 8.6 Hz, 2H), 7.28 (d, *J* = 8.6 Hz, 2H), 5.47 (d, *J* = 5.0 Hz, 1H), 4.55 (dd, *J* = 19.2, 5.1 Hz, 2H), 4.48 (d, *J* = 7.4 Hz, 1H), 4.27-4.36 (m, 3H), 4.07 (dd, *J* = 3.6, 5.0 Hz, 1H), 3.96-3.87 (m, 1H), 3.56-3.61 (m, 1H), 3.49-3.50 (m, 2H), 3.35-3.40 (m, 1H). ^{13}C NMR (100 MHz, DMSO-d_6): δ 172.7, 147.0, 139.2, 128.9, 120.7, 73.9, 72.5, 71.5, 70.1, 63.2, 41.1. LRMS (ESI): *m/z* calcd. for $C_{14}H_{18}F_3NNaO_7$ [M+Na]$^+$ 392.1, found 392.0.

***N*-2-(Trifluoromethoxy)benzyl-D-gluconamide (135)**

To a solution of D-gluconic acid-*d*-lactone (0.20 g, 1.12 mmol) in methanol (10 mL) was added 2-(trifluoromethoxy)benzylamine (0.43 g, 2.24 mmol). The mixture was stirred under reflux for 24 hours. The solvent was evaporated and the crude product was recrystallized in EtOH to afford **135** as white crystals (267 mg, 64%). ^{1}H NMR (500 MHz, DMSO-d_6): δ 8.23 (t, *J* = 6.2 Hz, 1H), 7.47 (dd, *J* = 2.3, 7.2 Hz, 1H), 7.31-7.38 (m, 3H), 5.52 (d, *J* = 5.0 Hz, 1H), 4.56 (dd, *J* = 18.7, 4.7 Hz, 2H), 4.51 (d, *J* = 7.0 Hz, 1H), 4.34-4.38 (m, 3H), 4.10 (dd, *J* = 3.6, 5.1 Hz, 1H), 3.96-3.98 (m, 1H), 3.57-3.61 (m, 1H), 3.49-3.52 (m, 2H), 3.36-3.41 (m, 1H). ^{13}C NMR (100 MHz, DMSO-d_6): δ 173.0, 145.9, 131.9, 128.9, 128.3, 127.3, 120.4, 73.9, 72.5, 71.6, 70.2, 63.3, 36.3. LRMS (ESI): *m/z* calcd. for $C_{14}H_{18}F_3NNaO_7$ [M+Na]$^+$ 392.1, found 392.0.

***N*-2-Chlorophenyl-D-gluconamide (136)**
To a solution of D-gluconic acid-*d*-lactone (0.20 g, 1.12 mmol) in acetic acid (5 mL) was added 2-chloroaniline (0.12 mL, 1.12 mmol). The mixture was stirred under reflux for 2 hours. The crude product was precipitated with hexanes, filtered and crude solid was recrystallized in EtOH to afford **136** as white crystals (10 mg, 3%). ^{1}H NMR (400 MHz, DMSO-d_6): δ 9.40 (s, 1H), 8.34 (dd, *J* = 1.6, 8.3 Hz, 1H), 7.52 (dd, *J* = 1.5, 8.0 Hz, 1H), 7.35 (dt, *J* = 1.6, 7.8 Hz, 1H), 7.13 (dt, *J* = 1.6, 7.7 Hz, 1H), 6.14 (d, *J* = 4.7 Hz, 1H), 4.7 (d, *J* = 7.4 Hz, 1H), 4.65 (dd, *J* = 2.8, 6.0 Hz, 2H), 4.39 (t, *J* = 5.7 Hz, 1H), 4.26 (dd, *J* = 3.1, 4.8 Hz, 1H), 4.05 (td, *J* = 3.0, 7.5 Hz, 1H), 3.48-3.61 (m, 3H), 3.37-3.43 (m, 1H). ^{13}C NMR (100 MHz, DMSO-d_6): δ 171.5, 134.4, 129.2, 127.8, 124.7, 122.3, 120.8, 74.0, 72.2, 71.6, 70.2, 63.2. LRMS (ESI): *m/z* calcd. for $C_{12}H_{16}ClNNaO_6$ [M+Na]$^+$ 328.1, found 328.0.

***N*-4-Chlorophenyl-D-gluconamide (137)**
To a solution of D-gluconic acid-*d*-lactone (0.20 g, 1.12 mmol) in acetic acid (5 mL) was added 4-chloroaniline (0.12 mL, 1.12 mmol). The mixture was stirred under reflux for 2 hours. The crude product was precipitated with hexanes, filtered and crude solid was recrystallized in EtOH to afford **137** as white crystals (180mg, 52%). ^{1}H NMR (400 MHz, DMSO-d_6): δ 9.70 (s, 1H), 7.80 (d, *J* = 9.1 Hz, 2H), 7.35 (d, *J* = 8.8 Hz, 2H), 5.71 (d, *J* = 5.3 Hz, 1H), 4.59 (d, *J* = 4.9 Hz, 1H), 4.55-4.53 (m, 2H), 4.36 (t, *J* = 5.7 Hz, 1H), 4.18 (dd, *J* = 5.1, 3.7 Hz, 1H), 4.02-3.99 (m, 1H), 3.61-3.57 (m, 1H), 3.52-3.51 (m, 2H), 3.42-3.36 (m, 1H). ^{13}C NMR (100 MHz, DMSO-d_6): δ 171.8, 137.6, 128.4, 126.9, 121.2, 74.2, 72.2, 71.5, 70.3, 63.3. LRMS (ESI): *m/z* calcd. for $C_{12}H_{16}ClNNaO_6$ [M+Na]$^+$ 328.1, found 328.0.

***N*-4-Chlorobenzyl-D-gluconamide (138)**
To a solution of D-gluconic acid-*d*-lactone (0.89 g, 5.02 mmol) in MeOH (30 mL) was added 4-chlorobenzylamine (0.8 mL, 5.02 mmol). The mixture was stirred under reflux for 48 hours. The solvent was evaporated and the residue was recrystallized in EtOH to afford **138** as white crystals (760 mg, 85%). Characterization data is consistent with that of previously reported data.[32] ^{1}H NMR (400 MHz, DMSO-d_6): δ 8.24 (t, *J* = 6.3 Hz, 1H), 7.32 (dd, *J* = 16.1, 8.8 Hz, 4H), 5.47 (d, *J* = 5.0 Hz, 1H), 4.58 (d, *J* = 4.7 Hz, 1H), 4.52 (d, *J* = 4.6 Hz, 1H), 4.47 (d, *J* = 7.2 Hz, 1H), 4.36 (t, *J* = 5.6 Hz, 1H), 4.26-4.33 (m, 2H), 4.07 (t, *J* = 4.0 Hz, 1H), 3.96-3.97 (m, 1H), 3.57-3.61 (m, 1H), 3.50 (m, 2H), 3.37-3.41 (m, 1H). ^{13}C NMR (100 MHz, DMSO-d_6): δ 172.7, 138.7, 131.1, 129.0, 128.0, 73.9, 72.5, 71.6, 70.2, 63.4, 41.2. LRMS (ESI): *m/z* calcd. for $C_{13}H_{18}ClNNaO_6$ [M+Na]$^+$ 342.1, found 342.2.

***N*-2-Methylphenyl-D-gluconamide (139)**
To a solution of D-gluconic acid-*d*-lactone (0.50 g, 2.81 mmol) in acetic acid (5 mL) was added 2-methylaniline (0.6 mL, 5.62 mmol). The mixture was stirred under reflux for two hours. The crude product was precipitated with hexanes, filtered and crude solid was recrystallized in EtOH to afford **139** as white crystals (705 mg, 88%). ^{1}H NMR (400 MHz, DMSO-d_6): δ 9.00 (s, 1H), 7.74 (d, *J* = 7.6 Hz, 1H), 7.15-7.22 (m, 2H), 7.03-7.06 (m, 1H), 5.83 (d, *J* = 4.8 Hz, 1H), 4.58-4.64 (m, 3H), 4.37 (t, *J* = 5.9 Hz, 1H), 4.20-4.22 (m, 1H), 4.01-4.05 (m, 1H), 3.51-3.62 (m, 3H), 3.37-3.43 (m, 1H), 2.22 (s, 3H). ^{13}C NMR (100 MHz, DMSO-d_6): δ 171.1, 136.0, 130.2, 129.4, 126.1, 124.4, 122.5, 74.1, 72.3, 71.6, 70.2, 63.3, 17.3. LRMS (ESI): *m/z* calcd. for $C_{13}H_{19}NaNO_6$ [M+Na]$^+$ 308.1, found 308.2.

***N*-2,6-Dimethylphenyl-D-gluconamide (140)**
To a solution of D-gluconic acid-*d*-lactone (0.50 g, 2.81 mmol) in acetic acid (5 mL) was added 2,6-dimethylaniline (0.7 mL, 5.62 mmol). The mixture was stirred under reflux for two hours. The crude product was precipitated with hexanes, filtered and crude solid was recrystallized in EtOH to afford **540** as white crystals (690 mg, 82%). ^{1}H NMR (400 MHz, DMSO-d_6): δ 9.02 (s, 1H), 7.05-7.07 (m, 3H), 5.62 (d, *J* = 5.0, 1H), 4.59 (d, *J* = 5.0 Hz, 1H), 4.55 (d, *J* = 5.4 Hz, 1H), 4.50 (d, *J* = 7.3 Hz, 1H), 4.37 (t, *J* = 5.5 Hz, 1H), 4.21 (t, *J* = 4.1 Hz, 1H), 4.02-4.05 (m, 1H), 3.52-3.63 (m, 3H), 3.39-3.43 (m, 1H), 2.15 (s,

6H). ^{13}C NMR (100 MHz, DMSO-d_6): δ 171.1, 135.4, 135.0, 127.5, 126.2, 74.2, 72.6, 71.6, 70.3, 63.4, 18.2. LRMS (ESI): *m/z* calcd. for $C_{14}H_{21}NNaO_6$ $[M+Na]^+$ 322.1, found 322.2.

***N*-2,5-Dimethylphenyl-D-gluconamide (141)**

To a solution of D-gluconic acid-*d*-lactone (0.50 g, 2.81 mmol) in acetic acid (5 mL) was added 2,5-dimethylaniline (0.7 mL, 5.62 mmol). The mixture was stirred under reflux for two hours. The crude product was precipitated with hexanes, filtered and crude solid was recrystallized in EtOH to afford **541** as white crystals (650 mg, 77%). ^{1}H NMR (400 MHz, DMSO-d_6): δ 8.94 (s, 1H), 7.59 (s, 1H), 7.08 (d, J = 7.7 Hz, 1H), 6.86 (d, J = 7.4 Hz, 1H), 5.82 (d, J = 4.4 Hz, 1H), 4.58 (m, 3H), 437 (t, J = 5.6 Hz, 1H), 4.19-4.21 (m, 1H), 4.01-4.04 (m, 1H), 3.51-3.63 (m, 3H), 3.38-3.49 (m, 1H), 2.25 (s, 3H), 2.16 (s, 3H). ^{13}C NMR (100 MHz, DMSO-d_6): δ 171.1, 135.8, 135.1, 130.0, 126.2, 125.0, 123.0, 74.1, 72.4, 71.6, 70.2, 63.3, 20.8, 16.9. LRMS (ESI): *m/z* calcd. for $C_{14}H_{21}NNaO_6$ $[M+Na]^+$ 322.1, found 322.2.

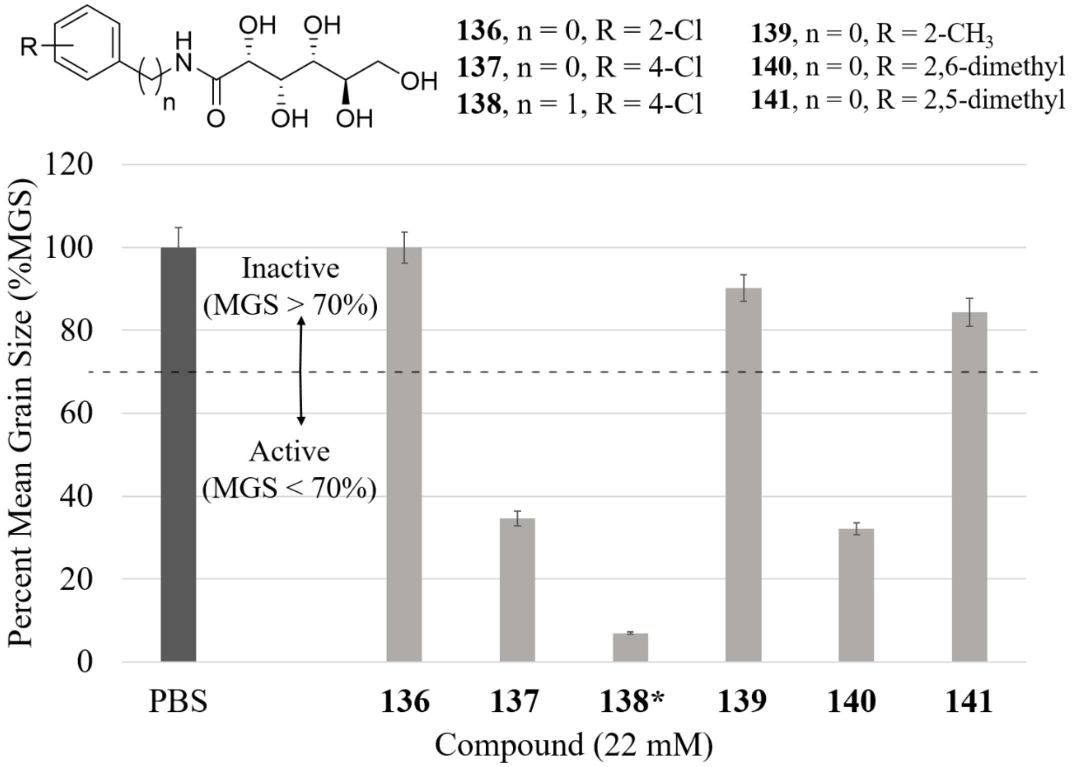

Figure S1. IRI activity of compounds predicted to be inactive (**136-141**) at 22 mM (except those marked with asterisks which were measured at 11 mM). The dark grey bar represents the PBS control. The dotted line represents the cutoff for activity where a MGS > 70% is considered inactive and a MGS < 70% is considered active.

Computational Details

Three-Dimensional Structure Generation

The initial set of 124 structures with experimental data was given in the format of 2D ChemDraw structures. Thus, 3D structures had to be generated and the optimal conformation for the QSAR model had to be determined. The models were manually generated using the Spartan program[42] and optimized using the Merck Molecular Force Field (MMFF)[43] before a conformational search was done. The conformational

search was done using a Monte-Carlo algorithm as implemented in Spartan.[42] The energy was evaluated at the PM6 level of theory.[44] This procedure was carried out until 100 conformers were generated. In order to maintain similarity within the structures the conformational search was done for a base molecule and then this structure was functionalized rather than a conformational search done on all functionalized molecules separately. Finally, single point DFT calculations at the B3LYP/6-311G(d,p) level of theory were performed using the Gaussian 09 software[45] to calculate the total electronic density distribution as well as the electrostatic potential.

Molecular Fingerprint Generation

The molecular surface was generated from the DFT-derived electronic density distribution. The molecular surface was defined by the total electron density isosurface value of 0.0135 electrons/Bohr^3, which was empirically shown to correspond to the vdW surface.[46, 47] The values were generated at nodes separated 0.176 Å apart on a rectangular grid. This was done for both the total electronic density where the values at the nodes are the curvature and for the electrostatic potential where the values of the nodes are the electrostatic potential at that node. This allows for the steric and electrostatic features to be represented on the molecular surface. The maximum product of the values at the surface nodes are now used as maximum auto- and cross correlation (MACC) scores to develop alignment-independent fingerprints. These maximum products are assigned to a distance bin according to the node separation whereby the number of bins was 200. Thus, at each specific distance bin is the maximum curvature-curvature, esp-esp, or curvature-esp product between nodes of the molecular surface. This procedure was derived from the GRIND method as described by Pastor et al.[48] and the MACC equations are listed below.

Maximum auto-correlations (*MAC*):

$$MAC_{r_o}{}^{Curvature} = max\left[\left\{\left|\delta_{ij} \times \rho_i^{curv} \times \rho_j^{curv}\right|\right\}_{i=1\ldots(N-1);j=(i+1)\ldots N}\right] \quad \textbf{(Eq. S1)}$$

$$MAC_{r_o}{}^{ESP} = max\left[\left\{\left|\delta_{ij} \times \rho_i^{ESP} \times \rho_j^{ESP}\right|\right\}_{i=1\ldots(N-1);j=(i+1)\ldots N}\right] \quad \textbf{(Eq. S2)}$$

Maximum cross-correlations (MCC):

$$MCC_{r_o}{}^{Curv-ESP} = max\left[\left\{\left|\delta_{ij} \times \rho_i^{Curv} \times \rho_j^{ESP}\right|\right\}_{i=1\ldots N;j=1\ldots N}\right] \quad \textbf{(Eq. S3)}$$

where δ_{ij} is the distance between the nodes *i* and *j*, ρ_i^{curv}, ρ_j^{curv}, ρ_i^{ESP}, ρ_j^{ESP}, are the values of the curvature of the vdW surface and electrostatic potential (ESP) at the nodes *i* and *j* on the molecular surface with a total number of node *N* with r_o as the distance bins calculated from the minimum to maximum distance

between nodes. This quantum-mechanical derived descriptor allows for the independence of alignment between molecules because the distance between features is not dependent on orientation or directionality. Thus, each maximum product represents the interaction between nodes at the molecular surface and the combined maximum products at each distance bin form the correlogram.

Genetic Algorithm Feature Selection

Once descriptors for all 124 molecules in the set were prepared they had to be partitioned into a training set to build the QSAR model and a test set to test its predictive potential. The training set is comprised of 84 compounds (68% of the total set) and the test set is comprised of 40 compounds (32% of the total set). The training and test sets were chosen at random but were ensured to contain the same ratio of active to inactive molecules. Partial least square regression (PLSR) models[49] were calibrated using the GRIND descriptors of the training set. The number of descriptors from this method was quite high with ~150 descriptors for each molecule. The number of features (variables in the linear equation) needs to be small enough such that over-fitting does not occur. Thus, PLSR alone can produce poor results and an additional feature selection step must be implemented. To this effect a genetic algorithm (GA)[50] was utilized to optimize the descriptors to reduce the number with which to build the QSAR model. Instead of predicting the absolute IRI activity value, we trained PLSR models with the GRIND descriptors to classify whether an additive has IRI activity lower (active) or higher (inactive) than 70%. The fitness function used for the GA in this project was an *F-score* of cross-validation receiver-operator-curve (ROC) defined as the harmonic mean of the precision and sensitivity in Eq. S4,

$$F - score = 2 \cdot \frac{precision \cdot sensitivity}{precision + sensitivity} \qquad \textbf{(Eq S4)}$$

where precision is equal to the ratio of true positives over the sum of true positives and false positives (TP/(TP + FP)) and sensitivity is the ratio of true positives over the sum of true positives plus false negatives (TP/(TP + FN). The cut-off value yielding the maximum value of the harmonic mean of the precision and sensitivity (F-score in Eq. S4) was named harmonic cut-off.

In cross validation, the training set of 84 molecules is partitioned into small subsets where the whole QSAR analysis is performed. This is done multiple times to reduce variability and gain confidence in the model. The subsets are randomly chosen and include molecules only from the training sets. For this specific project, an exhaustive leave-one-out (LOO) cross-validation was performed.

In the GA optimization, initially a population of 100 binary individual models was randomly selected for the first generation. Mating yielded a 90% offspring success during evolution and mutation had a 10% offspring success. A total of 100 independent GA runs were performed with a cutoff of 1000 generation or when 90% of the generations reached the same fitness score. This implementation was written in python using a generic GA implementation with the NIPALS algorithm for PLSR analysis.[51]

Optimum QSAR Model

The optimum QSAR model was found with to be only 23 descriptors to classify whereby the IRI activities were classified as either active (% MGS < 70) and inactive (% MGS > 70). In cross-validation experiments, it was found that this model successfully identified 80% of the IRI active compounds in the training set (the compounds used to make the model) with precision of 0.8. Next, the same procedure was performed on the remaining 37 compounds (test set) and consistent performance was shown with 83% of active molecules were successfully identified with a similar precision of 0.8.

The sensitivity of the optimum QSAR models at the harmonic cut-off value was found to be above 0.8 for training and cross-validation; and of 0.67 for the test sets. For specificity, only the test set (specificity = 0.72) had a value below 0.8. Finally, precision yielded good results for training (0.89), satisfactory results for cross-validation (0.78), and lower value for the test set (0.64). But the ROC plots for the training set and test sets shown below in Figure 3.6, illustrated both good AUC values for training and test sets were 0.834 and 0.830 respectively.

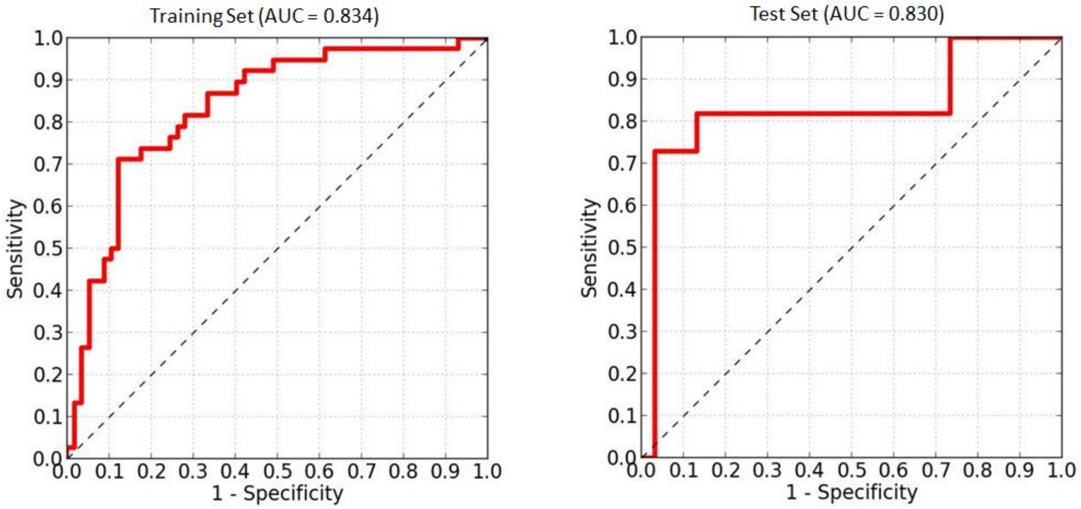

Figure S2: Area under the curve (AUC) plots for the training set and test sets. This is a graph of sensitivity vs. specificity and provides the probability that the model will properly classify molecules based on their activity.

References

1. Knight, C. A., Hallett, J. & DeVries, A. L. Solute effects on ice recrystallization: An assessment technique. *Cryobiology.* **25**, 55-60 (1988).
2. Jackman, J., Noestheden, M., Moffat, D., Pezacki, J. P., Findlay, S. & Ben, R. N. Assessing antifreeze activity of AFGP 8 using domain recognition software. *Biochem. Bioph. Res. Co.* **354**, 340-344 (2007).
3. McGill, N. W. & Williams, S. J. 2,6-Disubstituted Benzoates As Neighboring Groups for Enhanced Diastereoselectivity in β-Galactosylation Reactions: Synthesis of β-1,3-Linked Oligogalactosides Related to Arabinogalactan Proteins. *J. Org. Chem*. **74**, 9388-9398 (2009).
4. Smits, E., Engberts, J. B. F. N., Kellogg K. M. & Van Doren, H. A. Reliable method for the synthesis of aryl [small beta]-D-glucopyranosides, using boron trifluoride-diethyl ether as catalyst. *J. Chem. Soc. Perk. T.* **1**, 2873-2877 (1996).
5. Alberti, A., *et al*. Reactivity of glucosyl radical in the presence of phenols. *Tetrahedron*. **52**, 10241-10248 (1996).
6. Schwizer, D., *et al*. Antagonists of the myelin-associated glycoprotein: A new class of tetrasaccharide mimics. *Bioorgan. Med. Chem*. **14**, 4944-4957 (2006).
7. Li, Y., Mo, H., Lian, G. & Yu, B. Revisit of the phenol O-glycosylation with glycosyl imidates, $BF_3 \cdot OEt_2$ is a better catalyst than TMSOTf. *Carb. Res*. **363**, 14-22 (2012).
8. Aich, U. & Loganathan, D. Stereoselective single-step synthesis and X-ray crystallographic investigation of acetylated aryl 1,2-trans glycopyranosides and aryl 1,2-cis C2-hydroxy-glycopyranosides. *Carb. Res.* **341**, 19-28 (2006).
9. Li, Z. J., Cai, L. N. & Cai, M. S. Studies on Glycosides. X. An Alternate Method For Highly Stereoselective Synthesis of Alkyl-β-D-glucopyranosides. *Synthetic Commun*. **22**, 2121-2124 (1992).
10. Desai, R. N. & Blackwell, L. F. TEMPO-Mediated Regiospecific Oxidation of Glucosides to Glucuronides. *Synlett*. **2003**, 1981-1984 (2003).
11. Hansen, T. N., Smith, K.M., & Brockbank, K.G.M. Type I antifreeze protein attenuates cell recoveries following cryopreservation. *Transplant. P*. **25**, 3182-3184 (1993).

12. Yu, J., *et al.* Novel NMR Platform for Detecting Gene Transfection: Synthesis and Evaluation of Fluorinated Phenyl β-D-Galactosides with Potential Application for Assessing LacZ Gene Expression. *Bioconjugate Chem.* **15**, 1334-1341 (2004).
13. Rye, C. S. & Withers, S. G. Elucidation of the Mechanism of Polysaccharide Cleavage by Chondroitin AC Lyase from Flavobacterium heparinum. *J. Am. Chem. Soc*. **124**, 9756-9767 (2002).
14. Soulère, L., *et al.* Synthesis of GTP-Derived Ras Ligands. *ChemBioChem.* **5**, 1448-1453 (2004).
15. Mori, T., Fujita, S. & Okahata, Y. Transglycosylation in a two-phase aqueous-organic system with catalysis by a lipid-coated β-D-galactosidase. *Carb. Res*. **298**, 65-73 (1997).
16. Clerici, F., Gelmi, M. L. & Mottadelli, S. Glycosides. Part 1. New synthesis of 1,2-trans O-aryl glycosides, via tributyltin phenoxides. *J. Chem. Soc. Perk. T.* **1**, 985-988 (1994).
17. Xue, S. T., *et al*. Synthesis and Anti-Influenza Virus Activities of a Novel Class of Gastrodin Derivatives. *Molecules*. **18**, 3789-3805 (2013).
18. Liu, H. M., Yan, X., Li, W. & Huang, C. A mild and selective method for cleavage of O-acetyl groups with dibutyltin oxide. *Carb. Res.* **337**, 1763-176 (2002).
19. Dasgupta, S., Rajput, V. K., Roy, B. & Mukhopadhyay, B. Lanthanum Trifluoromethane-sulfonate-Catalyzed Facile Synthesis of Per-O-acetylated Sugars and Their One-Pot Conversion to S-Aryl and O-Alkyl/Aryl Glycosides. *J. Carbohyd. Chem*. **26**, 91-106 (2007).
20. Lopez, R. & Fernandez-Mayoralas, A. Enzymic β-Galactosidation of Modified Monosaccharides: Study of the Enzyme Selectivity for the Acceptor and Its Application to the Synthesis of Disaccharides. *J. Org. Chem*. **59**, 737-745 (1994).
21. Mukhopadhyay, B., Kartha, K. P. R., Russell, D. A. & Field, R. A. Streamlined Synthesis of Per-O-acetylated Sugars, Glycosyl Iodides, or Thioglycosides from Unprotected Reducing Sugars. *J. Org. Chem.* **69**, 7758-7760 (2004).
22. Saksena, R., Zhang, J. & Kovac, P. Synthesis of 2-(Trimethylsilyl)ethyl α-D-Mannopyranosides Revisited. *J. Carbohyd. Chem*. **21**, 453-470 (2002).
23. Garegg, P. J., Kvarnström, I., Niklasson, A., Niklasson, G. & Svensson, S. C. T. Partial Substitution of Thioglycosides by Phase Transfer Catalyzed Benzoylation and Benzylation. *J. Carbohyd. Chem*. **12**, 933-953 (1993).
24. Crich, D. & Sun, S. Direct chemical synthesis of β-mannopyranosides and other glycosides via glycosyl triflates. *Tetrahedron*. **54**, 8321-8348 (1998).
25. Balthaser, B. R. & McDonald F. E. Brønsted Acid-Promoted Glycosylations of Disaccharide Glycal Substructures of the Saccharomicins. *Org. Lett*. **11**, 4850-4853 (2009).
26. Zheng, S., *et al.* Synthesis and biological profiling of tellimagrandin I and analogues reveals that the medium ring can significantly modulate biological activity. *Org. Biomol. Chem*. **10**, 2590-2593 (2012).
27. Bridiau, N., Benmansour, M., Legoy, M. D. & Maugard, T. One-pot stereoselective synthesis of β-N-aryl-glycosides by N-glycosylation of aromatic amines: application to the synthesis of tumor-associated carbohydrate antigen building blocks. *Tetrahedron.* **63**, 4178-4183 (2007).
28. Gantt, R. W., Peltier-Pain, P., Cournoyer, W. J., & Thorson, J. S. Using simple donors to drive the equilibria of glycosyltransferase-catalyzed reactions. *Nat. Chem. Biol*. **7**, 685-691 (2011).
29. Czechura, P., Tam, R. Y., Dimitrijevic, E., Murphy, A. V. & Ben, R. N. The Importance of Hydration for Inhibiting Ice Recrystallization with C-Linked Antifreeze Glycoproteins. *J. Am. Chem. Soc.* **130**, 2928-2929 (2008).

30. Santra, A., Sau, A. & Misra, A. K. Synthesis of Thioglycosides in Room Temperature Ionic Liquid. *J. Carbohyd. Chem*. **30**, 85-93 (2011).
31. Daragics, K. & Fügedi, P. Regio- and chemoselective reductive cleavage of 4,6-O-benzylidene-type acetals of hexopyranosides using BH_3·THF–TMSOTf. *Tetrahedron Lett.* **50**, 2914-2916 (2009).
32. Xu, W., Springfield, S. A. & Koh, J. T. Highly efficient synthesis of 1-thioglycosides in solution and solid phase using iminophosphorane bases. *Carb. Res*. **325**, 169-176 (2000).
33. Hanessian, S. *et al.* Practical syntheses of B disaccharide and linear B type 2 trisaccharide—non-primate epitope markers recognized by human anti-α-Gal antibodies causing hyperacute rejection of xenotransplants. *Tetrahedron.* **57**, 3267-3280 (2001).
34. Damkaci, F. & DeShong, P. Stereoselective Synthesis of α- and β-Glycosylamide Derivatives from Glycopyranosyl Azides via Isoxazoline Intermediates. *J. Am. Chem. Soc*. **125**, 4408-4409 (2003).
35. Dasgupta, S. & Nitz, M. Use of N,O-Dimethylhydroxylamine As an Anomeric Protecting Group in Carbohydrate Synthesis. *J. Org. Chem*. **76**, 1918-1921 (2011).
36. Grayson, E. J. *et al*. Glycosyl Disulfides: Novel Glycosylating Reagents with Flexible Aglycon Alteration. *J. Org. Chem.* **70**, 9740-9754 (2005).
37. Peltier, R., *et al*. Synthesis and antifreeze activity of fish antifreeze glycoproteins and their analogues. *Chem. Sci*. **1**, 538-551 (2010).
38. Piispanen, P., Persson, M., Claesson, P. & Norin, T. Surface properties of surfactants derived from natural products. Part 1: Syntheses and structure/property relationships—Solubility and emulsification. *J. Surfactants Deterg*. **7**, 147-159 (2004).
39. Arévalo, M. a. J., *et al.* Optically active sugar thioamides from δ-gluconolactone. *Tetrahedron-Asymmetr*. **11**, 1985-1995 (2000).
40. Svenson, S., Kirste, B. & Furhop, J. H. A CPMAS ^{13}C NMR Study of Molecular Conformations and Disorder of N-Octylhexonamides in Microcrystals and Supramolecular Assemblies. *J. Am. Chem. Soc.* **116**, 11969-11975 (1994).
41. Svenson, S., Schafer, A. & Furhop, J. H. Conformational effects of 1,3-syn-diaxial repulsion and 1,2-gauche attraction between hydroxy groups in monomolecular N-octyl-D-hexonamide solutions. A ^{13}C and ^{1}H NMR spectroscopic study. *J. Chem. Soc. Perk. T.* **2**, 1023-1028 (1994).
42. Spartan'14. *Wavefunction Inc*. (2015).
43. Halgren, T. A. Merck molecular force field. I. Basis, form, scope, parameterization, and performance of MMFF94. *J. Comput. Chem*. **17**, 490-519 (1996).
44. Stewart, J. P. Optimization of parameters for semiempirical methods V: Modification of NDDO approximations and application to 70 elements. *J. Mol. Model*. **13**, 1173-1213 (2007).
45. Frisch, M., *et al.* Gaussian 09, Revision A. 02, *Gaussian. Inc., Wallingford, CT 200* (2009).
46. Walker, P. D., Arteca, G. A. & Mezey, P. G. A complete shape characterization for molecular charge densities represented by Gaussian-type functions. *J. Comput. Chem.* **12**, 220-230 (1991).
47. Bondi, A. van der Waals Volumes and Radii. *J. Phys. Chem*. **68**, 441-451 (1964).
48. Fontaine, F., Pastor, M. & Sanz, F. Incorporating molecular shape into the alignment-free Grid-Independent Descriptors. *J. Med. Chem*. **47**, 2805-2815 (2004).
49. Geladi, P. & Kowalski, B. R. Partial least-squares regression: a tutorial. *Anal. Chim. Acta.* **185**, 1-17 (1986).
50. Holland, J. H. in *Adaptation in Natural and Artificial Systems* (University of Michigan Press, 1975).
51. Lorber, A., Wangen, L. E. & Kowalski, B. R. A theoretical foundation for the PLS algorithm. *J. Chemometr*. **1**, 19-31 (1987).
